# Supplementary material for: Accuracy of self-diagnosis in conditions commonly managed in primary care: diagnostic accuracy systematic review and meta-analysis
Source: BMJ Open. 2023 Jan 10;13(1):e065748. doi: 10.1136/bmjopen-2022-065748 (PMC9835960; doi:10.1136/bmjopen-2022-065748)
Supplement: Supplementary data [file bmjopen-2022-065748supp001.pdf]

SUPPLEMENTARY MATERIAL

Supplementary Table 1: Commonly managed conditions in primary care

As reported by Cooke et al 2013 (Cooke G, Valenti L, Glasziou P, Britt H. Common general practice presentations and publication frequency. Australian Family Physician. 2013; 42:65-8)

1	Hypertension
2	Immunisation/vaccination: all
3	Acute upper respiratory tract infection
4	Depression
5	Diabetes: nongestational
6	Lipid disorders
7	General check-up
8	Osteoarthritis
9	Back complaint
10	Prescription
11	Oesophagus disease
12	Female genital check-up
13	Acute bronchitis/bronchiolitis
14	Asthma
15	Anxiety
16	Test results
17	Urinary tract infection
18	Dermatitis, contact/allergic
19	Pregnancy
20	Sleep disturbance
21	Sinusitis acute/chronic
22	Gastroenteritis
23	Vitamin/nutritional deficiency

24	Malignant neoplasm of skin
25	Abnormal test results
26	Atrial fibrillation/flutter
27	Oral contraception
28	Solar keratosis/sunburn
29	Ischaemic heart disease
30	Viral disease, not otherwise specified

Supplementary Table 2: Search strategy

**Medline**	
**#**	**Searches**
1	Diagnostic Self Evaluation/
2	((self* adj diagnos*) or selfdiagnos*).ti,ab.
3	(self* and diagnos*).ti.
4	((self* adj test*) or selftest*).ti,ab.
5	(home adj3 diagnos*).ti,ab.
6	((selfreport* or self-report*) and diagnos*).ti.
7	((selfreport* or self-report*) adj5 diagnos*).ti,ab.
8	(diagnos* and (selftreat* or self-treat*)).ti,ab.
9	1 or 2 or 3 or 4 or 5 or 6 or 7 or 8
10	respiratory tract infections/ or common cold/ or influenza, human/ or laryngitis/ or exp pharyngitis/ or rhinitis/ or exp sinusitis/ or exp supraglottitis/ or tracheitis/ or exp otitis media/
11	(((respirat* or airway*) adj2 infection*) or (common cold or influenza or flu or pharyngitis or laryngitis or tonsillitis or sore throat or rhinitis or nasopharyngitis or nasolaryngitis or sinusitis or supraglottitis or epiglottitis or tracheitis or urti or otitis media)).ti,ab.
12	exp Depressive Disorder/ or Depression/
13	(depress* or (mood adj2 disorder*)).ti,ab.
14	diabetes mellitus/ or exp diabetes mellitus, type 1/ or exp diabetes mellitus, type 2/
15	diabet*.ti,ab.
16	dyslipidemias/ or exp hyperlipidemias/
17	(dyslipid?emia? or hyperlipid?emia? or hypercholesterol?emia? or cholesterol* or triglyceride*).ti,ab.
18	hypertension/
19	(hypertens* or high blood pressure).ti,ab.
20	exp Osteoarthritis/
21	(osteoarthritis or osteo-arthritis or (degenerat* adj2 arthritis)).ti,ab.
22	back pain/ or low back pain/
23	Sciatica/
24	Intervertebral Disc Displacement/
25	((back adj2 (pain or problem? or disorder?)) or slipped disc* or sciatica).ti,ab.
26	Esophageal Diseases/ or esophageal motility disorders/ or exp gastroesophageal reflux/ or Barrett Esophagus/
27	((oesophag* or esophag*) adj2 disease).ti,ab.
28	(((oesophag* or esophag* or gastr*) adj2 reflux) or heartburn or heart burn).ti,ab.
29	((barrett* or globus) adj2 (esophag* or oesophag*)).ti,ab.
30	exp Asthma/
31	asthma*.ti,ab.

32	exp Anxiety Disorders/ or Anxiety/
33	(anxiety or phobia*).ti,ab.
34	exp Urinary Tract Infections/
35	exp Cystitis/
36	((urin* adj3 infection*) or bacteriuria or pyuria or cystitis).ti,ab.
37	exp Vaginitis/
38	(vaginitis or vaginosis).ti,ab.
39	dermatitis/ or exp dermatitis, contact/ or eczema/
40	(dermatitis or eczema).ti,ab.
41	exp Sleep Wake Disorders/
42	exp Sleep Apnea Syndromes/
43	((sleep adj2 (disorder* or disturbance*)) or insomnia*).ti,ab.
44	(sleep adj2 (apnea or apnoea)).ti,ab.
45	restless leg*.ti,ab.
46	Gastroenteritis/ or diarrhea/ or vomiting/
47	(gastroenteritis or (stomach adj2 (bug? or upset))).ti,ab.
48	(diarrhoea or diarrhea or food poisoning).ti,ab.
49	Sunburn/
50	(sunburn or solar keratosis).ti,ab.
51	or/10-50
52	9 and 51
53	exp "Sensitivity and Specificity"/
54	exp "REPRODUCIBILITY OF RESULTS"/
55	(sensitiv* or specific* or predict* or accura* or valid* or reproduc*).ti,ab.
56	53 or 54 or 55
57	52 and 56
**Embase**	
**#**	**Searches**
1	*Self Evaluation/
2	((self* adj diagnos*) or selfdiagnos*).ti,ab.
3	(self* and diagnos*).ti.
4	((self* adj test*) or selftest*).ti,ab.
5	(home adj3 diagnos*).ti,ab.
6	((selfreport* or self-report*) and diagnos*).ti.
7	((selfreport* or self-report*) adj5 diagnos*).ti,ab.
8	(diagnos* and (selftreat* or self-treat*)).ti,ab.
9	1 or 2 or 3 or 4 or 5 or 6 or 7 or 8
10	respiratory tract infection/ or upper respiratory tract infection/ or exp respiratory tract inflammation/ or influenza/ or otitis media/
11	(((respirat* or airway*) adj2 infection*) or (common cold or influenza or flu or pharyngitis or laryngitis or tonsillitis or sore throat or rhinitis or nasopharyngitis or nasolaryngitis or sinusitis or supraglottitis or epiglottitis or tracheitis or urti or otitis media)).ti,ab.

12	exp Depression/
13	(depress* or (mood adj2 disorder*)).ti,ab.
14	diabetes mellitus/ or exp diabetes mellitus, type 1/ or exp diabetes mellitus, type 2/
15	diabet*.ti,ab.
16	dyslipidemia/ or exp hyperlipidemia/
17	(dyslipid?emia? or hyperlipid?emia? or hypercholesterol?emia? or cholesterol* or triglyceride*).ti,ab.
18	hypertension/
19	(hypertens* or high blood pressure).ti,ab.
20	exp Osteoarthritis/
21	(osteoarthritis or osteo-arthritis or (degenerat* adj2 arthritis)).ti,ab.
22	backache/ or discogenic pain/ or low back pain/
23	Sciatica/
24	intervertebral disk hernia/ or intervertebral disk disease/
25	((back adj2 (pain or problem? or disorder?)) or slipped disc* or sciatica).ti,ab.
26	exp gastroesophageal reflux/ or barrett esophagus/ or esophagus disease/
27	((oesophag* or esophag*) adj2 disease).ti,ab.
28	(((oesophag* or esophag* or gastr*) adj2 reflux) or heartburn or heart burn).ti,ab.
29	((barrett* or globus) adj2 (esophag* or oesophag*)).ti,ab.
30	exp Asthma/
31	asthma*.ti,ab.
32	exp Anxiety Disorder/
33	(anxiety or phobia*).ti,ab.
34	exp Urinary Tract Infection/
35	Cystitis/
36	((urin* adj3 infection*) or bacteriuria or pyuria or cystitis).ti,ab.
37	vagina discharge/ or exp vaginitis/
38	(vaginitis or vaginosis).ti,ab.
39	dermatitis/ or contact dermatitis/ or exp eczema/
40	(dermatitis or eczema).ti,ab.
41	sleep disorder/ or exp insomnia/ or periodic limb movement disorder/
42	exp sleep disordered breathing/
43	((sleep adj2 (disorder* or disturbance*)) or insomnia*).ti,ab.
44	(sleep adj2 (apnea or apnoea)).ti,ab.
45	restless leg*.ti,ab.
46	Gastroenteritis/ or diarrhea/ or vomiting/
47	(gastroenteritis or (stomach adj2 (bug? or upset))).ti,ab.
48	(diarrhoea or diarrhea or food poisoning).ti,ab.
49	Sunburn/
50	(sunburn or solar keratosis).ti,ab.
51	or/10-50

52	9 and 51
53	"Sensitivity and Specificity"/
54	diagnostic accuracy/ or diagnostic test accuracy study/
55	predictive validity/ or predictive value/ or reproducibility/
56	(sensitiv* or specific* or predict* or accura* or valid* or reproduc*).ti,ab.
57	53 or 54 or 55 or 56
58	52 and 57
**Cochrane**	
**ID**	**Search**
#1	MeSH descriptor: [Diagnostic Self Evaluation] explode all trees
#2	(((self* AND diagnos*) or selfdiagnos*)):ti OR (((self* NEXT diagnos*) or selfdiagnos*)):ti,ab,kw OR (((self* NEXT test*) or selftest*)):ti,ab,kw OR (home NEAR/3 diagnos*):ti,ab,kw
#3	(((selfreport* or self-report*) and diagnos*)):ti OR (((selfreport* or self-report*) NEAR diagnos*)):ti,ab,kw OR ((diagnos* and (selftreat* or self-treat*))):ti,ab,kw
#4	#1 OR #2 OR #3
#5	MeSH descriptor: [Sensitivity and Specificity] explode all trees
#6	MeSH descriptor: [Reproducibility of Results] explode all trees
#7	(sensitiv* or specific* or predict* or accura* or valid* or reproduc*):ti,ab,kw
#8	#5 OR #6 OR #7
#9	#4 AND #8
**Cinahl**	
**#**	**Query**
S39	S3 AND S35 AND S38
S38	S36 OR S37
S37	TX sensitiv* or specific* or predict* or accura* or valid* or reproduc*
S36	(MH "Sensitivity and Specificity") OR (MH "Predictive Value of Tests")
S35	(S4 OR S5 OR S6 OR S7 OR S8 OR S9 OR S10 OR S11 OR S12 OR S13 OR S14 OR S15 OR S16 OR S17 OR S18 OR S19 OR S20 OR S21 OR S22 OR S23 OR S24 OR S25 OR S26 OR S27 OR S28 OR S29 OR S30 OR S31 OR S32 OR S33 OR S34)
S34	TI (sunburn or "solar keratosis") OR AB (sunburn or "solar keratosis")
S33	(MH "Sunburn")
S32	TI ((gastroenteritis or (stomach N2 (bug or bugs or upset)))) OR AB ((gastroenteritis or (stomach N2 (bug or bugs or upset)))) OR TI (diarrhoea or diarrhea or "food poisoning") OR AB (diarrhoea or diarrhea or "food poisoning")
S31	(MH "Gastroenteritis") OR (MH "Vomiting") OR (MH "Diarrhea")
S30	TI (((sleep N2 (disorder* or disturbance*)) or insomnia*)) OR AB (((sleep N2 (disorder* or disturbance*)) or insomnia*)) OR TI ((sleep N2 (apnea or apnoea))) OR AB ((sleep N2 (apnea or apnoea))) OR TI "restless legs" OR AB "restless legs"
S29	(MH "Sleep Disorders, Intrinsic+")
S28	TI (dermatitis or eczema) OR AB (dermatitis or eczema)
S27	(MH "Dermatitis") OR (MH "Dermatitis, Contact+") OR (MH "Eczema")
S26	TI (vaginitis or vaginosis) OR AB (vaginitis or vaginosis)

S25	(MH "Vaginitis+")
S24	TI (((urin* N3 infection*) or bacteriuria or pyuria or cystitis)) OR AB (((urin* N3 infection*) or bacteriuria or pyuria or cystitis))
S23	(MH "Urinary Tract Infections+") OR (MH "Cystitis")
S22	TI asthma* OR AB asthma*
S21	(MH "Asthma+")
S20	TI (((oesophag* or esophag*) N2 disease)) OR (((oesophag* or esophag*) N2 disease)) OR TI ((((oesophag* or esophag* or gastr*) N2 reflux) or heartburn or "heart burn")) OR AB ((((oesophag* or esophag* or gastr*) N2 reflux) or heartburn or "heart burn")) OR (((barrett* or globus) N2 (esophag* or oesophag*))) OR (((barrett* or globus) N2 (esophag* or oesophag*)))
S19	(MH "Gastroesophageal Reflux") OR (MH "Esophageal Motility Disorders") OR (MH "Esophageal Diseases") OR (MH "Barrett Esophagus")
S18	TI (((back N2 (pain or problem* or disorder*)) or "slipped disc*" or sciatica)) OR AB (((back N2 (pain or problem* or disorder*)) or "slipped disc*" or sciatica))
S17	(MH "Low Back Pain") OR (MH "Back Pain") OR (MH "Sciatica") OR (MH "Intervertebral Disk Displacement")
S16	TI ((osteoarthritis or osteo-arthritis or (degenerat* N2 arthritis))) OR AB ((osteoarthritis or osteo-arthritis or (degenerat* N2 arthritis)))
S15	(MH "Osteoarthritis+")
S14	TI (hypertens* or "high blood pressure") OR AB (hypertens* or "high blood pressure")
S13	(MH "Hypertension")
S12	TI (dyslipidemia* or hyperlipidemia* or hypercholesterolemia* or dyslipidaemia* or hyperlipidaemia* or hypercholesterolaemia* or cholesterol* or triglyceride*) OR AB (dyslipidemia* or hyperlipidemia* or hypercholesterolemia* or dyslipidaemia* or hyperlipidaemia* or hypercholesterolaemia* or cholesterol* or triglyceride*)
S11	(MH "Hyperlipidemia+")
S10	TI diabet* OR AB diabet*
S9	(MH "Diabetes Mellitus") OR (MH "Diabetes Mellitus, Type 1") OR (MH "Diabetes Mellitus, Type 2")
S8	TI ((depress* OR anxiety or (mood N2 disorder*))) OR AB ((depress* OR anxiety or (mood N2 disorder*)))
S7	(MH "Depression") OR (MH "Anxiety")
S6	TI ((((respirat* or airway*) N2 infection*) or ("common cold" or influenza or flu or pharyngitis or laryngitis or tonsillitis or "sore throat" or rhinitis or nasopharyngitis or nasolaryngitis or sinusitis or supraglottitis or epiglottitis or tracheitis or urti or "otitis media"))) OR AB ((((respirat* or airway*) N2 infection*) or ("common cold" or influenza or flu or pharyngitis or laryngitis or tonsillitis or "sore throat" or rhinitis or nasopharyngitis or nasolaryngitis or sinusitis or supraglottitis or epiglottitis or tracheitis or urti or "otitis media")))
S5	(MH "Otitis Media+")
S4	(MH "Respiratory Tract Infections") OR (MH "Common Cold") OR (MH "Influenza") OR (MH "Influenza, Human+") OR (MH "Laryngitis+") OR (MH "Pharyngitis") OR (MH "Rhinitis+") OR (MH "Sinusitis+") OR (MH "Tonsillitis+")

S3	S1 OR S2
S2	TI (((self* AND diagnos*) or selfdiagnos*)) OR AB (((self* N1 diagnos*) or selfdiagnos*)) OR TI (((self* N1 test*) or selftest*)) OR AB (((self* N1 test*) or selftest*)) OR TI (home N3 diagnos*) OR AB (home N3 diagnos*) OR TI (((selfreport* or self-report*) and diagnos*)) OR AB (((selfreport* or self-report*) N5 diagnos*)) OR TI ((diagnos* and (selftreat* or self-treat*))) OR AB ((diagnos* and (selftreat* or self-treat*)))
S1	(MH "Self Diagnosis")

Supplementary table 3: Data items included in extraction sheet (where available)

Study identification - author, year, location
Study research question
Study design and setting
Study funding source
Target condition definition/diagnostic criteria
Participant characteristics and numbers, including exclusions
Index test
Reference standard
Flow of participants through study including losses to follow-up<br>Patient presentation and prior testing
Conduct of the study including timing of the tests, and information on masking
Absolute counts of true positive (TP), false positive (FP), false negative (FN) and true negative (TN) diagnoses.
Statistical analyses that were performed, including whether all participants were included in analyses
Additional summary information on participant preference, timing, or cost, as available.

Supplementary table 4: Protocol

Accuracy of self-diagnosis in conditions commonly managed in primary care: diagnostic accuracy review

Annette Plüddemann [1], Hayley E Jones [2], Carl Heneghan [1]

1. Nuffield Department of Primary Care Health Sciences, University of Oxford, OX2 6GG
2. Population Health Sciences, Bristol Medical School, University of Bristol, BS8 2PS

Background and rationale

A wide range of conditions present to primary care, some acute, some chronic. As a consequence primary care is facing increasing workload (Hobbs et al 2016) that may become unmanageable.

Some common conditions in primary care, therefore, have the potential to be self-diagnosed and self-treated by the patients themselves. This offers superior convenience for individuals, swifter diagnosis and treatment where relevant, reduced costs for health service providers and
potentially reduce the burden on primary care services. Self-diagnosis may apply to initial diagnosis, or to diagnosing an exacerbation of an ongoing condition, such as an exacerbation of chronic obstructive pulmonary disease (COPD). In order to support self-diagnosis where safe and appropriate, the efficacy of self-diagnosis needs to be assessed, and this information used to make evidence-based decisions on who can self-diagnose safely.

Where there is an available comparison with diagnosis by a healthcare professional, it is possible to assess the accuracy of self-diagnosis. A further comparison could potentially be made with diagnosis by an allied healthcare professional such as pharmacists, but this is outside the scope of this current review. Sometimes, self-diagnosis involves using a diagnostic test (any type of medical test used to help diagnose or detect disease). For conditions where this is available and appropriate, the accuracy of these tests also informs the safety and efficacy of self-diagnosis by the patient.

Previous studies on the safety and accuracy of self-diagnosis of conditions commonly managed in primary care setting include self-diagnosis of urinary tract infection (Donofrio & Weiner 2013), high blood pressure (Tormo et al 2000) and depression (Hedayati et al 2006).

This review aims to identify, appraise and summarise the available evidence on self-diagnosis in common conditions in primary care. Cooke and colleagues recently reported the 30 most commonly managed conditions in primary care in Australia, which has a health landscape broadly comparable with western Europe (Cooke et al 2013). This list arises from survey data collected between January 2009 and December 2010, which included 194,100 patient encounters from 1,941 GPs. The most commonly managed conditions included some with the potential for self-diagnosis, e.g. urinary tract infection, as well as some that would be unsuitable for self-diagnosis, such as "general check-up". We base our review on conditions

from this list that are relevant for self-diagnosis. We may review infectious diseases and non-communicable diseases separately.

Objectives

Primary objective

Our primary objective is to summarize the accuracy of self-diagnosis of common conditions in primary care, compared with diagnosis by a healthcare provider.

Secondary objective

To summarise any associated relevant information relating to self-diagnosis of common conditions in primary care, such as information on patient preference, timing, or cost (only using information from studies we include for accuracy data). Where there is substantial qualitative information reported, this will only be summarised briefly; detailed qualitative approaches will not be used.

Methods

Criteria for considering studies for this review

Types of studies

Prospective or retrospective studies comparing the results of self-diagnosis of common self-limiting conditions in primary care by free-living individuals, to the results of a reference standard test performed by a healthcare service provider, will be included. Studies with a case-control design will be excluded. In case of duplicate publications we will include the study report with the highest methodological quality. There will be no language restrictions.

We will exclude studies comparing self-diagnosis with diagnosis by allied health professionals such as a pharmacists.

Participants

Adults (>= 18 years of age) self-diagnosing conditions common in primary care.

Index tests

Index tests will be the self-testing or self-diagnosis of relevant conditions, compared with diagnosis by a healthcare practitioner.

Comparator tests

Comparator tests will comprise diagnosis by a healthcare practitioner.

Outcome measures

Diagnostic accuracy measures (e.g. sensitivity, specificity, likelihood ratios, predictive values, etc.) and primary data for 2x2 tables. Studies reporting only measures of agreement will be excluded.

Search methods to identify studies

Electronic searches

The search strategy will be developed in consultation with a healthcare librarian experienced with supporting systematic reviews. No language restrictions will be applied. The search strategy will use multiple electronic databases, from inception onwards including:

Medline

EMBASE

Cochrane Central Register of Controlled Trials (CENTRAL)

Trip database

Web of Science for conference proceedings, dissertations, and theses

World Health Organization International Clinical Trials Registry Platform (ICTRP),

ClinicalTrials.gov

Database of Abstracts of Reviews of Effect (DARE)

We will also search Science Citation Index Expanded for study reports that cite the included studies.

The search may use relevant filters, but in order to maximise sensitivity, will not be limited to these. The reference lists of relevant studies will be examined and additional tools such as the “related articles” feature in PubMed will also be used to identify relevant publications.

Data collection and analysis

Selection of studies

Two reviewers will independently apply the selection criteria to the titles and abstracts of the study reports identified by the searches. If the decision to exclude a study cannot be made on the basis of the title and the abstract, the full study report will be retrieved for inclusion assessment. The final decision on inclusion will be based on the full study report. Disagreements between reviewers will be resolved by discussion, or if necessary by a third reviewer. Study identification will be summarised in a PRISMA flow diagram.

Data extraction and management

Two reviewers will independently extract information from selected studies into a data extraction sheet. Disagreements will be resolved by discussion, or if necessary with the help of a third reviewer.

Where this is insufficient (or unclear) information, where there is an email address provided, the authors will be contacted via email for clarification. Where data is not available for completion of 2x2 tables, the studies will be excluded from the analysis.

Data to be extracted

The following information will be extracted from the included studies, where available:

Study identification - author, year, location

Study research question

Study design and setting

Target condition definition/diagnostic criteria

Participant characteristics and numbers, including exclusions

Index test

Reference standard

Flow of participants through study including losses to follow-up

Patient presentation and prior testing

Conduct of the study including timing of the tests, and information on masking

Absolute counts of true positive (TP), false positive (FP), false negative (FN) and true negative (TN) diagnoses.

Statistical analyses that were performed, including whether all participants were included in analyses

Additional summary information on participant preference, timing, or cost, as available.

Assessment of methodological quality

To assess methodological quality, we will use the Quality Assessment of Diagnostic Accuracy Studies-2 (QUADAS-2) tool (Whiting et al 2011). Two reviewers will independently assess studies' methodological quality; disagreements will be resolved by discussion, or if necessary, by a third reviewer. The QUADAS-2 tool facilitates assessment of bias in four areas: patient selection; index test; reference standard; flow and timing; and also facilitates assessment of applicability of the studies to the review research question.

The data will be presented in a tables showing risk of bias and applicability within each domain assessed for each study. These data will be considered in relation to interpreting the results of the studies.

Statistical analysis and data synthesis

Analyses will be conducted for each category of condition specified. Summary tables will detail study information including the patient sample, condition, study design, the test under evaluation, and the comparator.

Meta-analysis

For each test, RevMan will be used to produce paired forest plots to explore the between-study variability of sensitivity and specificity across the included studies. For each study estimate of sensitivity and specificity, corresponding 95% confidence intervals will be shown

to illustrate the uncertainty related to each study estimate. If accuracy has been reported at multiple common thresholds, forest plots will be sub-grouped on threshold.

Bivariate meta-analysis methods (Reitsma et al 2005) will be used to generate pooled estimates of sensitivity and specificity where sufficient data is available for each test or condition. These will be plotted with 95% confidence and prediction ellipses in Receiver Operating Characteristic (ROC) space. Where appropriate, summary ROC curves will also be plotted, drawing on the equivalence of the bivariate method and the hierarchical summary ROC meta-analysis model (Rutter and Gatsonis 2001; Harbord et al 2007). For these analyses, we will use WinBUGS or the metandi command in Stata, as appropriate, and feed parameters directly into Revman to produce Cochrane-standardised output.

Where appropriate, meta-analysis models that include multiple thresholds will be employed (e.g. Steinhauser et al 2016 or similar).

Investigating heterogeneity

For medical conditions for which data from more than one study are available, it may be possible to investigate heterogeneity in the results. Two approaches will be used to explore the sources of between-study heterogeneity: 1) inclusion of study level characteristics as covariates in the bivariate model (meta-regression) 2) carrying out sub-group analyses. These approaches will only be carried out if there is sufficient data available and sub-group specific pooled estimates are thought to be of clinical relevance. Any meta-regressions will be carried out using WinBUGS or the xtmelogit command in Stata.

Sensitivity analyses

If there appear to be any outliers in the data, these studies will be removed from the analysis to evaluate the impact on the overall pooled estimates.

Investigating reporting bias

Funnel plots used to detect publication bias in reviews of RCTs have been shown to be misleading for diagnostic test accuracy reviews (Deeks et al 2005; Leeflang et al 2008). Funnel plots as an assessment of reporting bias will therefore not be included in this review. Publication bias will be assessed using Deek’s test, as recommended by the Cochrane Handbook, where data allows (Deeks et al 2005).

Funding

This project is funded by National Institute for Health Research School for Primary Care Research (NIHR SPCR) [ProjectNumber 390]

Declarations of conflict of interest

Dr. Plüddemann reports grants from NIHR, grants from NIHR School of Primary Care Research, during the conduct of the study; and occasionally receives expenses for teaching Evidence-Based Medicine. Dr. Heneghan reports receiving expenses and fees for his media work. He has received expenses from the WHO and holds grant funding from the NIHR, the NIHR School of Primary Care Research, The Wellcome Trust and the WHO. He has received financial remuneration from an asbestos case. He has also received income from the

publication of a series of toolkit books published by Blackwells. On occasion, he receives expenses for teaching EBM and is also paid for his GP work in NHS out of hours. CEBM jointly runs the EvidenceLive Conference with the BMJ and the Overdiagnosis Conference with some international partners which are based on a non-profit making model.

References

Cooke G, Valenti L, Glasziou P, Britt H. Common general practice presentations and publication frequency. Aust Fam Physician. 2013;42(1-2):65-8.

Deeks JJ, Macaskill P, Irwig L. The performance of tests of publication bias and other sample size effects in systematic reviews of diagnostic test accuracy was assessed. J Clin Epidemiol. 2005;58(9):882-93

Donofrio JC, Weiner SG. Female patient self-diagnosis compared with emergency physician diagnosis of urinary tract infection. J Emerg Med. 2013;45(6):969-73. doi: 10.1016/j.jemermed.2013.07.019

Harbord RM, Deeks JJ, Egger M, Whiting P, Sterne JA. A unification of models for meta-analysis of diagnostic accuracy studies. Biostatistics. 2007 Apr;8(2):239-51.

Hedayati SS, Bosworth HB, Kuchibhatla M, Kimmel PL, Szczech LA. The predictive value of self-report scales compared with physician diagnosis of depression in hemodialysis patients. Kidney Int. 2006 May;69(9):1662-8.

Hobbs FDR, Bankhead C, Mukhtar T, Stevens S, Perera-Salazar R, Holt T, Salisbury C; National Institute for Health Research School for Primary Care Research. Clinical workload in UK primary care: a retrospective analysis of 100 million consultations in England, 2007–14. The Lancet; 387; 10035: 2323-2330

Leeflang MM, Deeks JJ, Gatsonis C, Bossuyt PM; Cochrane Diagnostic Test Accuracy Working Group. Systematic reviews of diagnostic test accuracy. Ann Intern Med. 2008; 16;149(12): 889-97

Rutter CM, Gatsonis CA. A hierarchical regression approach to meta-analysis of diagnostic test accuracy evaluations. Stat Med. 2001 Oct 15;20(19):2865-84.

Reitsma JB, Glas AS, Rutjes AW, Scholten RJ, Bossuyt PM, Zwinderman AH. Bivariate analysis of sensitivity and specificity produces informative summary measures in diagnostic reviews. J Clin Epidemiol. 2005 Oct;58(10):982-90

Review Manager (RevMan) [Computer program]. Version 5.3. Copenhagen: The Nordic Cochrane Centre, The Cochrane Collaboration, 2014

Steinhauser S, Schumacher M, Rücker G. Modelling multiple thresholds in meta-analysis of diagnostic test accuracy studies. BMC Med Res Methodol. 2016 Aug 12;16(1):97. doi: 10.1186/s12874-016-0196-1.

Tormo MJ, Navarro C, Chirlaque MD, Barber X. Validation of self diagnosis of high blood pressure in a sample of the Spanish EPIC cohort: overall agreement and predictive values. EPIC Group of Spain. J Epidemiol Community Health. 2000 Mar;54(3):221-6.

Supplementary Table 5: Characteristics of included studies

Disease/ condition	Author, year	Design	Setting	Country	Study duration	Number of participants	Mean age* (years)	Female (%)
Vaginal infection	Donders 2016	Prospective diagnostic accuracy study	Birth control, general gynaecology, infertility and prenatal clinics	Uganda	N/A	360	28.3	100
	Sungkar 2012	RCT (intervention arm only)	Prenatal clinics or hospitals	Indonesia	24 weeks	176	28	100
	Geva 2006	Prospective diagnostic accuracy study	Gynaecologic clinics	Israel	N/A	593	18 – 60 range	100
	Ryan-Wenger 2010	Prospective diagnostic accuracy study	Military clinics	USA	N/A	546	25.7	100
	Jones 2013	RCT (both arms)	Clinics or home	Brazil	N/A	695	18 – 40 range	100
Common skin condition	Bregnhoj 2011	Prospective cohort	Not reported	Denmark	18 months	502	17.5	95
	Svensson 2002	Prospective diagnostic accuracy study	Dermatology outpatient clinics	Sweden	N/A	208	40.4	50
	Elsner 2015	Prospective diagnostic accuracy study	Not reported	Germany & Austria	N/A	165	≥18 years	81
	Josefson 2011	Prospective diagnostic accuracy study	Hospital dermatology departments	Sweden	N/A	243	44	69
HIV	Assiimwe 2014	RCT (unsupervised arm only)	Home	Uganda	N/A	123	28 (23–32), median (IQR)	47
	Belete 2019	Cross-sectional	Public health facilities	Ethiopia	N/A	400	29 (17.7–40.3), median (IQR)	61
	Choko 2011	Cross-sectional	Home	Malawi	N/A	241	27 (NR) median (IQR)	52
	Choko 2015	RCT (intervention arm only)	Home	Malawi	2 years	2370	NR	NR

	Kapaku 2017	Cohort	Home/Voluntary counselling & testing facilities	Zambia	1 year	2572	26 (21–35), median (IQR)	59
	Kurth 2016	Cross-sectional	NR (not at home)	Kenya	N/A	240**	36	33
	Martinez Perez 2016	Cross-sectional	Health care clinics/HIV testing sites	South Africa	N/A	2205	Male: 27 (11-36) Female: 28 (22-36) median (IQR)	66
	Orasure 2012	Cross-sectional	Study site	USA	N/A	5798	NR	50
	Pant Pai 2013	Cross-sectional	Hospital	South Africa	N/A	251	≥18 years	79

RCT – randomised control trial; N/A – not applicable; NR – not reported, * unless otherwise indicated, ** Subset (N=113) that used laboratory reference standard included in systematic review and pooled analysis.

Supplementary Table 6: Characteristics of self-diagnosis (index) tests and reference standard tests

Disease/ condition	Author, year	Test for	Self-diagnosis test (index)	Self-diagnosis test threshold	Reference test	Reference test threshold	Interval between index & reference test	Data collection points
Vaginal infection	Donders 2016a	Bacterial vaginosis	Vaginal fluid test using pH strip	+/- (≥ 4.5 pH)	Air dried vaginal fluid test using gram staining (Nugent score) Assessment by central laboratory	+/- (≥ 4.5 pH, Nugent score 7-10)	NR	N/A
	Donders 2016b			+/- (≥ 4.7 pH)		+/- (≥ 4.7 pH, Nugent score 7-10)		
	Sungkar 2012	Bacterial vaginosis	Vaginal fluid test using pH strip	+/-	Vaginal fluid test using microbiological gram staining test (Kopeloff modified Gram stain) Prepared by midwives for laboratory assessment	+/-	NR	Baseline, 16 – 18, 18 – 20, 20 – 22, 22 – 24 weeks
	Geva 2006	Bacterial vaginosis and/or *Trichomonas vaginalis*	Vaginal discharge test using panty liner test kit (VI-SENSE)	+/- (based on strip colour, no level reported*)	Clinical diagnosis plus vaginal wall swabs testing pH (nitrazine paper), amine, culture (InPouch TV, BioMed Diagnostic) and gram staining (Nugent score). Assessment by board certified gynaecologists and central laboratory.	BV: >3: (a) homogeneous discharge, (b) pH value >4.5, (c) release of fishy odor (KOH was added to the vaginal discharge, and (d) presence of clue cells; or 7+ Nugent score of gram stain TV: +/- by culture	Within 6 hours	N/A
	Ryan-Wenger 2010	Bacterial vaginosis and/or *Trichomonas vaginalis*	Vaginal fluid test using Women in the military self-diagnosis kit	+/- (based on ≥ 4.7 pH, amines,	Clinical interview plus vaginal fluid test for pH (nitrazine paper, amines (FemExam card), whiff, wet mount	+/-	None	N/A

			(includes FemExam card)	vaginal itching)	microscopy (Affirm VPIII Microbial Identification Test) Assessment by women's health nurse practitioner plus in clinic microscopy testing			
	Ryan-Wenger 2010	*Candida* vaginitis	Vaginal fluid test using Women in the military self-diagnosis kit (includes FemExam card)	+/- (based on ≥ 4.7 pH, amines, vaginal itching)	Clinical interview plus vaginal fluid test for pH (nitrazine paper, amines (FemExam card), whiff, wet mount microscopy (Affirm VPIII Microbial Identification Test) Assessment by women's health nurse practitioner plus in clinic microscopy testing	+/-	None	N/A
	Jones 2013	*Trichomonas vaginalis*	Vaginal fluid testing using dipstick test (OSOM Trichomonas rapid test)	+/- (two red lines)	Vaginal fluid test using PCR test Assessment at central laboratory	+/-	None	N/A
Common skin condition	Bregnhoj 2011	Eczema	Questionnaire on presence of eczema	Positive response	Hand Eczema Severity Index (HECSI) Interpretation by clinician	+ve for presence of eczema	Same day	Inclusion & 18 month follow-up
	Svensson 2002	Hand eczema	Questionnaire on presence of eczema	Positive response	Hand examination for erythema, papules, vesicles, scaling, fissures, lichenification and hyperkeratotic areas. Assessment by experienced dermatologist	+ve if erythema and papules ⁄ vesicles OR erythema and scaling and fissures ⁄ lichenification	None	N/A
	Elsner 2015	Allergic reaction to nickel and fragrance	Irritant reaction to surgical tapes on upper arm after 48 hours	+/-	Irritant reaction to surgical tapes on upper arm after 48 hours Assessment by clinician	+/-	None	N/A
	Josefson 2011	Allergic reaction to nickel	Medical plaster patches (Nixema) on upper arm with	+/-	Medical plaster patches (Finn	+/-	Same day	N/A

			readings on days 3-4		chambers® on Scanpor® tape or IQ Ultra® Chambers) on back with readings on days 3-4 and/or day 7 Assessment by dermatologist			
HIV	Assiimwe 2014	Antibodies for HIV1 and HIV2	Oral fluid test using OraQuick® In-Home Rapid HIV-1/2 Antibody Test (Orasure Technologies)	+/-	Finger pick blood test. Nationally approved algorithm of POC rapid HIV tests (Determine (Abbot Laboratories), STAT-PAK (Chembio Diagnostic Systems Inc) and Unigold (Trinity Biotech plc) as a tiebreaker). Assessment by research assistants.	+/-	12 -72 hours	N/A
	Belete 2019	Antibodies for HIV1 and HIV2	Oral fluid test using OraQuick® Rapid HIV-1/2 Antibody Test (Orasure Technologies)	+/-	Finger pick blood test. Nationally approved algorithm of POC rapid HIV tests (Wanti (Beijing), Unigold (Trinity Biotech plc), Vikia). Assessment by health professional.	+/-	Same time	N/A
	Choko 2011	Antibodies for HIV1 and HIV2	Oral fluid test using OraQuick ADVANCE® Rapid HIV-1/2 Antibody Test	+/-	Finger pick blood test. Algorithm of POC rapid HIV tests (Determine (Abbot Laboratories), Unigold (Trinity Biotech plc) and SD Bioline HIV i/II (Standard Diagnostics, Inc.) as a tiebreaker). Assessment by counsellor.	+/-	Same time	N/A
	Choko 2015	Antibodies for HIV1 and HIV2	Oral fluid test using OraQuick ADVANCE® Rapid HIV-1/2 Antibody Test	+/-	Finger pick blood test. Two parallel POC rapid HIV tests (Determine (Abbot Laboratories), Unigold (Trinity Biotech plc)). Assessment by nurse.	+/-	Approx. 1 week	1 – 12, 13 – 24 months (max. 1 test in 12 months)

	Kapaku 2017	Antibodies for HIV1 and HIV2	Oral fluid test using OraQuick® rapid HIV-1/2 Antibody Test (Orasure Technologies)	+/-	Venous blood test (EDTA tube) Testing in certified central laboratory (Abbott Architect HIV1 Ag/Ab combo assay, positive results confirmed by BioRad GS HIV combo Ag/Ab assay)	+/-	Within 8 hours	Once in 12 month study period.
	Kurth 2016	Antibodies for HIV1 and HIV2	Oral fluid test using OraQuick ADVANCE® Rapid HIV-1/2 Antibody Test	+/-	Venous blood test. ELISA testing by single person in certified central laboratory (Vironostika HIV Uni-Form II Ag/Ab (bioMe´rieux Inc.))	+/-	Within 8 hours	N/A
	Martinez Perez 2016	Antibodies for HIV1 and HIV2	Oral fluid test using OraQuick ADVANCE® Rapid HIV-1/2 Antibody Test	+/-	Finger pick blood test. POC rapid HIV tests (Determine (Abbot Laboratories), confirmatory Unigold (Trinity Biotech plc)). Assessment by HIV counsellor.	+/-	Same time	N/A
	Orasure 2012	Antibodies for HIV1 and HIV2	Oral fluid test using OraQuick® In-Home Rapid HIV-1/2 Antibody Test (Orasure Technologies)	+/-	Venous blood test. FDA approved serum EIA and Western blot in FDA approved laboratory.	+/-	Unclear	N/A
	Pant Pai 2013	Antibodies for HIV1 and HIV2	Oral fluid test using OraQuick® rapid HIV-1/2 Antibody Test (Orasure Technologies)	+/-	Venous blood test. ELISA with p24 Antigen tests in reference laboratories all within 24 hours (Architect HIV Ag/Ab combo (Abbott Laboratories), positive results confirmed by Western Blot)	+/-	Same time	N/A

HIV - Human immunodeficiency virus, POC – Point of care, max. – maximum, FDA – Food and Drug Administration, USA, N/A – not applicable; NR – not reported; KOH – potassium hydroxide; PCR – polymerase chain reaction; * whilst no reported level was given for VI-SENSE, it was reported that the polymer used in this product had a range of 4.3 – 5.1 pH
