# Supplementary material for: Accuracy of self-diagnosis in conditions commonly managed in primary care: diagnostic accuracy systematic review and meta-analysis
Source: BMJ Open. 2023 Jan 10;13(1):e065748. doi: 10.1136/bmjopen-2022-065748 (PMC9835960; doi:10.1136/bmjopen-2022-065748)
Supplement: Author's manuscript [file bmjopen-2022-065748.draft_revisions.pdf]

BMJ Open is committed to open peer review. As part of this commitment we make the peer review history of every article we publish publicly available.

When an article is published we post the peer reviewers' comments and the authors' responses online. We also post the versions of the paper that were used during peer review. These are the versions that the peer review comments apply to.

The versions of the paper that follow are the versions that were submitted during the peer review process. They are not the versions of record or the final published versions. They should not be cited or distributed as the published version of this manuscript.

BMJ Open is an open access journal and the full, final, typeset and author-corrected version of record of the manuscript is available on our site with no access controls, subscription charges or pay-per-view fees (http://bmjopen.bmj.com).

If you have any questions on BMJ Open's open peer review process please email info.bmjopen@bmj.com

BMJ Open

Accuracy of self-diagnosis in conditions commonly managed in primary care: Diagnostic accuracy systematic review and meta-analysis

Journal:	*BMJ Open*
Manuscript ID	bmjopen-2022-065748
Article Type:	Original research
Date Submitted by the Author:	15-Jun-2022
Complete List of Authors:	Mclellan, Julie; University of Oxford Nuffield Department of Primary Care Health Sciences, Nuffield Department of Primary Health Care Sciences<br>Heneghan, Carl; University of Oxford Nuffield Department of Primary Care Health Sciences, Primary Health Care<br>Roberts, Nia; University of Oxford Bodleian Libraries, Bodleian Health Care Libraries,<br>Pluddemann, Annette; University of Oxford Nuffield Department of Primary Care Health Sciences, Nuffield Department of Primary Care Health Sciences
Keywords:	Adult dermatology < DERMATOLOGY, HIV & AIDS < INFECTIOUS DISEASES, PRIMARY CARE, GYNAECOLOGY

SCHOLARONE™
Manuscripts

I, the Submitting Author has the right to grant and does grant on behalf of all authors of the Work (as defined in the below author licence), an exclusive licence and/or a non-exclusive licence for contributions from authors who are: i) UK Crown employees; ii) where BMJ has agreed a CC-BY licence shall apply, and/or iii) in accordance with the terms applicable for US Federal Government officers or employees acting as part of their official duties; on a worldwide, perpetual, irrevocable, royalty-free basis to BMJ Publishing Group Ltd ("BMJ") its licensees and where the relevant Journal is co-owned by BMJ to the co-owners of the Journal, to publish the Work in this journal and any other BMJ products and to exploit all rights, as set out in our licence.

The Submitting Author accepts and understands that any supply made under these terms is made by BMJ to the Submitting Author unless you are acting as an employee on behalf of your employer or a postgraduate student of an affiliated institution which is paying any applicable article publishing charge ("APC") for Open Access articles. Where the Submitting Author wishes to make the Work available on an Open Access basis (and intends to pay the relevant APC), the terms of reuse of such Open Access shall be governed by a Creative Commons licence – details of these licences and which Creative Commons licence will apply to this Work are set out in our licence referred to above.

Other than as permitted in any relevant BMJ Author's Self Archiving Policies, I confirm this Work has not been accepted for publication elsewhere, is not being considered for publication elsewhere and does not duplicate material already published. I confirm all authors consent to publication of this Work and authorise the granting of this licence.

For peer review only

Accuracy of self-diagnosis in conditions commonly managed in primary care: Diagnostic accuracy systematic review and meta-analysis

Julie McLellan[1], Carl Heneghan[1], Nia Roberts[2], Annette Plüddemann[1]

[1] Nuffield Department of Primary Care Health Sciences, University of Oxford, OX2 6GG

[2] Bodleian Health Care Libraries, University of Oxford, Oxford, UK

Corresponding author: Annette Plüddemann, Nuffield Department of Primary Care Health Sciences, University of Oxford, OX2 6GG, annette.pluddemann@phc.ox.ac.uk, +44 (0)1865 289353

Keywords: Diagnostic accuracy systematic review, meta-analysis, self-diagnosis, common conditions in primary care,

Abstract word count: 299

Manuscript word count: 4073

For peer review only

Abstract

Objectives:

To assess the diagnostic accuracy of self-diagnosis compared to a clinical diagnosis for common conditions in primary care

Design:

Systematic review and meta-analysis

Data sources:

Medline, Embase, Cochrane Central Register of Controlled Trials(CENTRAL), Cochrane Database of Systematic Reviews(CDSR), and CINAHL from inception to 25 January 2021

Study selection:

Eligible studies were prospective or retrospective studies comparing the results of self-diagnosis of common conditions in primary care to a relevant clinical diagnosis or laboratory reference standard test performed by a healthcare service provider. Studies that considered self-testing only were excluded.

Data extraction:

Two authors independently extracted data using a predefined data extraction form and assessed risk of bias using QUADAS-2.

Methods & Results:

Searches identified 5,047 records resulting in 18 included studies. Five studies reported on the accuracy of self-diagnosis of vaginal infection, four for common skin conditions, and nine for the self-diagnosis for HIV. For self-diagnosis of vaginal infection and common skin conditions meta-analysis was not appropriate and data were reported narratively. Nine studies, using point-of-care oral fluid tests, reported on the accuracy of self-diagnosis of HIV and data were pooled using bivariate meta-analysis methods. For these nine studies the pooled sensitivity was 92.8% (95%CI, 86% to 96.5%) and specificity was 99.8% (95%CI, 99.1% to 99.9%). Post hoc, the robustness of the pooled findings was tested in a sensitivity analysis only including four studies using laboratory testing as the reference standard. The pooled sensitivity reduced to 87.7% (95%CI 81.4% to 92.2%) and the specificity remained the same. The quality of all 18 included studies was assessed as mixed and overall study methodology was not always well described.

Conclusions and implications of key findings:

Overall, there was a paucity of evidence. The current evidence does not support routine self-diagnosis for common conditions in primary care.

PROSPERO registration number: CRD42018110288

For peer review only

Article Summary

Strengths and limitations of this study

- This systematic review summarises and interprets the available evidence on self-diagnosis of conditions managed in primary care
- This search strategy was extensive including publications identified from databases Medline, Embase, Cochrane Central Register of Controlled Trials (CENTRAL), Cochrane Database of Systematic Reviews (CDSR), and CINAHL, up to January 2021
- Standard methodology for systematic review of diagnostic accuracy studies was used, including study quality appraisal using QUADAS-2
- There was a paucity of evidence for many common conditions
- Lack of evidence meant meta-analysis was possible only for one condition

For peer review only

Introduction

The workload in primary care continues to increase (1, 2), in part due to global population increases, but also due to age of populations, change in lifestyle and more complex health problems (3). Increased workload has been recognised in the UK as an important factor in working conditions for primary care physicians (General Practitioners, GPs) and strategies are required to manage the workload (4).

One strategy is the potential for self-diagnosis and self-treatment by patients of some commonly occurring conditions. If feasible, this could lead to more rapid diagnosis and treatment reducing the burden on primary care services. The prospect of self-diagnosis is controversial with concerns if results are misinterpreted or patients fail to confirm their findings to a physician (5). In terms of the evidence, the first important question is to assess the diagnostic accuracy of self-diagnosis in the primary care setting. Subsequently, in order to support self-diagnosis, the efficacy needs to be assessed, to inform which conditions can self-diagnosed safely, in which circumstances, and by whom.

Cooke and colleagues recently reported the 30 most commonly managed conditions in primary care in Australia, which has a health landscape broadly comparable with western Europe (6). This list arises from survey data collected between January 2009 and December 2010, which included 194,100 patient encounters from 1,941 GPs.

This systematic review, therefore, aimed to assess the diagnostic accuracy of self-diagnosis compared to a clinical diagnosis for common conditions in primary care by a healthcare provider.

Methods

Types of studies

We included prospective or retrospective studies comparing the results of self-diagnosis of common conditions in primary care to the results of a relevant clinical diagnosis or laboratory reference standard test. We excluded studies with a case-control design due to their high risk of bias (4).

Population: Adults self-diagnosing common conditions in primary care. Common conditions included were broadly based on those reported by Cooke et al (6) and relevant for self-diagnosis (see online supplementary table 1). Studies in children, based in animals or non-human samples were excluded.

Index test: Self-diagnosis, where we defined "self-diagnosis" as a diagnosis made by the patients in the study, including self-evaluation and interpretation of results of rapid tests. Studies that considered self-testing only and not as part of self-diagnosis were excluded, in addition studies assessing accuracy of self-monitoring of an existing condition were excluded.

Reference standard: Clinical diagnosis or laboratory test performed by a healthcare service provider. We excluded studies comparing self-diagnosis with diagnosis by allied health professionals or pharmacists.

For peer review only

Outcome measures: Reported diagnostic accuracy measures (e.g. sensitivity, specificity, likelihood ratios, predictive values, etc.) and primary data for 2x2 tables. We excluded studies reporting only measures of agreement.

Search methods to identify studies

The search strategy was based on a combination of terms for self-testing and self-diagnosis, diagnostic accuracy terms (e.g. sensitivity, specificity, etc.) and terms for common conditions in primary care (6) (see online supplementary table 2 for full search strategy).

We searched the following electronic databases from inception to 25 January 2021: Medline (OvidSP)[1946-present], EMBASE (OvidSP)[1974-present], Cochrane Central Register of Controlled Trials (CENTRAL) and Cochrane Database of Systematic Reviews (CDSR) via Cochrane Library, Wiley)[Issue 1 of 12, January 2021] and CINAHL (EBSCOHost)[1982-present]. No restrictions were imposed on study population numbers or language (studies in languages other than English were translated). Letters, narrative reviews, and other non-primary sources were excluded. The reference lists of included studies, plus the first five "similar articles" identified through PubMed for these studies, and reference lists of relevant systematic reviews were used to identify further relevant publications. References were imported into Endnote X9 (7) where duplicates were removed.

Data collection and analysis

Selection of studies

Two reviewers independently applied the selection criteria to the titles and abstracts of the study reports identified by the searches. Full text of all studies that met the inclusion criteria were reviewed to agree the final list of included studies. Disagreements between reviewers were resolved by discussion and where agreement could not be reached a third reviewer was consulted.

Data extraction and management

Two reviewers (JM, AP) independently extracted information from selected studies into a pre-defined data extraction sheet (see online supplementary table 3) and crosschecked the data. Disagreements were resolved by discussion.

Assessment of methodological quality

We used the Quality Assessment of Diagnostic Accuracy Studies-2 (QUADAS-2) (8) tool to assess methodological quality of included studies, considering risk of bias in four domains (patient selection, index test, reference standard, flow and timing), as well as assessing the applicability of the studies to the review research question. Two reviewers (JM, AP) independently assessed studies' methodological quality; disagreements were resolved by discussion, or if necessary, by a third reviewer.

Statistical analysis and data synthesis

Data were presented and analysed based on the condition being diagnosed. We compiled summary tables outlining the detailed study information of included studies, including the patient sample, condition, study design, setting, the test under evaluation, the comparator, and conduct of the study. We extracted binary diagnostic accuracy data from all studies and constructed 2×2 tables.

For peer review only

Meta-analysis

We used Review Manager (9) to produce paired forest plots to explore the between-study variability of sensitivity and specificity across the included studies. For each study estimate of sensitivity and specificity, corresponding 95% confidence intervals were shown to illustrate the uncertainty related to each study estimate. Where different thresholds were applied these were reported. Where appropriate, we used bivariate meta-analysis methods (10) to generate pooled estimates of sensitivity and specificity. Due to the nature of the data a change was made to the protocol and RStudio (11) was used to generate the model parameters to input into Revman (9).

Investigating heterogeneity

For medical conditions for which data from more than one study was available, and where it was possible to investigate between-study heterogeneity in the results, inclusion of study level characteristics as covariates in meta-analysis and subgroup analyses were considered. These approaches were carried out if there was sufficient data available and sub-group specific pooled estimates were thought to be of clinical relevance.

Investigating reporting bias

Funnel plots used to detect publication bias in reviews of RCTs have been shown to be misleading for diagnostic test accuracy reviews (12, 13). Funnel plots as an assessment of reporting bias were therefore not be included in this review.

Patient involvement

Members of the public were part of the Research Programme Committee of the National Institute for Health Research (NIHR) programme grant that funded this study. Updates and details about the study were presented to the committee while the study was ongoing, and the public members provided feedback. This review formed part of the NIHR Evidence Synthesis Working Group (ESWG) and members of the public who were part of the ESWG steering committee commented on the protocol for the study and on updates presented to the steering committee.

The full protocol is provided as online supplementary table 4 and is registered on PROSPERO reference number CRD42018110288.

Results

Figure 1 shows a summary of the search results and the inclusion and exclusion of studies. After removal of duplicates, 5,047 records were identified through database searches, websites and citation searching. This resulted in full texts of 170 articles being assessed for eligibility, and 20 included articles (14-33) reporting results of 18 individual studies (14-20, 22-28, 30-33). These 18 included studies fell into 3 broad groups of commonly managed conditions: Female genital check-up, dermatitis, contact/allergic and viral disease, not otherwise specified. No studies of self-diagnosis were found for any other conditions in primary care.

Most excluded studies only reported on patients' ability to self-test (or self-monitor an existing condition) with the diagnosis being made by a clinician, and did not report diagnostic accuracy of self-diagnosis.

For peer review only

Of the included studies, five reported on the accuracy of self-diagnosis of vaginal infection (11-15), four for common skin conditions (19, 20, 22, 23) and nine for the self-diagnosis for HIV (24-28, 30-33). Online supplementary tables 5 and 6 summarise the characteristics of included studies and characteristics of self-diagnostic (index) and reference tests, respectively. Paired plots of sensitivity and specificity were generated, grouping the studies by the condition to be diagnosed (Figures 2-4). For studies examining the accuracy of self-diagnosis of vaginal infection and in common skin conditions (14-20, 22, 23), meta-analysis was not appropriate due to the between-study heterogeneity and the overall low number of studies, which would make meta-analysis uninformative (34).

Self-diagnosis of vaginal infections

Five studies assessed the accuracy of self-diagnosis of Bacterial vaginosis and/or infection with *Trichomonas vaginalis (14-18),* with one study assessing the self-diagnosis of *Candida* vaginitis (17) (Figure 2). For Bacterial vaginosis, the accuracy of a vaginal fluid test using a pH strip (for Bacterial vaginosis) was assessed with laboratory testing (Gram staining) as the reference standard in two studies (14, 15) (online supplementary table 5). For the diagnosis of Bacterial vaginosis and/or *T. vaginalis*, a panty liner test kit (VI-SENSE) for vaginal discharge was assessed against a combination of clinical and laboratory assessment as the reference test in one study (16); and a vaginal fluid self-diagnosis kit for women in the military was assessed with clinical and laboratory assessment as the reference test in the second study (17). One study used a vaginal fluid dipstick test for the presence of *T. vaginalis* (OSOM *Trichomonas* rapid test) compared to a laboratory PCR as a reference test (18). For the self-diagnosis of *Candida* vaginosis a military self-testing kit based on a combination of the measurement of pH, amines and the symptom of vaginal itching for self-diagnosis was compared to a combination of clinical and microbiological laboratory assessment in one study (17).

Bacterial vaginosis; Bacterial vaginosis and/or Trichomonas vaginalis

The sensitivity of a self-taken swab applied to a pH test strip for self-diagnosis of Bacterial vaginosis ranged from 0.45 (95%CI, 0.34-0.56) (14) to 0.60 (95%CI, 0.55-0.66) (17) at a pH cut-off of $\geq$ 4.7. Notably, Ryan-Wenger et al (17), the study reporting a sensitivity of 0.60, also included symptoms (vaginal itching) and the presence of amines as part of the assessment, which may explain the higher sensitivity. Donders et al (14) assessed a lower cut-off of pH $\geq$ 4.5 and showed an increased sensitivity of 0.95 (95%CI, 0.88-0.99); however at the expense of specificity. The study on pregnant women (15) assessing the accuracy of pH test strips did not specify the pH cut-off and reported a sensitivity of 0.36 (95%CI, 0.17-0.59) with a specificity of 0.88 (95%CI, 0.82-0.93). The specificity for the pH test strip tests ranged from 0.5 (95%CI, 0.43-0.56) (17) to 0.81 (95%CI, 0.75-0.85) (14) at the pH cut-off of $\geq$ 4.7, decreasing to 0.41 (95%CI, 0.34-0.47) at the lower cut-off of pH $\geq$ 4.5 (14). Interestingly the low specificity of 0.5 was reported by the study combining the pH test strip with symptoms and the presence of amines (17).

The study assessing the vaginal discharge test using a panty liner test kit with an indicator strip incorporated in the liner (16) reported a sensitivity of 0.91 (95%CI, 0.86-0.94) and specificity of 0.81 (95%CI, 0.76-0.86) for the diagnosis of Bacterial vaginosis and/or *T. vaginalis* infection.

Trichomonas vaginalis

For peer review only

One study in Brazil assessed a rapid immunochromatographic *T. vaginalis* test for use at home (18) and reported a sensitivity of 0.68 (95%CI, 0.43-0.87) and specificity of 1.00 (95%CI, 0.99-1.00) for self-diagnosis of *T. vaginalis* infection.

Candida vaginitis

Only one study (17) specifically assessed the diagnostic accuracy of self-diagnosis of *Candida* vaginitis, which formed part of the military self-testing kit, and reported a sensitivity of 0.18 (95%CI, 0.12-0.25) and specificity of 0.89 (95%CI, 0.85-0.92).

Self-diagnosis of common skin conditions

Four studies assessed the accuracy of self-diagnosis of common skin conditions (19, 20, 22, 23) (Figure 3). We included 2 studies that were outside our age inclusion criteria: Berghoi 2011 reported patients included had a mean age 17.5 years old, nevertheless these patients would have been 16+ years to qualify as apprentice hairdressers. And Svensson 2002 reported the mean age of patients as 40.4 years (no standard deviation), but included patients from age 16.

Eczema

Two studies assessed the accuracy of self-diagnosis based on a self-evaluated questionnaire of signs and symptoms for the diagnosis of eczema alongside a self-assessment of the presence or absence of eczema based on the questionnaire results, compared to assessment by a clinician (19, 20). Overall, 710 participants were included across the two studies. The reported sensitivity ranged from 0.7 (95%CI, 0.53- 0.84) to 0.87 (95%CI, 0.78-0.93) and specificity ranged from 0.79 (95%CI, 0.70-0.86) to 1.00 (95%CI, 0.99-1.00). The relatively high specificity suggests the potential for patients to use the questionnaire as a tool to confirm that they have eczema and therefore seek healthcare advice; however, it should be noted that there was an unclear risk of bias regarding the patient selection. In Svensson et al (20), the patients were recruited at a dermatology outpatient clinic, where they had been referred to and 113 patients in the study reported having had a diagnosis of eczema in the last 12 months, suggesting a more selected population with a higher pre-test probability. Whilst the setting in the study by Bregnhoj et al. (19) was not reported, it was conducted amongst hairdressers who may have had more experience of eczema either themselves or colleagues being diagnosed with the condition, given the nature of their profession. They may be more aware of the signs and symptoms and may constitute a selected population. Therefore, the diagnostic accuracy of the questionnaire for self-diagnosis may be dependent on the type of population.

Skin allergy

Two studies assessed the diagnostic accuracy of self-diagnosis to detect an allergic skin reaction including 408 participants across the two studies; one study assessed nickel and/or fragrance allergy (22), and the other nickel allergy alone (23). Both studies used a patch test applied to the arm, which as self-evaluated by participants 2-4 days later. Dermatologists then also evaluated the patch tests as the reference standard. One study recruited participants at hospital dermatology departments (23), while the other recruited through a newspaper advertisement targeted at people with a self-suspected allergy towards fragrance and/or nickel (22). Sensitivity ranged from 0.72 (95%CI, 0.57-0.84) to 0.97 (95%CI. 0.87-1.00) and specificity ranged from 0.87 (95%CI, 0.80-0.92) to 0.91 (95%CI, 0.85-0.95). Elsner et al.

For peer review only

(22) also reported that participants found the information regarding how to apply the test extensive and detailed, the information regarding self-evaluation of the test was limited and should be improved.

Self-diagnosis of HIV

Nine studies were identified that reported the diagnostic accuracy of self-testing and self-diagnosis of HIV (24-28, 30-33). In all studies self-diagnosis was undertaken unsupervised using a rapid point of care (POC) oral fluid test manufactured by OraSure technologies, either OraQuick Inhome intended for lay users or OraQuick Advance intended for professional use. The studies recruited 13103 participants, and all were conducted in African countries except for the phase III trial in the USA by OraSure Technologies (33). The 2019 global HIV prevalence rates for women and men aged 15 to 49 were 0.8% (95%CI, 0.7–1.0) and 0.6% (95%CI, 0.5-0.8) respectively with the overall highest prevalence by country in Eswatini (Africa) at 27.1% (95%CI, 25.4-28.8] (35). All included studies had prevalence rates above the global averages for men and women or, if not reported, were in countries with high prevalence rates. Prevalence rates ranged from 2.12% in the USA study (33) to 22.1% in the Ugandan study (24). The USA study was conducted in 20 clinical sites, 17 identified as high prevalence sites (2.6%) and 3 as low prevalence sites (0.1%). All studies enrolled participants from the general population including the USA where no breakdown of sexual orientation was reported. The reported sensitivity and specificity were similar between studies (Figure 4a); the single study (32) reporting a lower estimate for sensitivity still had a confidence interval that overlapped with half of the other studies. The pooled sensitivity based on all 9 included studies was 92.8% (95%CI, 86% to 96.5%) and the pooled specificity was 99.8% (95%CI, 99.1% to 99.9%). The studies showed low heterogeneity (Figure 4b). The reference standard used in the studies was one of three types: four studies took a venous sample which was sent to a laboratory for testing (28, 30, 32, 33), two studies used a nationally approved algorithm based on a combination of rapid POC tests (24, 25) and three studies used a study based algorithm again based on rapid POC tests (26, 27, 31). In three studies using POC tests for the reference standard, the diagnosis may have been by clinicians, but it was unclear. These studies reported the diagnosis by a research assistant (24) or a counsellor (26, 31). Post hoc, a sensitivity analysis was conducted to test the robustness of the pooled findings by removing the studies using POC tests as the reference standard (including tests where it was unclear whether diagnosis was by a clinician) and only including those studies using laboratory testing. Based on four studies using laboratory testing as the reference standard (28, 30, 32, 33), the pooled sensitivity was 87.7% (95%CI 81.4% to 92.2%) and the pooled specificity was 99.8% (95%CI 98.9% to 99.9%). No data were reported by participant characteristics such as gender, ethnicity, or sexual orientation.

A number of studies (24-26, 30-32) reported on the viability or feasibility of the oral fluid self-test stating that participants found it easy to conduct, but acknowledged that instructions should be adapted to the population using the test, particularly the literacy levels. Furthermore, users must be encouraged to receive a confirmatory test.

Methodological quality of included studies

Assessment of the quality of included studies using the QUADAS-2 framework (8) is presented in Figure 5, which summarises the overall risk of bias and applicability concerns.

For peer review only

For patient selection, the risk of bias overall was mixed; where studies were rated unclear in this domain it was because several studies either recruited a selected population with a potentially higher pre-test probability or they did not clearly report the recruitment strategy and whether eligible patients were consecutively recruited. However, with the exception of one study, applicability concerns were low. In several cases, although the population may have been a selected population (e.g. at high risk of HIV infection or skin eczema; or with a prior history of eczema or vaginal infection), it could be argued that these might be the populations where self-diagnosis and/or self-testing may be most relevant. Overall risk of bias regarding the conduct of the index test was low and in most studies participants were blinded to the results of the reference test, though in some studies this was not clearly described. Risk of bias with respect to the reference test was unclear or high in some studies as assessors were either not blinded to the results of the index test or blinding was unclear. For the domain of flow and timing, several studies were judged to be at high risk of bias as the interval between testing was frequently not explicitly reported. In addition differential reference bias was identified as present in some studies and unclear in others, in particular it was unclear in several studies how clinical assessment was conducted or standardised. Overall study procedures were not always clearly described.

Discussion

With the increasing workload in primary care (1) and the continued development of rapid tests, including those that are intended to be used by patients, we aimed to assess the evidence for the diagnostic accuracy of self-diagnosis. We identified limited evidence on the diagnostic accuracy of self-diagnosis: only 20 publications (reporting data from 18 studies) specifically assessed the accuracy of self-diagnosis, covering three commonly managed conditions, namely vaginal infections, common skin conditions and HIV. Interestingly, no studies of self-diagnosis were found for any other conditions in primary care. It was particularly notable that we did not find any studies assessing the diagnostic accuracy of self-diagnosis of common primary conditions such as upper respiratory tract and urinary tract infections.

The evidence for self-diagnosis of vaginal infection suggests a lack of sufficient accuracy to aid self-diagnosis. Tests relying on self-swabs and pH strips showed low sensitivity (below 60%), and therefore would not be useful to rule out disease. Although sensitivity improved to 95% with increasing pH cut-off, this occurred at the cost of specificity, which dropped from 81% to 41%. Therefore, using the test at this cut-off would result in concern for missed diagnoses. The immunochromatography test also showed insufficient sensitivity (68%) to be of use as a rule-out test. The panty liner test, however, had high sensitivity of 91% and may prove to be a useful rule-out test, although this result is limited as it is based on one study judged to be at unclear risk of bias, with a high risk of bias in the flow and timing domain. In terms of their use to confirm the presence of vaginal infection, the highest specificity (100%) was reported for the immunochromatographic test. This may be a useful test to aid self-diagnosis of vaginal infection in systems that currently rely on syndromic management, such as low resource settings, particularly to improve the targeting of antimicrobial prescribing. However, it should be noted this result is also based on one study and requires confirming in a larger study.

The allergy patch tests overall showed reasonable accuracy (sensitivity 72-97%; specificity 87-91%) and may be useful as an initial self-screening test for patients with a suspected

For peer review only

nickel or fragrance allergy; however it could be argued that the main use of these tests might be to safely rule out a contact allergy. The reported diagnostic accuracy suggests the tests are not sufficiently sensitive as a rule-out test, particularly given the relatively wide confidence intervals. The tests may however be useful in settings where access to dermatology services is scarce. The self-diagnosis of eczema using a questionnaire of signs and symptoms showed specificity ranging from 0.79 to 1.00, suggesting this test might be useful as a confirmation test for patients for suspect they have eczema and to then seek treatment and management advice. The selected nature of the patients in the studies (i.e. patients with a previous eczema diagnosis and hairdressers, who may encounter eczema more frequently due to their profession) may overestimate the accuracy of the test; however, it could be argued that these might be the populations in whom the test is most relevant.

We identified nine studies that reported the accuracy of self-diagnosis of HIV (sensitivity 93%, specificity 99%). However, the sensitivity is reduced to 88% when only studies using a venous sample and laboratory testing as the reference standard are included. With a sensitivity of 88%, the accuracy data would not support the use of this test as a rule-out test, particularly given the clinical consequences of a false negative test result. Evidence suggests there may be benefits to self-initiated HIV testing, including early identification, increased likelihood for the uptake of HIV prevention interventions, and a reduction in sexual risk behaviours (36), warranting further research, in particular in resource-limited settings where access to testing sites may be a barrier. However, HIV self-testing should also be considered in the context of linkage to care, access to counselling, and adequate regulatory and quality assurance systems (37).

The search strategy for this review was broad and extensive with few restrictions resulting in the high number of publications to screen. Whilst it is possible, it is unlikely studies were missed. We are unaware of any other reviews examining this research question. The main limitation was the lack of available evidence for a number of common conditions; studies reporting on self-testing alone were more common, but few studies assessed self-diagnosis, with patients interpreting the test results and making a diagnosis independently. For the three conditions where we identified studies reporting on the diagnostic accuracy of self-diagnosis, there was a paucity of evidence. Many studies were not replicated and included small sample sizes and contained methodological biases that limited the application of the results to practice. For self-diagnosis of vaginal infections, common skin conditions, and HIV further research is required to draw a definitive conclusion on the efficacy of self-diagnosis. For other common conditions in primary care, research is needed on self-diagnosis where this option is available, and studies should go beyond considering self-testing alone and assess the diagnostic accuracy of self-diagnosis. Terminology for self-diagnosis, self-testing and self-screening is overlapping in some cases and needs clarifying. Finally, research is required into the patient's readiness and attitude towards self-diagnosis along with its effect on the patient/physician relationship.

The current limited evidence does not support routine self-diagnosis for vaginal infections, common skin conditions and HIV in primary care.

Acknowledgements

The authors would like to thank Hayley Jones for joint authorship of the original protocol, Elizabeth Spencer for assistance with preparation of the protocol, and Emily McFadden for assistance with screening of studies for inclusion.

For peer review only - http://bmjopen.bmj.com/site/about/guidelines.xhtml

Contributors

AP and CH conceived the idea for this review. AP led the research, developed the protocol, and conducted the review (screening and extraction). CH developed the protocol and provided clinical input. NR devised and conducted the search strategy, and approved the manuscript. JM conducted the review (screening, extraction, data analysis). JM, AP and CH jointly prepared the manuscript.

Funding

This project was funded by National Institute for Health Research School for Primary Care Research (NIHR SPCR) [Project Number 390]

Data availability statement

All data are available in published articles.

Competing interests

Julie McLellan reports grants from NIHR School of Primary Care Research (Evidence Synthesis Working Group Project Number 390) and part-funding from the Thames Valley Applied Research Collaborative (ARC), during the conduct of the study; and occasionally receives expenses for teaching Evidence-Based Medicine

Dr. Plüddemann reports grants from NIHR School of Primary Care Research (Evidence Synthesis Working Group Project Number 390), during the conduct of the study; and occasionally receives expenses for teaching Evidence-Based Medicine.

Dr. Heneghan reports receiving expenses and fees for his media work. He has received expenses from the WHO and holds grant funding from the NIHR, the NIHR School of Primary Care Research, The Wellcome Trust and the WHO. He has received financial remuneration from an asbestos case. He has also received income from the publication of a series of toolkit books published by Blackwells. On occasion, he receives expenses for teaching EBM and is also paid for his GP work in NHS out of hours. CEBM jointly runs the EvidenceLive Conference with the BMJ and the Overdiagnosis Conference with some international partners which are based on a non-profit making model.

Ethics Approval

Not applicable.

References

1. Hobbs FDR, Bankhead C, Mukhtar T, Stevens S, Perera-Salazar R, Holt T, et al. Clinical workload in UK primary care: a retrospective analysis of 100 million consultations in England, 2007-14. Lancet. 2016;387(10035):2323-30.
2. Irving G, Neves AL, Dambha-Miller H, Oishi A, Tagashira H, Verho A, et al. International variations in primary care physician consultation time: a systematic review of 67 countries. BMJ Open. 2017;7(10):e017902.
3. Orgnaistion WH. Global status report on noncommunicable diseases 2014. 2014.

For peer review only

4. British Medical Association. BMA guidance: Controlling workload in general practice strategy 2020 [Available from: https://www.bma.org.uk/advice-and-support/gp-practices/managing-workload/controlling-workload-in-general-practice-strategy] Accessed 18 June 2021.
5. Hynes V. The trend toward self-diagnosis. CMAJ. 2013;185(3):E149-E50.
6. Cooke G, Valenti L, Glasziou P, Britt H. Common general practice presentations and publication frequency. Australian Family Physician. 2013;42:65-8.
7. The EndNote Team. Endnote. Endnote X9 ed. Philadelphia, PA: Clarivate; 2013.
8. Whiting PF, Rutjes AW, Westwood ME, Mallett S, Deeks JJ, Reitsma JB, et al. QUADAS-2: a revised tool for the quality assessment of diagnostic accuracy studies. Ann Intern Med. 2011;155(8):529-36.
9. Review Manager (RevMan) [Computer program]. Version 5.4. The Cochrane Collaboration. 2020.
10. Reitsma JB, Glas AS, Rutjes AW, Scholten RJ, Bossuyt PM, Zwinderman AH. Bivariate analysis of sensitivity and specificity produces informative summary measures in diagnostic reviews. J Clin Epidemiol. 2005;58(10):982-90.
11. RStudio Team. 2021. RStudio: Integrated Development Environment for R. RStudio, PBC, Boston, MA [Available from: http://www.rstudio.com/] Accesssed 22 June 2021.
12. Deeks JJ, Macaskill P, Irwig L. The performance of tests of publication bias and other sample size effects in systematic reviews of diagnostic test accuracy was assessed. J Clin Epidemiol. 2005;58(9):882-93.
13. Leeflang MM, Deeks JJ, Gatsonis C, Bossuyt PM. Systematic reviews of diagnostic test accuracy. Ann Intern Med. 2008;149(12):889-97.
14. Donders GGG, Gonzaga A, Marconi C, Donders F, Michiels T, Eggermont N, et al. Increased vaginal pH in Ugandan women: what does it indicate? European Journal of Clinical Microbiology & Infectious Diseases. 2016;35(8):1297-303.
15. Sungkar A, Purwosunu Y, Aziz MF, Pratomo H, Sutrisna B, Sekizawa A. Influence of early self-diagnosis and treatment of bacterial vaginosis on preterm birth rate. International Journal of Gynaecology & Obstetrics. 2012;117(3):264-7.
16. Geva A, Bornstein J, Dan M, Shoham HK, Sobel JD. The VI-SENSE-vaginal discharge self-test to facilitate management of vaginal symptoms. American Journal of Obstetrics & Gynecology. 2006;195(5):1351-6.
17. Ryan-Wenger NA, Neal JL, Jones AS, Lowe NK. Accuracy of vaginal symptom self-diagnosis algorithms for deployed military women. Nursing Research. 2010;59(1):2-10.
18. Jones HE, Lippman SA, Caiaffa-Filho HH, Young T, van de Wijgert JHHM. Performance of a rapid self-test for detection of Trichomonas vaginalis in South Africa and Brazil. Journal of Clinical Microbiology. 2013;51(3):1037-9.
19. Bregnhoj A, Sosted H, Menne T, Johansen JD. Validation of self-reporting of hand eczema among Danish hairdressing apprentices. Contact Dermatitis. 2011;65(3):146-50.
20. Svensson A, Lindberg M, Meding B, Sundberg K, Stenberg B. Self-reported hand eczema: symptom-based reports do not increase the validity of diagnosis. British Journal of Dermatology. 2002;147(2):281-4.
21. Elsner P, Bjarnoe H, Aberer W, Brasch J. Evaluation of a self-test device in allergic contact dermatitis. Dermatitis. 2009;20 (4):230-1.
22. Elsner P, Bjarnøe H, Aberer W, Brasch J. Evaluation of a Self-Test Device Used in Allergic Contact Dermatitis. Skin Pharmacol Physiol. 2015;28(5):257-63.
23. Josefson A, Svensson A, Farm G, Engfeldt M, Meding B. Validation of self-testing as a method to estimate the prevalence of nickel allergy. Acta Dermato-Venereologica. 2011;91(5):526-30.

For peer review only

24. Asiimwe S, Oloya J, Song X, Whalen CC. Accuracy of un-supervised versus provider-supervised self-administered HIV testing in Uganda: a randomized implementation trial. AIDS and behavior. 2014;18(12):2477-84.
25. Belete W, Deressa T, Feleke A, Menna T, Moshago T, Abdella S, et al. Evaluation of diagnostic performance of non-invasive HIV self-testing kit using oral fluid in Addis Ababa, Ethiopia: A facility-based cross-sectional study. PLoS One. 2019;14(1):e0210866.
26. Choko AT, Desmond N, Webb EL, Chavula K, Napierala-Mavedzenge S, Gaydos CA, et al. The uptake and accuracy of oral kits for HIV self-testing in high HIV prevalence setting: a cross-sectional feasibility study in Blantyre, Malawi. Plos medicine. 2011;8(10):e1001102.
27. Choko AT, MacPherson P, Webb EL, Willey BA, Feasy H, Sambakunsi R, et al. Uptake, Accuracy, Safety, and Linkage into Care over Two Years of Promoting Annual Self-Testing for HIV in Blantyre, Malawi: A Community-Based Prospective Study. PLoS Med. 2015;12(9):e1001873.
28. Kapaku K, Newman M, Maluzi K, Sigande L, Taegtmeyer M, Corbett E, et al., editors. Is OraQuick HIV-self-testing valid among intended users? Analysis from a clinical performance study in
Lusaka, Zambia. 22nd International AIDS Conference; 2017 24-26 July; Paris, France.
29. UNITAID/PSI HIV Self-Testing Africa (HIV STAR) study. A clinical performance study of self-testing using self-collected oral fluid transudate and the OraQuick® HIV Self-Test [Available from: https://hivstar.lshtm.ac.uk/files/2016/11/STAR-Protocol-for-Zambia-Clinical-Performance-Study-web.pdf] Accessed 18 June 2021.
30. Kurth AE, Cleland CM, Chhun N, Sidle JE, Were E, Naanyu V, et al. Accuracy and Acceptability of Oral Fluid HIV Self-Testing in a General Adult Population in Kenya. AIDS Behav. 2016;20(4):870-9.
31. Martínez Pérez G, Steele SJ, Govender I, Arellano G, Mkwamba A, Hadebe M, et al. Supervised oral HIV self-testing is accurate in rural KwaZulu-Natal, South Africa. Trop Med Int Health. 2016;21(6):759-67.
32. Pant Pai N, Behlim T, Abrahams L, Vadnais C, Shivkumar S, Pillay S, et al. Will an unsupervised self-testing strategy for HIV work in health care workers of South Africa? A cross sectional pilot feasibility study. PLoS One. 2013;8(11):e79772.
33. OraSure Technologies. Final Advisory Committee Briefing Materials: Available for Public Release. OraQuick In-Home HIV Test. Washington (D.C.): Food and Drug Administration: Blood Products Advisory Committee; 2012.
34. Macaskill P, Gatsonis C, Deeks JJ, Harbord RM, Takwoingi Y. Cochrane Handbook for Systematic Reviews of Diagnostic Test Accuracy.: The Cochrane Collaboration; http://srdta.cochrane.org; 2010.
35. UNAIDS. AIDSinfo Global factsheets 2019. HIV and AIDS Estimates [Available from http://aidsinfo.unaids.org/] Accessed 22 June 2021.
36. Adebayo OW, Salerno JP. Facilitators, Barriers, and Outcomes of Self-Initiated HIV Testing: An Integrative Literature Review. Res Theory Nurs Pract. 2019;33(3):275-91.
37. Njau B, Covin C, Lisasi E, Damian D, Mushi D, Boulle A, et al. A systematic review of qualitative evidence on factors enabling and deterring uptake of HIV self-testing in Africa. BMC Public Health. 2019;19(1):1289.

For peer review only

Figure 1: Study Selection

Figure 2: Paired forest plots of sensistivity and specificity for studies of self-diagnosis of vaginal infection (where Donders 2016a used pH threshold ≥ 4.5, Donders 2016b used pH threshold ≥ 4.7. Sungkar 2012 represents pooled data from 4 time points)

Figure 3: Paired forest plots of sensistivity and specificity for studies of self-diagnosis of common skin conditions (where Bregnhoj 2011 represents pooled data from recruitment and follow up time points)

Figure 4: Studies of self-diagnosis of HIV

(a) Paired forest plots of sensitivity and specificity (where Choko 2015 represents pooled data from 1- 12 and 13 – 24 months follow up time points)
(b) Receiver operating characteristic plot HIV self-diagnosis compared with clinical diagnosis or laboratory reference test grouped by reference test type (where size of symbol indicates study size)

Figure 5: QUADAS-2 summary of risk of bias and applicability concerns showing review authors' judgments about each domain for each included study (based on 19 studies (20 data sets))

For peer review only

Figure 1: Study Selection

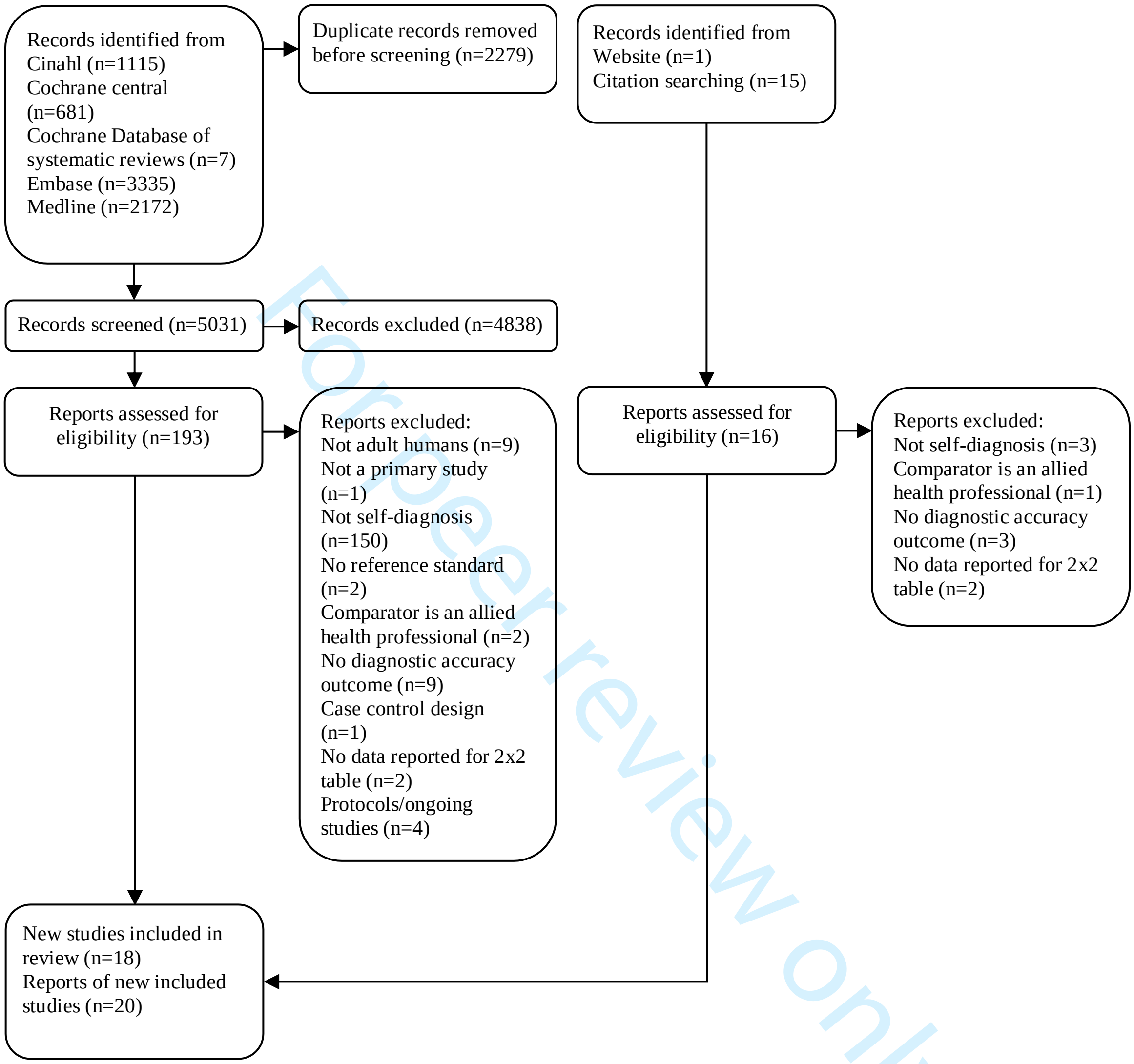

For peer review only

Figure 2: Paired forest plots of sensistivity and specificity for studies of self-diagnosis of vaginal infection (where Donders 2016a used pH threshold ≥ 4.5, Donders 2016b used pH threshold ≥ 4.7. Sungkar 2012 represents pooled data from 4 time points)

Study	TP	FP	FN	TN	Sensitivity (95% CI)	Specificity (95% CI)
**Bacterial vaginosis**						
Donders 2016a	80	151	4	103	0.95 [0.88, 0.99]	0.41 [0.34, 0.47]
Donders 2016b	38	49	46	205	0.45 [0.34, 0.56]	0.81 [0.75, 0.85]
Sungkar 2012	8	16	14	122	0.36 [0.17, 0.59]	0.88 [0.82, 0.93]
**Bacterial vaginosis &/or Trichomonas vaginalis**						
Geva 2006	226	50	23	217	0.91 [0.86, 0.94]	0.81 [0.76, 0.86]
Ryan-Wenger 2010	193	109	127	107	0.60 [0.55, 0.66]	0.50 [0.43, 0.56]
**Candida vaginitis**						
Ryan-Wenger 2010	26	43	122	345	0.18 [0.12, 0.25]	0.89 [0.85, 0.92]
**Trichomonas vaginalis**						
Jones 2013	13	1	6	675	0.68 [0.43, 0.87]	1.00 [0.99, 1.00]

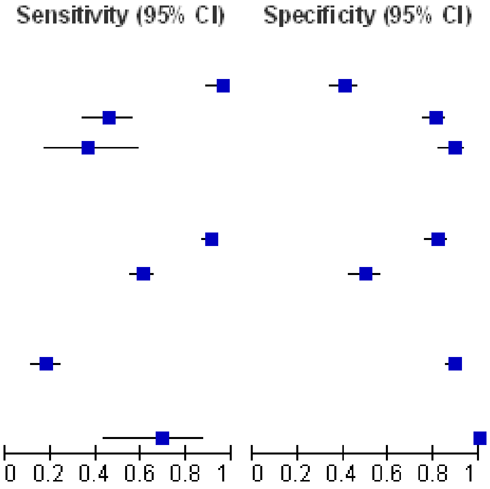

For peer review only

Figure 3: Paired forest plots of sensistivity and specificity for studies of self-diagnosis of common skin conditions (where Bregnhoj 2011 represents pooled data from recruitment and follow up time points)

Study	TP	FP	FN	TN	Sensitivity (95% CI)	Specificity (95% CI)
**Eczema**						
Bregnhoj 2011	26	1	11	726	0.70 [0.53, 0.84]	1.00 [0.99, 1.00]
Svensson 2002	78	22	12	82	0.87 [0.78, 0.93]	0.79 [0.70, 0.86]
**Nickel & fragrance allergy**						
Elsner 2015	39	16	1	106	0.97 [0.87, 1.00]	0.87 [0.80, 0.92]
**Nickel allergy**						
Josefson 2011	33	13	13	132	0.72 [0.57, 0.84]	0.91 [0.85, 0.95]

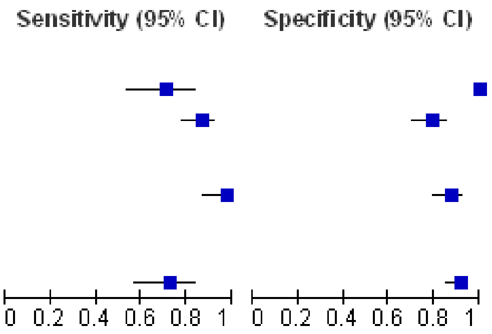

For peer review only

Figure 4: Studies of self-diagnosis of HIV

(a) Paired forest plots of sensitivity and specificity (where Choko 2015 represents pooled data from 1- 12 and 13 – 24 months follow up time points)

Study	TP	FP	FN	TN	Sensitivity (95% CI)	Specificity (95% CI)
Assiimwe 2014	24	5	3	91	0.89 [0.71, 0.98]	0.95 [0.88, 0.98]
Belete 2019	199	0	1	200	0.99 [0.97, 1.00]	1.00 [0.98, 1.00]
Choko 2011	27	0	1	210	0.96 [0.82, 1.00]	1.00 [0.98, 1.00]
Choko 2015	132	1	9	1507	0.94 [0.88, 0.97]	1.00 [1.00, 1.00]
Kapaku 2017	227	7	32	2286	0.88 [0.83, 0.91]	1.00 [0.99, 1.00]
Kurth 2016	26	1	3	173	0.90 [0.73, 0.98]	0.99 [0.97, 1.00]
Martinez Perez 2016	323	0	4	1860	0.99 [0.97, 1.00]	1.00 [1.00, 1.00]
Orasure 2012	106	1	8	5385	0.93 [0.87, 0.97]	1.00 [1.00, 1.00]
Pant Pai 2013	6	0	3	242	0.67 [0.30, 0.93]	1.00 [0.98, 1.00]

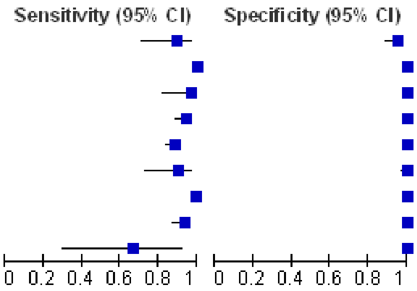

(b) Receiver operating characteristic plot HIV self-diagnosis compared with clinical diagnosis or laboratory reference test grouped by reference test type (where size of symbol indicates study size)

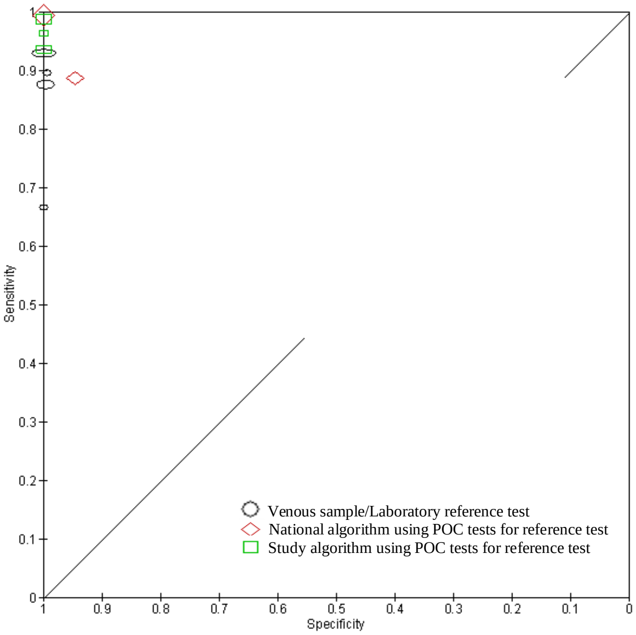

All studies (n=9):
Sensitivity: 92.8% (95%CI 86% to 96.5%); Specificity: 99.8% (95%CI 99.1% to 99.9%)
Sensitivity analysis retaining only studies with venous/laboratory reference test (n=4):
Sensitivity: 87.7% (95%CI 81.4% to 92.2%); Specificity: 99.8% (95%CI 98.9% to 99.9%)

For peer review only - http://bmjopen.bmj.com/site/about/guidelines.xhtml

<u>Figure 5: QUADAS-2 summary of risk of bias and applicability concerns showing review authors' judgments about each domain for each included study (based on 19 studies (20 data sets))</u>

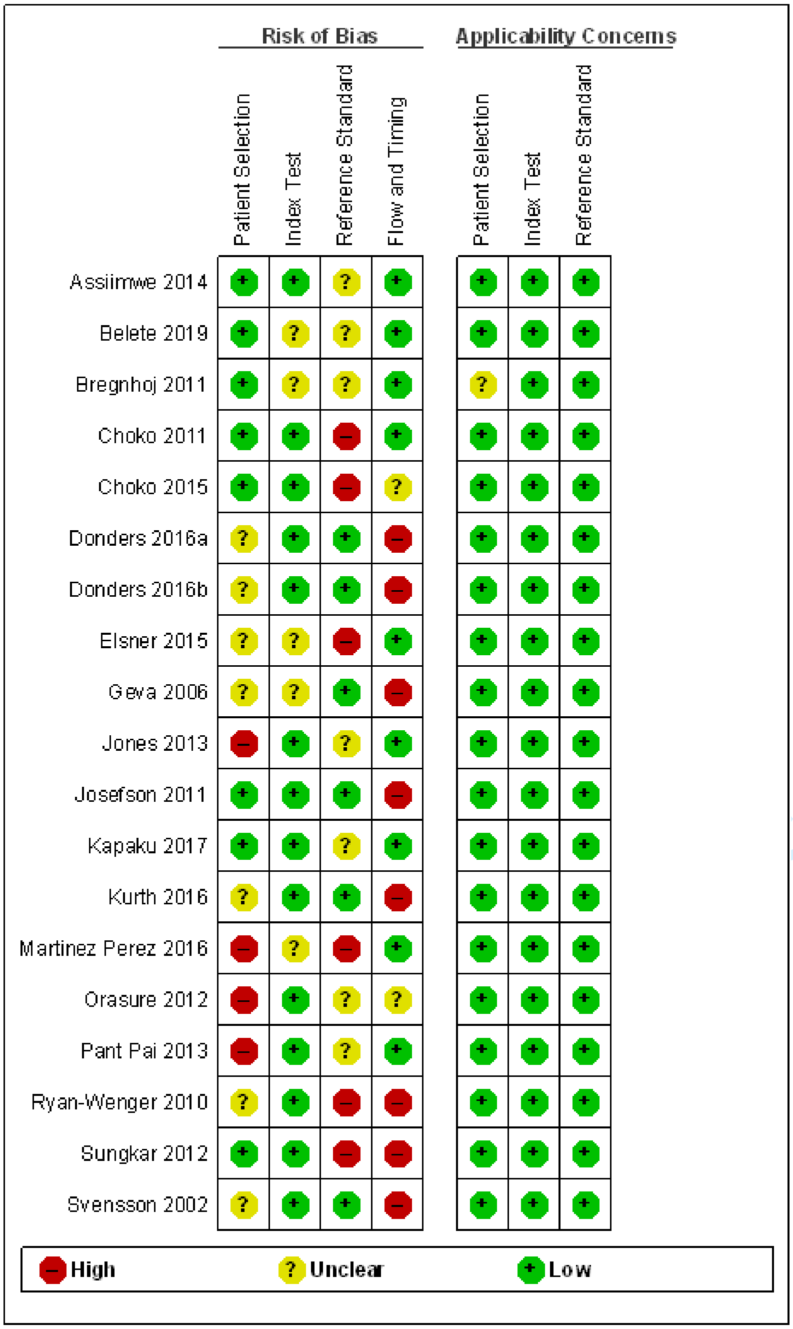

SUPPLEMENTARY MATERIAL

Supplementary Table 1: Commonly managed conditions in primary care

As reported by Cooke et al 2013 (Cooke G, Valenti L, Glasziou P, Britt H. Common general practice presentations and publication frequency. Australian Family Physician. 2013; 42:65-8)

1	Hypertension
2	Immunisation/vaccination: all
3	Acute upper respiratory tract infection
4	Depression
5	Diabetes: nongestational
6	Lipid disorders
7	General check-up
8	Osteoarthritis
9	Back complaint
10	Prescription
11	Oesophagus disease
12	Female genital check-up
13	Acute bronchitis/bronchiolitis
14	Asthma
15	Anxiety
16	Test results
17	Urinary tract infection
18	Dermatitis, contact/allergic
19	Pregnancy
20	Sleep disturbance
21	Sinusitis acute/chronic
22	Gastroenteritis
23	Vitamin/nutritional deficiency

For peer review only

24	Malignant neoplasm of skin
25	Abnormal test results
26	Atrial fibrillation/flutter
27	Oral contraception
28	Solar keratosis/sunburn
29	Ischaemic heart disease
30	Viral disease, not otherwise specified

For peer review only

Supplementary Table 2: Search strategy

**Medline**	
**#**	**Searches**
1	Diagnostic Self Evaluation/
2	((self* adj diagnos*) or selfdiagnos*).ti,ab.
3	(self* and diagnos*).ti.
4	((self* adj test*) or selftest*).ti,ab.
5	(home adj3 diagnos*).ti,ab.
6	((selfreport* or self-report*) and diagnos*).ti.
7	((selfreport* or self-report*) adj5 diagnos*).ti,ab.
8	(diagnos* and (selftreat* or self-treat*)).ti,ab.
9	1 or 2 or 3 or 4 or 5 or 6 or 7 or 8
10	respiratory tract infections/ or common cold/ or influenza, human/ or laryngitis/ or exp pharyngitis/ or rhinitis/ or exp sinusitis/ or exp supraglottitis/ or tracheitis/ or exp otitis media/
11	(((respirat* or airway*) adj2 infection*) or (common cold or influenza or flu or pharyngitis or laryngitis or tonsillitis or sore throat or rhinitis or nasopharyngitis or nasolaryngitis or sinusitis or supraglottitis or epiglottitis or tracheitis or urti or otitis media)).ti,ab.
12	exp Depressive Disorder/ or Depression/
13	(depress* or (mood adj2 disorder*)).ti,ab.
14	diabetes mellitus/ or exp diabetes mellitus, type 1/ or exp diabetes mellitus, type 2/
15	diabet*.ti,ab.
16	dyslipidemias/ or exp hyperlipidemias/
17	(dyslipid?emia? or hyperlipid?emia? or hypercholesterol?emia? or cholesterol* or triglyceride*).ti,ab.
18	hypertension/
19	(hypertens* or high blood pressure).ti,ab.
20	exp Osteoarthritis/
21	(osteoarthritis or osteo-arthritis or (degenerat* adj2 arthritis)).ti,ab.
22	back pain/ or low back pain/
23	Sciatica/
24	Intervertebral Disc Displacement/
25	((back adj2 (pain or problem? or disorder?)) or slipped disc* or sciatica).ti,ab.
26	Esophageal Diseases/ or esophageal motility disorders/ or exp gastroesophageal reflux/ or Barrett Esophagus/
27	((oesophag* or esophag*) adj2 disease).ti,ab.
28	(((oesophag* or esophag* or gastr*) adj2 reflux) or heartburn or heart burn).ti,ab.
29	((barrett* or globus) adj2 (esophag* or oesophag*)).ti,ab.
30	exp Asthma/
31	asthma*.ti,ab.

For peer review only

32	exp Anxiety Disorders/ or Anxiety/
33	(anxiety or phobia*).ti,ab.
34	exp Urinary Tract Infections/
35	exp Cystitis/
36	((urin* adj3 infection*) or bacteriuria or pyuria or cystitis).ti,ab.
37	exp Vaginitis/
38	(vaginitis or vaginosis).ti,ab.
39	dermatitis/ or exp dermatitis, contact/ or eczema/
40	(dermatitis or eczema).ti,ab.
41	exp Sleep Wake Disorders/
42	exp Sleep Apnea Syndromes/
43	((sleep adj2 (disorder* or disturbance*)) or insomnia*).ti,ab.
44	(sleep adj2 (apnea or apnoea)).ti,ab.
45	restless leg*.ti,ab.
46	Gastroenteritis/ or diarrhea/ or vomiting/
47	(gastroenteritis or (stomach adj2 (bug? or upset))).ti,ab.
48	(diarrhoea or diarrhea or food poisoning).ti,ab.
49	Sunburn/
50	(sunburn or solar keratosis).ti,ab.
51	or/10-50
52	9 and 51
53	exp "Sensitivity and Specificity"/
54	exp "REPRODUCIBILITY OF RESULTS"/
55	(sensitiv* or specific* or predict* or accura* or valid* or reproduc*).ti,ab.
56	53 or 54 or 55
57	52 and 56
**Embase**	
**#**	**Searches**
1	*Self Evaluation/
2	((self* adj diagnos*) or selfdiagnos*).ti,ab.
3	(self* and diagnos*).ti.
4	((self* adj test*) or selftest*).ti,ab.
5	(home adj3 diagnos*).ti,ab.
6	((selfreport* or self-report*) and diagnos*).ti.
7	((selfreport* or self-report*) adj5 diagnos*).ti,ab.
8	(diagnos* and (selftreat* or self-treat*)).ti,ab.
9	1 or 2 or 3 or 4 or 5 or 6 or 7 or 8
10	respiratory tract infection/ or upper respiratory tract infection/ or exp respiratory tract inflammation/ or influenza/ or otitis media/
11	(((respirat* or airway*) adj2 infection*) or (common cold or influenza or flu or pharyngitis or laryngitis or tonsillitis or sore throat or rhinitis or nasopharyngitis or nasolaryngitis or sinusitis or supraglottitis or epiglottitis or tracheitis or urti or otitis media)).ti,ab.

For peer review only

12	exp Depression/
13	(depress* or (mood adj2 disorder*)).ti,ab.
14	diabetes mellitus/ or exp diabetes mellitus, type 1/ or exp diabetes mellitus, type 2/
15	diabet*.ti,ab.
16	dyslipidemia/ or exp hyperlipidemia/
17	(dyslipid?emia? or hyperlipid?emia? or hypercholesterol?emia? or cholesterol* or triglyceride*).ti,ab.
18	hypertension/
19	(hypertens* or high blood pressure).ti,ab.
20	exp Osteoarthritis/
21	(osteoarthritis or osteo-arthritis or (degenerat* adj2 arthritis)).ti,ab.
22	backache/ or discogenic pain/ or low back pain/
23	Sciatica/
24	intervertebral disk hernia/ or intervertebral disk disease/
25	((back adj2 (pain or problem? or disorder?)) or slipped disc* or sciatica).ti,ab.
26	exp gastroesophageal reflux/ or barrett esophagus/ or esophagus disease/
27	((oesophag* or esophag*) adj2 disease).ti,ab.
28	(((oesophag* or esophag* or gastr*) adj2 reflux) or heartburn or heart burn).ti,ab.
29	((barrett* or globus) adj2 (esophag* or oesophag*)).ti,ab.
30	exp Asthma/
31	asthma*.ti,ab.
32	exp Anxiety Disorder/
33	(anxiety or phobia*).ti,ab.
34	exp Urinary Tract Infection/
35	Cystitis/
36	((urin* adj3 infection*) or bacteriuria or pyuria or cystitis).ti,ab.
37	vagina discharge/ or exp vaginitis/
38	(vaginitis or vaginosis).ti,ab.
39	dermatitis/ or contact dermatitis/ or exp eczema/
40	(dermatitis or eczema).ti,ab.
41	sleep disorder/ or exp insomnia/ or periodic limb movement disorder/
42	exp sleep disordered breathing/
43	((sleep adj2 (disorder* or disturbance*)) or insomnia*).ti,ab.
44	(sleep adj2 (apnea or apnoea)).ti,ab.
45	restless leg*.ti,ab.
46	Gastroenteritis/ or diarrhea/ or vomiting/
47	(gastroenteritis or (stomach adj2 (bug? or upset))).ti,ab.
48	(diarrhoea or diarrhea or food poisoning).ti,ab.
49	Sunburn/
50	(sunburn or solar keratosis).ti,ab.
51	or/10-50

For peer review only

52	9 and 51
53	"Sensitivity and Specificity"/
54	diagnostic accuracy/ or diagnostic test accuracy study/
55	predictive validity/ or predictive value/ or reproducibility/
56	(sensitiv* or specific* or predict* or accura* or valid* or reproduc*).ti,ab.
57	53 or 54 or 55 or 56
58	52 and 57
**Cochrane**	
**ID**	**Search**
#1	MeSH descriptor: [Diagnostic Self Evaluation] explode all trees
#2	(((self* AND diagnos*) or selfdiagnos*)):ti OR (((self* NEXT diagnos*) or selfdiagnos*)):ti,ab,kw OR (((self* NEXT test*) or selftest*)):ti,ab,kw OR (home NEAR/3 diagnos*):ti,ab,kw
#3	(((selfreport* or self-report*) and diagnos*)):ti OR (((selfreport* or self-report*) NEAR diagnos*)):ti,ab,kw OR ((diagnos* and (selftreat* or self-treat*))):ti,ab,kw
#4	#1 OR #2 OR #3
#5	MeSH descriptor: [Sensitivity and Specificity] explode all trees
#6	MeSH descriptor: [Reproducibility of Results] explode all trees
#7	(sensitiv* or specific* or predict* or accura* or valid* or reproduc*):ti,ab,kw
#8	#5 OR #6 OR #7
#9	#4 AND #8
**Cinahl**	
**#**	**Query**
S39	S3 AND S35 AND S38
S38	S36 OR S37
S37	TX sensitiv* or specific* or predict* or accura* or valid* or reproduc*
S36	(MH "Sensitivity and Specificity") OR (MH "Predictive Value of Tests")
S35	(S4 OR S5 OR S6 OR S7 OR S8 OR S9 OR S10 OR S11 OR S12 OR S13 OR S14 OR S15 OR S16 OR S17 OR S18 OR S19 OR S20 OR S21 OR S22 OR S23 OR S24 OR S25 OR S26 OR S27 OR S28 OR S29 OR S30 OR S31 OR S32 OR S33 OR S34)
S34	TI (sunburn or "solar keratosis") OR AB (sunburn or "solar keratosis")
S33	(MH "Sunburn")
S32	TI ((gastroenteritis or (stomach N2 (bug or bugs or upset)))) OR AB ((gastroenteritis or (stomach N2 (bug or bugs or upset)))) OR TI (diarrhoea or diarrhea or "food poisoning") OR AB (diarrhoea or diarrhea or "food poisoning")
S31	(MH "Gastroenteritis") OR (MH "Vomiting") OR (MH "Diarrhea")
S30	TI (((sleep N2 (disorder* or disturbance*)) or insomnia*)) OR AB (((sleep N2 (disorder* or disturbance*)) or insomnia*)) OR TI ((sleep N2 (apnea or apnoea))) OR AB ((sleep N2 (apnea or apnoea))) OR TI "restless legs" OR AB "restless legs"
S29	(MH "Sleep Disorders, Intrinsic+")
S28	TI (dermatitis or eczema) OR AB (dermatitis or eczema)
S27	(MH "Dermatitis") OR (MH "Dermatitis, Contact+") OR (MH "Eczema")
S26	TI (vaginitis or vaginosis) OR AB (vaginitis or vaginosis)

For peer review only

S25	(MH "Vaginitis+")
S24	TI (((urin* N3 infection*) or bacteriuria or pyuria or cystitis)) OR AB (((urin* N3 infection*) or bacteriuria or pyuria or cystitis))
S23	(MH "Urinary Tract Infections+") OR (MH "Cystitis")
S22	TI asthma* OR AB asthma*
S21	(MH "Asthma+")
S20	TI (((oesophag* or esophag*) N2 disease)) OR (((oesophag* or esophag*) N2 disease)) OR TI ((((oesophag* or esophag* or gastr*) N2 reflux) or heartburn or "heart burn")) OR AB ((((oesophag* or esophag* or gastr*) N2 reflux) or heartburn or "heart burn")) OR (((barrett* or globus) N2 (esophag* or oesophag*))) OR (((barrett* or globus) N2 (esophag* or oesophag*)))
S19	(MH "Gastroesophageal Reflux") OR (MH "Esophageal Motility Disorders") OR (MH "Esophageal Diseases") OR (MH "Barrett Esophagus")
S18	TI (((back N2 (pain or problem* or disorder*)) or "slipped disc*" or sciatica)) OR AB (((back N2 (pain or problem* or disorder*)) or "slipped disc*" or sciatica))
S17	(MH "Low Back Pain") OR (MH "Back Pain") OR (MH "Sciatica") OR (MH "Intervertebral Disk Displacement")
S16	TI ((osteoarthritis or osteo-arthritis or (degenerat* N2 arthritis))) OR AB ((osteoarthritis or osteo-arthritis or (degenerat* N2 arthritis)))
S15	(MH "Osteoarthritis+")
S14	TI (hypertens* or "high blood pressure") OR AB (hypertens* or "high blood pressure")
S13	(MH "Hypertension")
S12	TI (dyslipidemia* or hyperlipidemia* or hypercholesterolemia* or dyslipidaemia* or hyperlipidaemia* or hypercholesterolaemia* or cholesterol* or triglyceride*) OR AB (dyslipidemia* or hyperlipidemia* or hypercholesterolemia* or dyslipidaemia* or hyperlipidaemia* or hypercholesterolaemia* or cholesterol* or triglyceride*)
S11	(MH "Hyperlipidemia+")
S10	TI diabet* OR AB diabet*
S9	(MH "Diabetes Mellitus") OR (MH "Diabetes Mellitus, Type 1") OR (MH "Diabetes Mellitus, Type 2")
S8	TI ((depress* OR anxiety or (mood N2 disorder*))) OR AB ((depress* OR anxiety or (mood N2 disorder*)))
S7	(MH "Depression") OR (MH "Anxiety")
S6	TI ((((respirat* or airway*) N2 infection*) or ("common cold" or influenza or flu or pharyngitis or laryngitis or tonsillitis or "sore throat" or rhinitis or nasopharyngitis or nasolaryngitis or sinusitis or supraglottitis or epiglottitis or tracheitis or urti or "otitis media"))) OR AB ((((respirat* or airway*) N2 infection*) or ("common cold" or influenza or flu or pharyngitis or laryngitis or tonsillitis or "sore throat" or rhinitis or nasopharyngitis or nasolaryngitis or sinusitis or supraglottitis or epiglottitis or tracheitis or urti or "otitis media")))
S5	(MH "Otitis Media+")
S4	(MH "Respiratory Tract Infections") OR (MH "Common Cold") OR (MH "Influenza") OR (MH "Influenza, Human+") OR (MH "Laryngitis+") OR (MH "Pharyngitis") OR (MH "Rhinitis+") OR (MH "Sinusitis+") OR (MH "Tonsillitis+")

For peer review only

S3	S1 OR S2
S2	TI (((self* AND diagnos*) or selfdiagnos*)) OR AB (((self* N1 diagnos*) or selfdiagnos*)) OR TI (((self* N1 test*) or selftest*)) OR AB (((self* N1 test*) or selftest*)) OR TI (home N3 diagnos*) OR AB (home N3 diagnos*) OR TI (((selfreport* or self-report*) and diagnos*)) OR AB (((selfreport* or self-report*) N5 diagnos*)) OR TI ((diagnos* and (selftreat* or self-treat*))) OR AB ((diagnos* and (selftreat* or self-treat*)))
S1	(MH "Self Diagnosis")

For peer review only

Supplementary table 3: Data items included in extraction sheet (where available)

Study identification - author, year, location
Study research question
Study design and setting
Study funding source
Target condition definition/diagnostic criteria
Participant characteristics and numbers, including exclusions
Index test
Reference standard
Flow of participants through study including losses to follow-up<br>Patient presentation and prior testing
Conduct of the study including timing of the tests, and information on masking
Absolute counts of true positive (TP), false positive (FP), false negative (FN) and true negative (TN) diagnoses.
Statistical analyses that were performed, including whether all participants were included in analyses
Additional summary information on participant preference, timing, or cost, as available.

For peer review only

Supplementary table 4: Protocol

Accuracy of self-diagnosis in conditions commonly managed in primary care: diagnostic accuracy review

Annette Plüddemann [1], Hayley E Jones [2], Carl Heneghan [1]

1. Nuffield Department of Primary Care Health Sciences, University of Oxford, OX2 6GG
2. Population Health Sciences, Bristol Medical School, University of Bristol, BS8 2PS

Background and rationale

A wide range of conditions present to primary care, some acute, some chronic. As a consequence primary care is facing increasing workload (Hobbs et al 2016) that may become unmanageable.

Some common conditions in primary care, therefore, have the potential to be self-diagnosed and self-treated by the patients themselves. This offers superior convenience for individuals, swifter diagnosis and treatment where relevant, reduced costs for health service providers and
potentially reduce the burden on primary care services. Self-diagnosis may apply to initial diagnosis, or to diagnosing an exacerbation of an ongoing condition, such as an exacerbation of chronic obstructive pulmonary disease (COPD). In order to support self-diagnosis where safe and appropriate, the efficacy of self-diagnosis needs to be assessed, and this information used to make evidence-based decisions on who can self-diagnose safely.

Where there is an available comparison with diagnosis by a healthcare professional, it is possible to assess the accuracy of self-diagnosis. A further comparison could potentially be made with diagnosis by an allied healthcare professional such as pharmacists, but this is outside the scope of this current review. Sometimes, self-diagnosis involves using a diagnostic test (any type of medical test used to help diagnose or detect disease). For conditions where this is available and appropriate, the accuracy of these tests also informs the safety and efficacy of self-diagnosis by the patient.

Previous studies on the safety and accuracy of self-diagnosis of conditions commonly managed in primary care setting include self-diagnosis of urinary tract infection (Donofrio & Weiner 2013), high blood pressure (Tormo et al 2000) and depression (Hedayati et al 2006).

This review aims to identify, appraise and summarise the available evidence on self-diagnosis in common conditions in primary care. Cooke and colleagues recently reported the 30 most commonly managed conditions in primary care in Australia, which has a health landscape broadly comparable with western Europe (Cooke et al 2013). This list arises from survey data collected between January 2009 and December 2010, which included 194,100 patient encounters from 1,941 GPs. The most commonly managed conditions included some with the potential for self-diagnosis, e.g. urinary tract infection, as well as some that would be unsuitable for self-diagnosis, such as "general check-up". We base our review on conditions

For peer review only

from this list that are relevant for self-diagnosis. We may review infectious diseases and non-communicable diseases separately.

Objectives

Primary objective

Our primary objective is to summarize the accuracy of self-diagnosis of common conditions in primary care, compared with diagnosis by a healthcare provider.

Secondary objective

To summarise any associated relevant information relating to self-diagnosis of common conditions in primary care, such as information on patient preference, timing, or cost (only using information from studies we include for accuracy data). Where there is substantial qualitative information reported, this will only be summarised briefly; detailed qualitative approaches will not be used.

Methods

Criteria for considering studies for this review

Types of studies

Prospective or retrospective studies comparing the results of self-diagnosis of common self-limiting conditions in primary care by free-living individuals, to the results of a reference standard test performed by a healthcare service provider, will be included. Studies with a case-control design will be excluded. In case of duplicate publications we will include the study report with the highest methodological quality. There will be no language restrictions.

We will exclude studies comparing self-diagnosis with diagnosis by allied health professionals such as a pharmacists.

Participants

Adults (>= 18 years of age) self-diagnosing conditions common in primary care.

Index tests

Index tests will be the self-testing or self-diagnosis of relevant conditions, compared with diagnosis by a healthcare practitioner.

Comparator tests

Comparator tests will comprise diagnosis by a healthcare practitioner.

Outcome measures

Diagnostic accuracy measures (e.g. sensitivity, specificity, likelihood ratios, predictive values, etc.) and primary data for 2x2 tables. Studies reporting only measures of agreement will be excluded.

For peer review only - http://bmjopen.bmj.com/site/about/guidelines.xhtml

Search methods to identify studies

Electronic searches

The search strategy will be developed in consultation with a healthcare librarian experienced with supporting systematic reviews. No language restrictions will be applied. The search strategy will use multiple electronic databases, from inception onwards including:

Medline

EMBASE

Cochrane Central Register of Controlled Trials (CENTRAL)

Trip database

Web of Science for conference proceedings, dissertations, and theses

World Health Organization International Clinical Trials Registry Platform (ICTRP),

ClinicalTrials.gov

Database of Abstracts of Reviews of Effect (DARE)

We will also search Science Citation Index Expanded for study reports that cite the included studies.

The search may use relevant filters, but in order to maximise sensitivity, will not be limited to these. The reference lists of relevant studies will be examined and additional tools such as the "related articles" feature in PubMed will also be used to identify relevant publications.

Data collection and analysis

Selection of studies

Two reviewers will independently apply the selection criteria to the titles and abstracts of the study reports identified by the searches. If the decision to exclude a study cannot be made on the basis of the title and the abstract, the full study report will be retrieved for inclusion assessment. The final decision on inclusion will be based on the full study report. Disagreements between reviewers will be resolved by discussion, or if necessary by a third reviewer. Study identification will be summarised in a PRISMA flow diagram.

Data extraction and management

Two reviewers will independently extract information from selected studies into a data extraction sheet. Disagreements will be resolved by discussion, or if necessary with the help of a third reviewer.

Where this is insufficient (or unclear) information, where there is an email address provided, the authors will be contacted via email for clarification. Where data is not available for completion of 2x2 tables, the studies will be excluded from the analysis.

Data to be extracted

For peer review only

The following information will be extracted from the included studies, where available:

Study identification - author, year, location

Study research question

Study design and setting

Target condition definition/diagnostic criteria

Participant characteristics and numbers, including exclusions

Index test

Reference standard

Flow of participants through study including losses to follow-up

Patient presentation and prior testing

Conduct of the study including timing of the tests, and information on masking

Absolute counts of true positive (TP), false positive (FP), false negative (FN) and true negative (TN) diagnoses.

Statistical analyses that were performed, including whether all participants were included in analyses

Additional summary information on participant preference, timing, or cost, as available.

Assessment of methodological quality

To assess methodological quality, we will use the Quality Assessment of Diagnostic Accuracy Studies-2 (QUADAS-2) tool (Whiting et al 2011). Two reviewers will independently assess studies' methodological quality; disagreements will be resolved by discussion, or if necessary, by a third reviewer. The QUADAS-2 tool facilitates assessment of bias in four areas: patient selection; index test; reference standard; flow and timing; and also facilitates assessment of applicability of the studies to the review research question.

The data will be presented in a tables showing risk of bias and applicability within each domain assessed for each study. These data will be considered in relation to interpreting the results of the studies.

Statistical analysis and data synthesis

Analyses will be conducted for each category of condition specified. Summary tables will detail study information including the patient sample, condition, study design, the test under evaluation, and the comparator.

Meta-analysis

For each test, RevMan will be used to produce paired forest plots to explore the between-study variability of sensitivity and specificity across the included studies. For each study estimate of sensitivity and specificity, corresponding 95% confidence intervals will be shown

For peer review only

to illustrate the uncertainty related to each study estimate. If accuracy has been reported at multiple common thresholds, forest plots will be sub-grouped on threshold.

Bivariate meta-analysis methods (Reitsma et al 2005) will be used to generate pooled estimates of sensitivity and specificity where sufficient data is available for each test or condition. These will be plotted with 95% confidence and prediction ellipses in Receiver Operating Characteristic (ROC) space. Where appropriate, summary ROC curves will also be plotted, drawing on the equivalence of the bivariate method and the hierarchical summary ROC meta-analysis model (Rutter and Gatsonis 2001; Harbord et al 2007). For these analyses, we will use WinBUGS or the metandi command in Stata, as appropriate, and feed parameters directly into Revman to produce Cochrane-standardised output.

Where appropriate, meta-analysis models that include multiple thresholds will be employed (e.g. Steinhauser et al 2016 or similar).

Investigating heterogeneity

For medical conditions for which data from more than one study are available, it may be possible to investigate heterogeneity in the results. Two approaches will be used to explore the sources of between-study heterogeneity: 1) inclusion of study level characteristics as covariates in the bivariate model (meta-regression) 2) carrying out sub-group analyses. These approaches will only be carried out if there is sufficient data available and sub-group specific pooled estimates are thought to be of clinical relevance. Any meta-regressions will be carried out using WinBUGS or the xtmelogit command in Stata.

Sensitivity analyses

If there appear to be any outliers in the data, these studies will be removed from the analysis to evaluate the impact on the overall pooled estimates.

Investigating reporting bias

Funnel plots used to detect publication bias in reviews of RCTs have been shown to be misleading for diagnostic test accuracy reviews (Deeks et al 2005; Leeflang et al 2008). Funnel plots as an assessment of reporting bias will therefore not be included in this review. Publication bias will be assessed using Deek’s test, as recommended by the Cochrane Handbook, where data allows (Deeks et al 2005).

Funding

This project is funded by National Institute for Health Research School for Primary Care Research (NIHR SPCR) [ProjectNumber 390]

Declarations of conflict of interest

Dr. Plüddemann reports grants from NIHR, grants from NIHR School of Primary Care Research, during the conduct of the study; and occasionally receives expenses for teaching Evidence-Based Medicine. Dr. Heneghan reports receiving expenses and fees for his media work. He has received expenses from the WHO and holds grant funding from the NIHR, the NIHR School of Primary Care Research, The Wellcome Trust and the WHO. He has received financial remuneration from an asbestos case. He has also received income from the

For peer review only

publication of a series of toolkit books published by Blackwells. On occasion, he receives expenses for teaching EBM and is also paid for his GP work in NHS out of hours. CEBM jointly runs the EvidenceLive Conference with the BMJ and the Overdiagnosis Conference with some international partners which are based on a non-profit making model.

References

Cooke G, Valenti L, Glasziou P, Britt H. Common general practice presentations and publication frequency. Aust Fam Physician. 2013;42(1-2):65-8.

Deeks JJ, Macaskill P, Irwig L. The performance of tests of publication bias and other sample size effects in systematic reviews of diagnostic test accuracy was assessed. J Clin Epidemiol. 2005;58(9):882-93

Donofrio JC, Weiner SG. Female patient self-diagnosis compared with emergency physician diagnosis of urinary tract infection. J Emerg Med. 2013;45(6):969-73. doi: 10.1016/j.jemermed.2013.07.019

Harbord RM, Deeks JJ, Egger M, Whiting P, Sterne JA. A unification of models for meta-analysis of diagnostic accuracy studies. Biostatistics. 2007 Apr;8(2):239-51.

Hedayati SS, Bosworth HB, Kuchibhatla M, Kimmel PL, Szczech LA. The predictive value of self-report scales compared with physician diagnosis of depression in hemodialysis patients. Kidney Int. 2006 May;69(9):1662-8.

Hobbs FDR, Bankhead C, Mukhtar T, Stevens S, Perera-Salazar R, Holt T, Salisbury C; National Institute for Health Research School for Primary Care Research. Clinical workload in UK primary care: a retrospective analysis of 100 million consultations in England, 2007–14. The Lancet; 387; 10035: 2323-2330

Leeflang MM, Deeks JJ, Gatsonis C, Bossuyt PM; Cochrane Diagnostic Test Accuracy Working Group. Systematic reviews of diagnostic test accuracy. Ann Intern Med. 2008; 16;149(12): 889-97

Rutter CM, Gatsonis CA. A hierarchical regression approach to meta-analysis of diagnostic test accuracy evaluations. Stat Med. 2001 Oct 15;20(19):2865-84.

Reitsma JB, Glas AS, Rutjes AW, Scholten RJ, Bossuyt PM, Zwinderman AH. Bivariate analysis of sensitivity and specificity produces informative summary measures in diagnostic reviews. J Clin Epidemiol. 2005 Oct;58(10):982-90

Review Manager (RevMan) [Computer program]. Version 5.3. Copenhagen: The Nordic Cochrane Centre, The Cochrane Collaboration, 2014

Steinhauser S, Schumacher M, Rücker G. Modelling multiple thresholds in meta-analysis of diagnostic test accuracy studies. BMC Med Res Methodol. 2016 Aug 12;16(1):97. doi: 10.1186/s12874-016-0196-1.

Tormo MJ, Navarro C, Chirlaque MD, Barber X. Validation of self diagnosis of high blood pressure in a sample of the Spanish EPIC cohort: overall agreement and predictive values. EPIC Group of Spain. J Epidemiol Community Health. 2000 Mar;54(3):221-6.

For peer review only

Supplementary Table 5: Characteristics of included studies

Disease/ condition	Author, year	Design	Setting	Country	Study duration	Number of participants	Mean age* (years)	Female (%)
Vaginal infection	Donders 2016	Prospective diagnostic accuracy study	Birth control, general gynaecology, infertility and prenatal clinics	Uganda	N/A	360	28.3	100
	Sungkar 2012	RCT (intervention arm only)	Prenatal clinics or hospitals	Indonesia	24 weeks	176	28	100
	Geva 2006	Prospective diagnostic accuracy study	Gynaecologic clinics	Israel	N/A	593	18 – 60 range	100
	Ryan-Wenger 2010	Prospective diagnostic accuracy study	Military clinics	USA	N/A	546	25.7	100
	Jones 2013	RCT (both arms)	Clinics or home	Brazil	N/A	695	18 – 40 range	100
Common skin condition	Bregnhoj 2011	Prospective cohort	Not reported	Denmark	18 months	502	17.5	95
	Svensson 2002	Prospective diagnostic accuracy study	Dermatology outpatient clinics	Sweden	N/A	208	40.4	50
	Elsner 2015	Prospective diagnostic accuracy study	Not reported	Germany & Austria	N/A	165	≥18 years	81
	Josefson 2011	Prospective diagnostic accuracy study	Hospital dermatology departments	Sweden	N/A	243	44	69
HIV	Assiimwe 2014	RCT (unsupervised arm only)	Home	Uganda	N/A	123	28 (23–32), median (IQR)	47
	Belete 2019	Cross-sectional	Public health facilities	Ethiopia	N/A	400	29 (17.7–40.3), median (IQR)	61
	Choko 2011	Cross-sectional	Home	Malawi	N/A	241	27 (NR) median (IQR)	52
	Choko 2015	RCT (intervention arm only)	Home	Malawi	2 years	2370	NR	NR

For peer review only

	Kapaku 2017	Cohort	Home/Voluntary counselling & testing facilities	Zambia	1 year	2572	26 (21–35), median (IQR)	59
	Kurth 2016	Cross-sectional	NR (not at home)	Kenya	N/A	240**	36	33
	Martinez Perez 2016	Cross-sectional	Health care clinics/HIV testing sites	South Africa	N/A	2205	Male: 27 (11-36) Female: 28 (22-36) median (IQR)	66
	Orasure 2012	Cross-sectional	Study site	USA	N/A	5798	NR	50
	Pant Pai 2013	Cross-sectional	Hospital	South Africa	N/A	251	≥18 years	79

RCT – randomised control trial; N/A – not applicable; NR – not reported, * unless otherwise indicated, ** Subset (N=113) that used laboratory reference standard included in systematic review and pooled analysis.

For peer review only

Supplementary Table 6: Characteristics of self-diagnosis (index) tests and reference standard tests

Disease/ condition	Author, year	Test for	Self-diagnosis test (index)	Self-diagnosis test threshold	Reference test	Reference test threshold	Interval between index & reference test	Data collection points
Vaginal infection	Donders 2016a	Bacterial vaginosis	Vaginal fluid test using pH strip	+/- (≥ 4.5 pH)	Air dried vaginal fluid test using gram staining (Nugent score) Assessment by central laboratory	+/- (≥ 4.5 pH, Nugent score 7-10)	NR	N/A
	Donders 2016b			+/- (≥ 4.7 pH)		+/- (≥ 4.7 pH, Nugent score 7-10)		
	Sungkar 2012	Bacterial vaginosis	Vaginal fluid test using pH strip	+/-	Vaginal fluid test using microbiological gram staining test (Kopeloff modified Gram stain) Prepared by midwives for laboratory assessment	+/-	NR	Baseline, 16 – 18, 18 – 20, 20 – 22, 22 – 24 weeks
	Geva 2006	Bacterial vaginosis and/or *Trichomonas vaginalis*	Vaginal discharge test using panty liner test kit (VI-SENSE)	+/- (based on strip colour, no level reported*)	Clinical diagnosis plus vaginal wall swabs testing pH (nitrazine paper), amine, culture (InPouch TV, BioMed Diagnostic) and gram staining (Nugent score). Assessment by board certified gynaecologists and central laboratory.	BV: >3: (a) homogeneous discharge, (b) pH value >4.5, (c) release of fishy odor (KOH was added to the vaginal discharge, and (d) presence of clue cells; or 7+ Nugent score of gram stain TV: +/- by culture	Within 6 hours	N/A
	Ryan-Wenger 2010	Bacterial vaginosis and/or *Trichomonas vaginalis*	Vaginal fluid test using Women in the military self-diagnosis kit	+/- (based on ≥ 4.7 pH, amines,	Clinical interview plus vaginal fluid test for pH (nitrazine paper), amines (FemExam card), whiff, wet mount	+/-	None	N/A

			(includes FemExam card)	vaginal itching)	microscopy (Affirm VPIII Microbial Identification Test)<br>Assessment by women's health nurse practitioner plus in clinic microscopy testing			
	Ryan-Wenger 2010	*Candida* vaginitis	Vaginal fluid test using Women in the military self-diagnosis kit (includes FemExam card)	+/- (based on ≥ 4.7 pH, amines, vaginal itching)	Clinical interview plus vaginal fluid test for pH (nitrazine paper, amines (FemExam card), whiff, wet mount microscopy (Affirm VPIII Microbial Identification Test)<br>Assessment by women's health nurse practitioner plus in clinic microscopy testing	+/-	None	N/A
	Jones 2013	*Trichomonas vaginalis*	Vaginal fluid testing using dipstick test (OSOM Trichomonas rapid test)	+/- (two red lines)	Vaginal fluid test using PCR test<br>Assessment at central laboratory	+/-	None	N/A
Common skin condition	Bregnhoj 2011	Eczema	Questionnaire on presence of eczema	Positive response	Hand Eczema Severity Index (HECSI)<br>Interpretation by clinician	+ve for presence of eczema	Same day	Inclusion & 18 month follow-up
	Svensson 2002	Hand eczema	Questionnaire on presence of eczema	Positive response	Hand examination for erythema, papules, vesicles, scaling, fissures, lichenification and hyperkeratotic areas.<br>Assessment by experienced dermatologist	+ve if erythema and papules / vesicles OR erythema and scaling and fissures / lichenification	None	N/A
	Elsner 2015	Allergic reaction to nickel and fragrance	Irritant reaction to surgical tapes on upper arm after 48 hours	+/-	Irritant reaction to surgical tapes on upper arm after 48 hours<br>Assessment by clinician	+/-	None	N/A
	Josefson 2011	Allergic reaction to nickel	Medical plaster patches (Nixema) on upper arm with	+/-	Medical plaster patches (Finn	+/-	Same day	N/A

For peer review only

			readings on days 3-4		chambers® on Scanpor® tape or IQ Ultra® Chambers) on back with readings on days 3-4 and/or day 7 Assessment by dermatologist			
HIV	Assiimwe 2014	Antibodies for HIV1 and HIV2	Oral fluid test using OraQuick® In-Home Rapid HIV-1/2 Antibody Test (Orasure Technologies)	+/-	Finger pick blood test. Nationally approved algorithm of POC rapid HIV tests (Determine (Abbot Laboratories), STAT-PAK (Chembio Diagnostic Systems Inc) and Unigold (Trinity Biotech plc) as a tiebreaker). Assessment by research assistants.	+/-	12 -72 hours	N/A
	Belete 2019	Antibodies for HIV1 and HIV2	Oral fluid test using OraQuick® Rapid HIV-1/2 Antibody Test (Orasure Technologies)	+/-	Finger pick blood test. Nationally approved algorithm of POC rapid HIV tests (Wanti (Beijing), Unigold (Trinity Biotech plc), Vikia). Assessment by health professional.	+/-	Same time	N/A
	Choko 2011	Antibodies for HIV1 and HIV2	Oral fluid test using OraQuick ADVANCE® Rapid HIV-1/2 Antibody Test	+/-	Finger pick blood test. Algorithm of POC rapid HIV tests (Determine (Abbot Laboratories), Unigold (Trinity Biotech plc) and SD Bioline HIV i/II (Standard Diagnostics, Inc.) as a tiebreaker). Assessment by counsellor.	+/-	Same time	N/A
	Choko 2015	Antibodies for HIV1 and HIV2	Oral fluid test using OraQuick ADVANCE® Rapid HIV-1/2 Antibody Test	+/-	Finger pick blood test. Two parallel POC rapid HIV tests (Determine (Abbot Laboratories), Unigold (Trinity Biotech plc)). Assessment by nurse.	+/-	Approx. 1 week	1 – 12, 13 – 24 months (max. 1 test in 12 months)

For peer review only

	Kapaku 2017	Antibodies for HIV1 and HIV2	Oral fluid test using OraQuick® rapid HIV-1/2 Antibody Test (Orasure Technologies)	+/-	Venous blood test (EDTA tube) Testing in certified central laboratory (Abbott Architect HIV1 Ag/Ab combo assay, positive results confirmed by BioRad GS HIV combo Ag/Ab assay)	+/-	Within 8 hours	Once in 12 month study period.
	Kurth 2016	Antibodies for HIV1 and HIV2	Oral fluid test using OraQuick ADVANCE® Rapid HIV-1/2 Antibody Test	+/-	Venous blood test. ELISA testing by single person in certified central laboratory (Vironostika HIV Uni-Form II Ag/Ab (bioMe´rieux Inc.))	+/-	Within 8 hours	N/A
	Martinez Perez 2016	Antibodies for HIV1 and HIV2	Oral fluid test using OraQuick ADVANCE® Rapid HIV-1/2 Antibody Test	+/-	Finger pick blood test. POC rapid HIV tests (Determine (Abbot Laboratories), confirmatory Unigold (Trinity Biotech plc)). Assessment by HIV counsellor.	+/-	Same time	N/A
	Orasure 2012	Antibodies for HIV1 and HIV2	Oral fluid test using OraQuick® In-Home Rapid HIV-1/2 Antibody Test (Orasure Technologies)	+/-	Venous blood test. FDA approved serum EIA and Western blot in FDA approved laboratory.	+/-	Unclear	N/A
	Pant Pai 2013	Antibodies for HIV1 and HIV2	Oral fluid test using OraQuick® rapid HIV-1/2 Antibody Test (Orasure Technologies)	+/-	Venous blood test. ELISA with p24 Antigen tests in reference laboratories all within 24 hours (Architect HIV Ag/Ab combo (Abbott Laboratories), positive results confirmed by Western Blot)	+/-	Same time	N/A

HIV - Human immunodeficiency virus, POC – Point of care, max. – maximum, FDA – Food and Drug Administration, USA, N/A – not applicable; NR – not reported; KOH – potassium hydroxide; PCR – polymerase chain reaction; * whilst no reported level was given for VI-SENSE, it was reported that the polymer used in this product had a range of 4.3 – 5.1 pH

PRISMA 2020 Checklist

Section and Topic	Item #	Checklist item	Location where item is reported
**TITLE**			
Title	1	Identify the report as a systematic review.	Title page (pg 1)
**ABSTRACT**			
Abstract	2	See the PRISMA 2020 for Abstracts checklist.	Page 2
**INTRODUCTION**			
Rationale	3	Describe the rationale for the review in the context of existing knowledge.	Introduction (pg 4)
Objectives	4	Provide an explicit statement of the objective(s) or question(s) the review addresses.	Introduction (pg 2)
**METHODS**			
Eligibility criteria	5	Specify the inclusion and exclusion criteria for the review and how studies were grouped for the syntheses.	Methods, Types of studies (pg 4) & Statistical analysis and data analysis (pg 5)
Information sources	6	Specify all databases, registers, websites, organisations, reference lists and other sources searched or consulted to identify studies. Specify the date when each source was last searched or consulted.	Methods, Search methods to identify studies (pg 4)
Search strategy	7	Present the full search strategies for all databases, registers and websites, including any filters and limits used.	Supplementary table 1
Selection process	8	Specify the methods used to decide whether a study met the inclusion criteria of the review, including how many reviewers screened each record and each report retrieved, whether they worked independently, and if applicable, details of automation tools used in the process.	Methods, Selection of studies (pg 5). No automation tools used.
Data collection process	9	Specify the methods used to collect data from reports, including how many reviewers collected data from each report, whether they worked independently, any processes for obtaining or confirming data from study investigators, and if applicable, details of automation tools used in the process.	Methods, Data extraction and management (pg 5). No automation tools used.
Data items	10a	List and define all outcomes for which data were sought. Specify whether all results that were compatible with each outcome domain in each study were sought (e.g. for all measures, time points, analyses), and if not, the methods used to decide which results to collect.	Supplementary table 2. Methods, Data extraction and

For peer review only - http://bmjopen.bmj.com/site/about/guidelines.xhtml

PRISMA 2020 Checklist

Section and Topic	Item #	Checklist item	Location where item is reported
			management (pg 5).
	10b	List and define all other variables for which data were sought (e.g. participant and intervention characteristics, funding sources). Describe any assumptions made about any missing or unclear information.	Supplementary table 2.<br>Methods, Data extraction and management (pg 5).
Study risk of bias assessment	11	Specify the methods used to assess risk of bias in the included studies, including details of the tool(s) used, how many reviewers assessed each study and whether they worked independently, and if applicable, details of automation tools used in the process.	Methods, Assessment of methodological quality (pg 5).<br>No automation tools used.
Effect measures	12	Specify for each outcome the effect measure(s) (e.g. risk ratio, mean difference) used in the synthesis or presentation of results.	Methods, Statistical analysis and data synthesis (pg 5)<br>Supplementary table 2
Synthesis methods	13a	Describe the processes used to decide which studies were eligible for each synthesis (e.g. tabulating the study intervention characteristics and comparing against the planned groups for each synthesis (item #5)).	Methods, Statistical analysis and data synthesis (pg 5)
	13b	Describe any methods required to prepare the data for presentation or synthesis, such as handling of missing summary statistics, or data conversions.	N/A Studies included providing 2x2 published data.
	13c	Describe any methods used to tabulate or visually display results of individual studies and syntheses.	Methods, Statistical analysis and data synthesis (pg 5)
	13d	Describe any methods used to synthesize results and provide a rationale for the choice(s). If meta-analysis was performed, describe the model(s), method(s) to identify the presence and extent of statistical heterogeneity, and software package(s) used.	Methods, meta-analysis (pg 5)
	13e	Describe any methods used to explore possible causes of heterogeneity among study results (e.g. subgroup analysis, meta-regression).	Methods, Investigating

For peer review only

PRISMA 2020 Checklist

Section and Topic	Item #	Checklist item	Location where item is reported
			heterogeneity (pg 5 & 6)
	13f	Describe any sensitivity analyses conducted to assess robustness of the synthesized results.	Results, Self-diagnosis of HIV (pg 9)
Reporting bias assessment	14	Describe any methods used to assess risk of bias due to missing results in a synthesis (arising from reporting biases).	Not examined.
Certainty assessment	15	Describe any methods used to assess certainty (or confidence) in the body of evidence for an outcome.	Methods, meta-analysis (pg 5)
**RESULTS**			
Study selection	16a	Describe the results of the search and selection process, from the number of records identified in the search to the number of studies included in the review, ideally using a flow diagram.	Results (pg 6) Figure 1
	16b	Cite studies that might appear to meet the inclusion criteria, but which were excluded, and explain why they were excluded.	N/A. Rather 2 studies were included that were borderline exclusion (pg 7 & 8)
Study characteristics	17	Cite each included study and present its characteristics.	Results (pg 6 – 9). Supplementary tables 4 & 5
Risk of bias in studies	18	Present assessments of risk of bias for each included study.	Figure 5
Results of individual studies	19	For all outcomes, present, for each study: (a) summary statistics for each group (where appropriate) and (b) an effect estimate and its precision (e.g. confidence/credible interval), ideally using structured tables or plots.	Figures 2 - 4
Results of syntheses	20a	For each synthesis, briefly summarise the characteristics and risk of bias among contributing studies.	Results (pg 6-9)
	20b	Present results of all statistical syntheses conducted. If meta-analysis was done, present for each the summary estimate and its precision (e.g. confidence/credible interval) and measures of statistical heterogeneity. If comparing groups, describe the direction of the effect.	Results, Self-diagnosis of HIV (pg 8 & 9). Figure 4
	20c	Present results of all investigations of possible causes of heterogeneity among study results.	Results, Self-diagnosis of HIV (pg 8 & 9). Figure 4
	20d	Present results of all sensitivity analyses conducted to assess the robustness of the synthesized results.	Results, Self-

For peer review only

PRISMA 2020 Checklist

Section and Topic	Item #	Checklist item	Location where item is reported
			diagnosis of HIV (pg 9).<br>Figure 4
Reporting biases	21	Present assessments of risk of bias due to missing results (arising from reporting biases) for each synthesis assessed.	N/A
Certainty of evidence	22	Present assessments of certainty (or confidence) in the body of evidence for each outcome assessed.	Discussion (pg 10 & 11)
**DISCUSSION**			
Discussion	23a	Provide a general interpretation of the results in the context of other evidence.	Discussion (pg 10 & 11)
	23b	Discuss any limitations of the evidence included in the review.	Discussion (pg 10 & 11)
	23c	Discuss any limitations of the review processes used.	Discussion (pg 10 & 11)
	23d	Discuss implications of the results for practice, policy, and future research.	Discussion (pg 11)
**OTHER INFORMATION**			
Registration and protocol	24a	Provide registration information for the review, including register name and registration number, or state that the review was not registered.	Abstract (pg 2).<br>Methods (pg 6)
	24b	Indicate where the review protocol can be accessed, or state that a protocol was not prepared.	Supplementary table 3
	24c	Describe and explain any amendments to information provided at registration or in the protocol.	Methods, meta-analysis (pg 5).<br>Results, self-diagnosis of HIV (post-hoc analysis, pg 9)
Support	25	Describe sources of financial or non-financial support for the review, and the role of the funders or sponsors in the review.	Funding statement (pg 11)
Competing interests	26	Declare any competing interests of review authors.	Competing interests statement (pg 11)
Availability of data, code and other materials	27	Report which of the following are publicly available and where they can be found: template data collection forms; data extracted from included studies; data used for all analyses; analytic code; any other materials used in the review.	Data availability statement (pg 11)

For peer review only

PRISMA 2020 Checklist

From: Page MJ, McKenzie JE, Bossuyt PM, Boutron I, Hoffmann TC, Mulrow CD, et al. The PRISMA 2020 statement: an updated guideline for reporting systematic reviews. BMJ 2021;372:n71. doi: 10.1136/bmj.n71
For more information, visit: http://www.prisma-statement.org/

For peer review only

BMJ Open

Accuracy of self-diagnosis in conditions commonly managed in primary care: Diagnostic accuracy systematic review and meta-analysis

Journal:	*BMJ Open*
Manuscript ID	bmjopen-2022-065748.R1
Article Type:	Original research
Date Submitted by the Author:	26-Sep-2022
Complete List of Authors:	Mclellan, Julie; University of Oxford Nuffield Department of Primary Care Health Sciences, Nuffield Department of Primary Health Care Sciences<br>Heneghan, Carl; University of Oxford Nuffield Department of Primary Care Health Sciences, Primary Health Care<br>Roberts, Nia; University of Oxford Bodleian Libraries, Bodleian Health Care Libraries,<br>Pluddemann, Annette; University of Oxford Nuffield Department of Primary Care Health Sciences, Nuffield Department of Primary Care Health Sciences
**Primary Subject Heading**:	General practice / Family practice
Secondary Subject Heading:	Dermatology, HIV/AIDS, Obstetrics and gynaecology
Keywords:	Adult dermatology < DERMATOLOGY, HIV & AIDS < INFECTIOUS DISEASES, PRIMARY CARE, GYNAECOLOGY

SCHOLARONE™
Manuscripts

I, the Submitting Author has the right to grant and does grant on behalf of all authors of the Work (as defined in the below author licence), an exclusive licence and/or a non-exclusive licence for contributions from authors who are: i) UK Crown employees; ii) where BMJ has agreed a CC-BY licence shall apply, and/or iii) in accordance with the terms applicable for US Federal Government officers or employees acting as part of their official duties; on a worldwide, perpetual, irrevocable, royalty-free basis to BMJ Publishing Group Ltd ("BMJ") its licensees and where the relevant Journal is co-owned by BMJ to the co-owners of the Journal, to publish the Work in this journal and any other BMJ products and to exploit all rights, as set out in our licence.

The Submitting Author accepts and understands that any supply made under these terms is made by BMJ to the Submitting Author unless you are acting as an employee on behalf of your employer or a postgraduate student of an affiliated institution which is paying any applicable article publishing charge ("APC") for Open Access articles. Where the Submitting Author wishes to make the Work available on an Open Access basis (and intends to pay the relevant APC), the terms of reuse of such Open Access shall be governed by a Creative Commons licence – details of these licences and which Creative Commons licence will apply to this Work are set out in our licence referred to above.

Other than as permitted in any relevant BMJ Author's Self Archiving Policies, I confirm this Work has not been accepted for publication elsewhere, is not being considered for publication elsewhere and does not duplicate material already published. I confirm all authors consent to publication of this Work and authorise the granting of this licence.

For peer review only

Accuracy of self-diagnosis in conditions commonly managed in primary care: Diagnostic accuracy systematic review and meta-analysis

Julie McLellan[1], Carl Heneghan[1], Nia Roberts[2], Annette Plüddemann[1]

[1] Nuffield Department of Primary Care Health Sciences, University of Oxford, OX2 6GG

[2] Bodleian Health Care Libraries, University of Oxford, Oxford, UK

Corresponding author: Annette Plüddemann, Nuffield Department of Primary Care Health Sciences, University of Oxford, OX2 6GG, annette.pluddemann@phc.ox.ac.uk, +44 (0)1865 289353

Keywords: Diagnostic accuracy systematic review, meta-analysis, self-diagnosis, common conditions in primary care,

Abstract word count: 299

Manuscript word count:

For peer review only

Abstract

Objectives:

To assess the diagnostic accuracy of self-diagnosis compared to a clinical diagnosis for common conditions in primary care

Design:

Systematic review. Meta-analysis

Data sources:

Medline, Embase, Cochrane CENTRAL, Cochrane CDSR, and CINAHL from inception to 25 January 2021

Study selection:

Eligible studies were prospective or retrospective studies comparing the results of self-diagnosis of common conditions in primary care to a relevant clinical diagnosis or laboratory reference standard test performed by a healthcare service provider. Studies that considered self-testing only were excluded.

Data extraction:

Two authors independently extracted data using a predefined data extraction form and assessed risk of bias using QUADAS-2.

Methods & Results:

5,047 records identified 18 studies for inclusion covering the self-diagnosis of three common conditions: vaginal infection (five studies), common skin conditions (four studies) and HIV (nine studies). No studies were found for any other condition. For self-diagnosis of vaginal infection and common skin conditions meta-analysis was not appropriate and data were reported narratively. Nine studies, using point-of-care oral fluid tests, reported on the accuracy of self-diagnosis of HIV and data were pooled using bivariate meta-analysis methods. For these nine studies the pooled sensitivity was 92.8% (95%CI, 86% to 96.5%) and specificity was 99.8% (95%CI, 99.1% to 99.9%). Post hoc, the robustness of the pooled findings was tested in a sensitivity analysis only including four studies using laboratory testing as the reference standard. The pooled sensitivity reduced to 87.7% (95%CI 81.4% to 92.2%) and the specificity remained the same. The quality of all 18 included studies was assessed as mixed and overall study methodology was not always well described.

Conclusions and implications of key findings:

Overall, there was a paucity of evidence. The current evidence does not support routine self-diagnosis for vaginal infections, common skin conditions and HIV in primary care.

PROSPERO registration number: CRD42018110288

For peer review only

Article Summary

Strengths and limitations of this study

- This systematic review summarises and interprets the available evidence on self-diagnosis of conditions managed in primary care
- This search strategy was extensive including publications identified from databases Medline, Embase, Cochrane Central Register of Controlled Trials (CENTRAL), Cochrane Database of Systematic Reviews (CDSR), and CINAHL, up to January 2021
- Standard methodology for systematic review of diagnostic accuracy studies was used, including study quality appraisal using QUADAS-2
- There was a paucity of evidence for many common conditions
- Lack of evidence meant meta-analysis was possible only for one condition

For peer review only

Introduction

The workload in primary care continues to increase (1, 2), in part due to global population increases, but also due to age of populations, change in lifestyle and more complex health problems (3). Increased workload has been recognised in the UK as an important factor in working conditions for primary care physicians (General Practitioners, GPs) and strategies are required to manage the workload (4).

One strategy is the potential for self-diagnosis and self-treatment by patients of some commonly occurring conditions. If feasible, this could lead to more rapid diagnosis and treatment reducing the burden on primary care services. The prospect of self-diagnosis is controversial with concerns if results are misinterpreted or patients fail to confirm their findings to a physician (5). In terms of the evidence, the first important question is to assess the diagnostic accuracy of self-diagnosis in the primary care setting. Subsequently, in order to support self-diagnosis, the efficacy needs to be assessed, to inform which conditions can self-diagnosed safely, in which circumstances, and by whom.

Cooke and colleagues recently reported the 30 most commonly managed conditions in primary care in Australia, which has a health landscape broadly comparable with western Europe (6). This list arises from survey data collected between January 2009 and December 2010, which included 194,100 patient encounters from 1,941 GPs.

This systematic review, therefore, aimed to assess the diagnostic accuracy of self-diagnosis compared to a clinical diagnosis for common conditions in primary care by a healthcare provider.

Methods

Types of studies

We included prospective or retrospective studies comparing the results of self-diagnosis of common conditions in primary care to the results of a relevant clinical diagnosis or laboratory reference standard test. We excluded studies with a case-control design due to their high risk of bias (4).

Population: Adults self-diagnosing common conditions in primary care. Common conditions included were broadly based on those reported by Cooke et al (6) and relevant for self-diagnosis (see online supplementary table 1). Studies in children, based in animals or non-human samples were excluded.

Index test: Self-diagnosis, where we defined “self-diagnosis” as a diagnosis made by the patients in the study, including self-evaluation and interpretation of results of rapid tests. Studies that considered self-testing only and not as part of self-diagnosis were excluded, in addition studies assessing accuracy of self-monitoring of an existing condition were excluded.

Reference standard: Clinical diagnosis or laboratory test performed by a healthcare service provider. We excluded studies comparing self-diagnosis with diagnosis by allied health professionals or pharmacists.

For peer review only - http://bmjopen.bmj.com/site/about/guidelines.xhtml

Outcome measures: Reported diagnostic accuracy measures (e.g. sensitivity, specificity, likelihood ratios, predictive values, etc.) and primary data for 2x2 tables. We excluded studies reporting only measures of agreement.

Search methods to identify studies

The search strategy was based on a combination of terms for self-testing and self-diagnosis, diagnostic accuracy terms (e.g. sensitivity, specificity, etc.) and terms for common conditions in primary care (6) (see online supplementary table 2 for full search strategy).

We searched the following electronic databases from inception to 25 January 2021: Medline (OvidSP)[1946-present], EMBASE (OvidSP)[1974-present], Cochrane Central Register of Controlled Trials (CENTRAL) and Cochrane Database of Systematic Reviews (CDSR) via Cochrane Library, Wiley)[Issue 1 of 12, January 2021] and CINAHL (EBSCOHost)[1982-present]. No restrictions were imposed on study population numbers or language (studies in languages other than English were translated). Letters, narrative reviews, and other non-primary sources were excluded. The reference lists of included studies, plus the first five "similar articles" identified through PubMed for these studies, and reference lists of relevant systematic reviews were used to identify further relevant publications. References were imported into Endnote X9 (7) where duplicates were removed.

Data collection and analysis

Selection of studies

Two reviewers independently applied the selection criteria to the titles and abstracts of the study reports identified by the searches. Full text of all studies that met the inclusion criteria were reviewed to agree the final list of included studies. Disagreements between reviewers were resolved by discussion and where agreement could not be reached a third reviewer was consulted.

Data extraction and management

Two reviewers (JM, AP) independently extracted information from selected studies into a pre-defined data extraction sheet (see online supplementary table 3) and crosschecked the data. Disagreements were resolved by discussion.

Assessment of methodological quality

We used the Quality Assessment of Diagnostic Accuracy Studies-2 (QUADAS-2) (8) tool to assess methodological quality of included studies. This considered the risk of bias in four domains (patient selection, index test, reference standard, flow and timing), as well as assessing the applicability (for the first three domains) of the studies to the review research question. Studies were assessed as low, high or unclear risk of bias/concerns regarding applicability for each domain. Two reviewers (JM, AP) independently assessed studies' methodological quality; disagreements were resolved by discussion, or if necessary, by a third reviewer. The results of the QUADAS-2 assessment were presented in a summary table.

Statistical analysis and data synthesis

Data were presented and analysed based on the condition being diagnosed. We compiled summary tables outlining the detailed study information of included studies, including the patient sample, condition, study design, setting, the test under evaluation, the comparator, and

For peer review only

conduct of the study. We extracted binary diagnostic accuracy data from all studies and constructed 2×2 tables.

Meta-analysis

We used Review Manager (9) to produce paired forest plots to explore the between-study variability of sensitivity and specificity across the included studies. For each study estimate of sensitivity and specificity, corresponding 95% confidence intervals were shown to illustrate the uncertainty related to each study estimate. Where different thresholds were applied these were reported. Where appropriate, we used bivariate meta-analysis methods (10) to generate pooled estimates of sensitivity and specificity. Due to the nature of the data a change was made to the protocol and RStudio (11) was used to generate the model parameters to input into Revman (9).

Investigating heterogeneity

For medical conditions for which data from more than one study was available, and where it was possible to investigate between-study heterogeneity in the results, inclusion of study level characteristics as covariates in meta-analysis and subgroup analyses were considered. These approaches were carried out if there was sufficient data available and sub-group specific pooled estimates were thought to be of clinical relevance.

Investigating reporting bias

Funnel plots used to detect publication bias in reviews of RCTs have been shown to be misleading for diagnostic test accuracy reviews (12, 13). Funnel plots as an assessment of reporting bias were therefore not be included in this review.

Patient involvement

Members of the public were part of the Research Programme Committee of the National Institute for Health Research (NIHR) programme grant that funded this study. Updates and details about the study were presented to the committee while the study was ongoing, and the public members provided feedback. This review formed part of the NIHR Evidence Synthesis Working Group (ESWG) and members of the public who were part of the ESWG steering committee commented on the protocol for the study and on updates presented to the steering committee.

The full protocol is provided as online supplementary table 4 and is registered on PROSPERO reference number CRD42018110288.

Results

Figure 1 shows a summary of the search results and the inclusion and exclusion of studies. After removal of duplicates, 5,047 records were identified through database searches, websites and citation searching. This resulted in full texts of 170 articles being assessed for eligibility, and 20 included articles (14-33) reporting results of 18 individual studies (14-20, 22-28, 30-33). These 18 included studies fell into 3 broad groups of commonly managed conditions: Female genital infection, dermatitis, contact/allergic and viral disease, not otherwise specified. No studies of self-diagnosis were found for any other conditions in primary care.

For peer review only

Most excluded studies only reported on patients' ability to self-test (or self-monitor an existing condition) with the diagnosis being made by a clinician, and did not report diagnostic accuracy of self-diagnosis.

Of the included studies, five reported on the accuracy of self-diagnosis of vaginal infection (11-15), four for common skin conditions (19, 20, 22, 23) and nine for the self-diagnosis for HIV (24-28, 30-33). Online supplementary tables 5 and 6 summarise the characteristics of included studies and characteristics of self-diagnostic (index) and reference tests, respectively. Paired plots of sensitivity and specificity were generated, grouping the studies by the condition to be diagnosed (Figures 2-4). For studies examining the accuracy of self-diagnosis of vaginal infection and in common skin conditions (14-20, 22, 23), meta-analysis was not appropriate due to the between-study heterogeneity and the overall low number of studies, which would make meta-analysis uninformative (34).

Self-diagnosis of vaginal infections

Five studies assessed the accuracy of self-diagnosis of Bacterial vaginosis and/or infection with *Trichomonas vaginalis (14-18),* with one study assessing the self-diagnosis of *Candida* vaginitis (17) (Figure 2). For Bacterial vaginosis, the accuracy of a vaginal fluid test using a pH strip (for Bacterial vaginosis) was assessed with laboratory testing (Gram staining) as the reference standard in two studies (14, 15) (online supplementary table 5). For the diagnosis of Bacterial vaginosis and/or *T. vaginalis*, a panty liner test kit (VI-SENSE) for vaginal discharge was assessed against a combination of clinical and laboratory assessment as the reference test in one study (16); and a vaginal fluid self-diagnosis kit for women in the military was assessed with clinical and laboratory assessment as the reference test in the second study (17). One study used a vaginal fluid dipstick test for the presence of *T. vaginalis* (OSOM *Trichomonas* rapid test) compared to a laboratory PCR as a reference test (18). For the self-diagnosis of *Candida* vaginosis a military self-testing kit based on a combination of the measurement of pH, amines and the symptom of vaginal itching for self-diagnosis was compared to a combination of clinical and microbiological laboratory assessment in one study (17).

Bacterial vaginosis; Bacterial vaginosis and/or Trichomonas vaginalis

The sensitivity of a self-taken swab applied to a pH test strip for self-diagnosis of Bacterial vaginosis ranged from 0.45 (95%CI, 0.34-0.56) (14) to 0.60 (95%CI, 0.55-0.66) (17) at a pH cut-off of ≥ 4.7. Notably, Ryan-Wenger et al (17), the study reporting a sensitivity of 0.60, also included symptoms (vaginal itching) and the presence of amines as part of the assessment, which may explain the higher sensitivity. Donders et al (14) assessed a lower cut-off of pH ≥ 4.5 and showed an increased sensitivity of 0.95 (95%CI, 0.88-0.99); however at the expense of specificity. The study on pregnant women (15) assessing the accuracy of pH test strips did not specify the pH cut-off and reported a sensitivity of 0.36 (95%CI, 0.17-0.59) with a specificity of 0.88 (95%CI, 0.82-0.93). The specificity for the pH test strip tests ranged from 0.5 (95%CI, 0.43-0.56) (17) to 0.81 (95%CI, 0.75-0.85) (14) at the pH cut-off of ≥ 4.7, decreasing to 0.41 (95%CI, 0.34-0.47) at the lower cut-off of pH ≥ 4.5 (14). Interestingly the low specificity of 0.5 was reported by the study combining the pH test strip with symptoms and the presence of amines (17).

The study assessing the vaginal discharge test using a panty liner test kit with an indicator strip incorporated in the liner (16) reported a sensitivity of 0.91 (95%CI, 0.86-0.94) and specificity of 0.81 (95%CI, 0.76-0.86) for the diagnosis of Bacterial vaginosis and/or *T. vaginalis* infection.

For peer review only

Trichomonas vaginalis

One study in Brazil assessed a rapid immunochromatographic *T. vaginalis* test for use at home (18) and reported a sensitivity of 0.68 (95%CI, 0.43-0.87) and specificity of 1.00 (95%CI, 0.99-1.00) for self-diagnosis of *T. vaginalis* infection.

Candida vaginitis

Only one study (17) specifically assessed the diagnostic accuracy of self-diagnosis of *Candida* vaginitis, which formed part of the military self-testing kit, and reported a sensitivity of 0.18 (95%CI, 0.12-0.25) and specificity of 0.89 (95%CI, 0.85-0.92).

Self-diagnosis of common skin conditions

Four studies assessed the accuracy of self-diagnosis of common skin conditions (19, 20, 22, 23) (Figure 3). We included 2 studies that were outside our age inclusion criteria: Berghoi 2011 reported patients included had a mean age 17.5 years old, nevertheless these patients would have been 16+ years to qualify as apprentice hairdressers. And Svensson 2002 reported the mean age of patients as 40.4 years (no standard deviation), but included patients from age 16.

Eczema

Two studies assessed the accuracy of self-diagnosis based on a self-evaluated questionnaire of signs and symptoms for the diagnosis of eczema alongside a self-assessment of the presence or absence of eczema based on the questionnaire results, compared to assessment by a clinician (19, 20). Overall, 710 participants were included across the two studies. The reported sensitivity ranged from 0.7 (95%CI, 0.53- 0.84) to 0.87 (95%CI, 0.78-0.93) and specificity ranged from 0.79 (95%CI, 0.70-0.86) to 1.00 (95%CI, 0.99-1.00). The relatively high specificity suggests the potential for patients to use the questionnaire as a tool to confirm that they have eczema and therefore seek healthcare advice; however, it should be noted that there was an unclear risk of bias regarding the patient selection. In Svensson et al (20), the patients were recruited at a dermatology outpatient clinic, where they had been referred to and 113 patients in the study reported having had a diagnosis of eczema in the last 12 months, suggesting a more selected population with a higher pre-test probability. Whilst the setting in the study by Bregnhoj et al. (19) was not reported, it was conducted amongst hairdressers who may have had more experience of eczema either themselves or colleagues being diagnosed with the condition, given the nature of their profession. They may be more aware of the signs and symptoms and may constitute a selected population. Therefore, the diagnostic accuracy of the questionnaire for self-diagnosis may be dependent on the type of population.

Skin allergy

Two studies assessed the diagnostic accuracy of self-diagnosis to detect an allergic skin reaction including 408 participants across the two studies; one study assessed nickel and/or fragrance allergy (22), and the other nickel allergy alone (23). Both studies used a patch test applied to the arm, which as self-evaluated by participants 2-4 days later. Dermatologists then also evaluated the patch tests as the reference standard. One study recruited participants at hospital dermatology departments (23), while the other recruited through a newspaper advertisement targeted at people with a self-suspected allergy towards fragrance and/or nickel

For peer review only

(22). Sensitivity ranged from 0.72 (95%CI, 0.57-0.84) to 0.97 (95%CI. 0.87-1.00) and specificity ranged from 0.87 (95%CI, 0.80-0.92) to 0.91 (95%CI, 0.85-0.95). Elsner et al. (22) also reported that participants found the information regarding how to apply the test extensive and detailed, the information regarding self-evaluation of the test was limited and should be improved.

Self-diagnosis of HIV

Nine studies were identified that reported the diagnostic accuracy of self-testing and self-diagnosis of HIV (24-28, 30-33). In all studies self-diagnosis was undertaken unsupervised using a rapid point of care (POC) oral fluid test manufactured by OraSure technologies, either OraQuick Inhome intended for lay users or OraQuick Advance intended for professional use. The studies recruited 13103 participants, and all were conducted in African countries except for the phase III trial in the USA by OraSure Technologies (33). The 2019 global HIV prevalence rates for women and men aged 15 to 49 were 0.8% (95%CI, 0.7–1.0) and 0.6% (95%CI, 0.5-0.8) respectively with the overall highest prevalence by country in Eswatini (Africa) at 27.1% (95%CI, 25.4-28.8] (35). All included studies had prevalence rates above the global averages for men and women or, if not reported, were in countries with high prevalence rates. Prevalence rates ranged from 2.12% in the USA study (33) to 22.1% in the Ugandan study (24). The USA study was conducted in 20 clinical sites, 17 identified as high prevalence sites (2.6%) and 3 as low prevalence sites (0.1%). All studies enrolled participants from the general population including the USA where no breakdown of sexual orientation was reported. The reported sensitivity and specificity were similar between studies (Figure 4a); the single study (32) reporting a lower estimate for sensitivity still had a confidence interval that overlapped with half of the other studies. The pooled sensitivity based on all 9 included studies was 92.8% (95%CI, 86% to 96.5%) and the pooled specificity was 99.8% (95%CI, 99.1% to 99.9%). The studies showed low heterogeneity (Figure 4b). The reference standard used in the studies was one of three types: four studies took a venous sample which was sent to a laboratory for testing (28, 30, 32, 33), two studies used a nationally approved algorithm based on a combination of rapid POC tests (24, 25) and three studies used a study based algorithm again based on rapid POC tests (26, 27, 31). In three studies using POC tests for the reference standard, the diagnosis may have been by clinicians, but it was unclear. These studies reported the diagnosis by a research assistant (24) or a counsellor (26, 31). Post hoc, a sensitivity analysis was conducted to test the robustness of the pooled findings by removing the studies using POC tests as the reference standard (including tests where it was unclear whether diagnosis was by a clinician) and only including those studies using laboratory testing. Based on four studies using laboratory testing as the reference standard (28, 30, 32, 33), the pooled sensitivity was 87.7% (95%CI 81.4% to 92.2%) and the pooled specificity was 99.8% (95%CI 98.9% to 99.9%). No data were reported by participant characteristics such as gender, ethnicity, or sexual orientation.

A number of studies (24-26, 30-32) reported on the viability or feasibility of the oral fluid self-test stating that participants found it easy to conduct, but acknowledged that instructions should be adapted to the population using the test, particularly the literacy levels. Furthermore, users must be encouraged to receive a confirmatory test.

Methodological quality of included studies

For peer review only

Assessment of the quality of included studies using the QUADAS-2 framework (8) is presented in Figure 5, which summarises the overall risk of bias and applicability concerns. For patient selection, the risk of bias overall was mixed; where studies were rated unclear in this domain it was because several studies either recruited a selected population with a potentially higher pre-test probability or they did not clearly report the recruitment strategy and whether eligible patients were consecutively recruited. However, with the exception of one study, applicability concerns were low. In several cases, although the population may have been a selected population (e.g. at high risk of HIV infection or skin eczema; or with a prior history of eczema or vaginal infection), it could be argued that these might be the populations where self-diagnosis and/or self-testing may be most relevant. Overall risk of bias regarding the conduct of the index test was low and in most studies participants were blinded to the results of the reference test, though in some studies this was not clearly described. Risk of bias with respect to the reference test was unclear or high in some studies as assessors were either not blinded to the results of the index test or blinding was unclear. For the domain of flow and timing, several studies were judged to be at high risk of bias as the interval between testing was frequently not explicitly reported. In addition differential reference bias was identified as present in some studies and unclear in others, in particular it was unclear in several studies how clinical assessment was conducted or standardised. Overall study procedures were not always clearly described.

Discussion

With the increasing workload in primary care (1) and the continued development of rapid tests, including those that are intended to be used by patients, we aimed to assess the evidence for the diagnostic accuracy of self-diagnosis. We identified limited evidence on the diagnostic accuracy of self-diagnosis: only 20 publications (reporting data from 18 studies) specifically assessed the accuracy of self-diagnosis, covering three commonly managed conditions, namely vaginal infections, common skin conditions and HIV. Interestingly, no studies of self-diagnosis were found for any other conditions in primary care. It was particularly notable that we did not find any studies assessing the diagnostic accuracy of self-diagnosis of common primary conditions such as upper respiratory tract and urinary tract infections. As technology develops potentially enabling increased self-diagnosis in primary care, we would expect future reviews examining this research question to include more common conditions. In particular, we would expect to include studies for self-diagnosis of COVID-19 following the rapid development of tests during the COVID-19 pandemic.

The evidence for self-diagnosis of vaginal infection suggests a lack of sufficient accuracy to aid self-diagnosis. Tests relying on self-swabs and pH strips showed low sensitivity (below 60%), and therefore would not be useful to rule out disease. Although sensitivity improved to 95% with increasing pH cut-off, this occurred at the cost of specificity, which dropped from 81% to 41%. Using the test at this cut-off would, therefore, result in concern for missed diagnoses. The immunochromatography test also showed insufficient sensitivity (68%) to be of use as a rule-out test. The panty liner test, however, had high sensitivity of 91% and may prove to be a useful rule-out test, although this result is limited as it is based on one study judged to be at unclear risk of bias, with a high risk of bias in the flow and timing domain. In terms of their use to confirm the presence of vaginal infection, the highest specificity (100%) was reported for the immunochromatographic test. This may be a useful test to aid self-diagnosis of vaginal infection in systems that currently rely on syndromic management, such as low resource settings, particularly to improve the targeting of antimicrobial prescribing.

For peer review only

However, it should be noted this result is also based on one study and requires confirming in a larger study.

The allergy patch tests overall showed reasonable accuracy (sensitivity 72-97%; specificity 87-91%) and may be useful as an initial self-screening test for patients with a suspected nickel or fragrance allergy; however it could be argued that the main use of these tests might be to safely rule out a contact allergy. The reported diagnostic accuracy suggests the tests are not sufficiently sensitive as a rule-out test, particularly given the relatively wide confidence intervals. The tests may however be useful in settings where access to dermatology services is scarce. The self-diagnosis of eczema using a questionnaire of signs and symptoms showed specificity ranging from 0.79 to 1.00, suggesting this test might be useful as a confirmation test for patients for suspect they have eczema and to then seek treatment and management advice. The selected nature of the patients in the studies (i.e. patients with a previous eczema diagnosis and hairdressers, who may encounter eczema more frequently due to their profession) may overestimate the accuracy of the test; however, it could be argued that these might be the populations in whom the test is most relevant.

We identified nine studies that reported the accuracy of self-diagnosis of HIV (sensitivity 93%, specificity 99%). However, the sensitivity is reduced to 88% when only studies using a venous sample and laboratory testing as the reference standard are included. With a sensitivity of 88%, the accuracy data would not support the use of this test as a rule-out test, particularly given the clinical consequences of a false negative test result. Evidence suggests there may be benefits to self-initiated HIV testing, including early identification, increased likelihood for the uptake of HIV prevention interventions, and a reduction in sexual risk behaviours (36), warranting further research, in particular in resource-limited settings where access to testing sites may be a barrier. However, HIV self-testing should also be considered in the context of linkage to care, access to counselling, and adequate regulatory and quality assurance systems (37).

The search strategy for this review was broad and extensive with few restrictions resulting in the high number of publications to screen. Whilst it is possible, it is unlikely, studies were missed. We are unaware of any other reviews examining this research question. The main limitation was the lack of available evidence for a number of common conditions; studies reporting on self-testing alone were more common, but few studies assessed self-diagnosis, with patients interpreting the test results and making a diagnosis independently. For the three conditions where we identified studies reporting on the diagnostic accuracy of self-diagnosis, there was a paucity of evidence. Many studies were not replicated and included small sample sizes and contained methodological biases that limited the application of the results to practice. For self-diagnosis of vaginal infections, common skin conditions, and HIV further research is required to draw a definitive conclusion on the efficacy of self-diagnosis. For other common conditions in primary care, research is needed on self-diagnosis where this option is available, and studies should go beyond considering self-testing alone and also assess the diagnostic accuracy of self-diagnosis. Terminology for self-diagnosis, self-testing and self-screening is overlapping in some cases and needs clarifying. Finally, research is required into the patient's readiness and attitude towards self-diagnosis along with its effect on the patient/physician relationship.

The current limited evidence does not support routine self-diagnosis for vaginal infections, common skin conditions and HIV in primary care.

For peer review only

Acknowledgements

The authors would like to thank Hayley Jones for joint authorship of the original protocol, Elizabeth Spencer for assistance with preparation of the protocol, and Emily McFadden for assistance with screening of studies for inclusion.

Contributors

AP and CH conceived the idea for this review. AP led the research, developed the protocol, and conducted the review (screening and extraction). CH developed the protocol and provided clinical input. NR devised and conducted the search strategy, and approved the manuscript. JM conducted the review (screening, extraction, data analysis). JM, AP and CH jointly prepared the manuscript.

Funding

This project was funded by National Institute for Health Research School for Primary Care Research (NIHR SPCR) [Project Number 390]

Data availability statement

All data are available in published articles.

Competing interests

Julie McLellan reports grants from NIHR School of Primary Care Research (Evidence Synthesis Working Group Project Number 390) and part-funding from the Thames Valley Applied Research Collaborative (ARC), during the conduct of the study; and occasionally receives expenses for teaching Evidence-Based Medicine

Dr. Plüddemann reports grants from NIHR School of Primary Care Research (Evidence Synthesis Working Group Project Number 390), during the conduct of the study; and occasionally receives expenses for teaching Evidence-Based Medicine.

Dr. Heneghan reports receiving expenses and fees for his media work. He has received expenses from the WHO and holds grant funding from the NIHR, the NIHR School of Primary Care Research, The Wellcome Trust and the WHO. He has received financial remuneration from an asbestos case. He has also received income from the publication of a series of toolkit books published by Blackwells. On occasion, he receives expenses for teaching EBM and is also paid for his GP work in NHS out of hours. CEBM jointly runs the EvidenceLive Conference with the BMJ and the Overdiagnosis Conference with some international partners which are based on a non-profit making model.

References

1. Hobbs FDR, Bankhead C, Mukhtar T, Stevens S, Perera-Salazar R, Holt T, et al. Clinical workload in UK primary care: a retrospective analysis of 100 million consultations in England, 2007-14. Lancet. 2016;387(10035):2323-30.
2. Irving G, Neves AL, Dambha-Miller H, Oishi A, Tagashira H, Verho A, et al. International variations in primary care physician consultation time: a systematic review of 67 countries. BMJ Open. 2017;7(10):e017902.

For peer review only

3. Orgnaistion WH. Global status report on noncommunicable diseases 2014. 2014.
4. British Medical Association. BMA guidance: Controlling workload in general practice strategy 2020 [Available from: https://www.bma.org.uk/advice-and-support/gp-practices/managing-workload/controlling-workload-in-general-practice-strategy] Accessed 18 June 2021.
5. Hynes V. The trend toward self-diagnosis. CMAJ. 2013;185(3):E149-E50.
6. Cooke G, Valenti L, Glasziou P, Britt H. Common general practice presentations and publication frequency. Australian Family Physician. 2013;42:65-8.
7. The EndNote Team. Endnote. Endnote X9 ed. Philadelphia, PA: Clarivate; 2013.
8. Whiting PF, Rutjes AW, Westwood ME, Mallett S, Deeks JJ, Reitsma JB, et al. QUADAS-2: a revised tool for the quality assessment of diagnostic accuracy studies. Ann Intern Med. 2011;155(8):529-36.
9. Review Manager (RevMan) [Computer program]. Version 5.4. The Cochrane Collaboration. 2020.
10. Reitsma JB, Glas AS, Rutjes AW, Scholten RJ, Bossuyt PM, Zwinderman AH. Bivariate analysis of sensitivity and specificity produces informative summary measures in diagnostic reviews. J Clin Epidemiol. 2005;58(10):982-90.
11. RStudio Team. 2021. RStudio: Integrated Development Environment for R. RStudio, PBC, Boston, MA [Available from: http://www.rstudio.com/] Accesssed 22 June 2021.
12. Deeks JJ, Macaskill P, Irwig L. The performance of tests of publication bias and other sample size effects in systematic reviews of diagnostic test accuracy was assessed. J Clin Epidemiol. 2005;58(9):882-93.
13. Leeflang MM, Deeks JJ, Gatsonis C, Bossuyt PM. Systematic reviews of diagnostic test accuracy. Ann Intern Med. 2008;149(12):889-97.
14. Donders GGG, Gonzaga A, Marconi C, Donders F, Michiels T, Eggermont N, et al. Increased vaginal pH in Ugandan women: what does it indicate? European Journal of Clinical Microbiology & Infectious Diseases. 2016;35(8):1297-303.
15. Sungkar A, Purwosunu Y, Aziz MF, Pratomo H, Sutrisna B, Sekizawa A. Influence of early self-diagnosis and treatment of bacterial vaginosis on preterm birth rate. International Journal of Gynaecology & Obstetrics. 2012;117(3):264-7.
16. Geva A, Bornstein J, Dan M, Shoham HK, Sobel JD. The VI-SENSE-vaginal discharge self-test to facilitate management of vaginal symptoms. American Journal of Obstetrics & Gynecology. 2006;195(5):1351-6.
17. Ryan-Wenger NA, Neal JL, Jones AS, Lowe NK. Accuracy of vaginal symptom self-diagnosis algorithms for deployed military women. Nursing Research. 2010;59(1):2-10.
18. Jones HE, Lippman SA, Caiaffa-Filho HH, Young T, van de Wijgert JHHM. Performance of a rapid self-test for detection of Trichomonas vaginalis in South Africa and Brazil. Journal of Clinical Microbiology. 2013;51(3):1037-9.
19. Bregnhoj A, Sosted H, Menne T, Johansen JD. Validation of self-reporting of hand eczema among Danish hairdressing apprentices. Contact Dermatitis. 2011;65(3):146-50.
20. Svensson A, Lindberg M, Meding B, Sundberg K, Stenberg B. Self-reported hand eczema: symptom-based reports do not increase the validity of diagnosis. British Journal of Dermatology. 2002;147(2):281-4.
21. Elsner P, Bjarnoe H, Aberer W, Brasch J. Evaluation of a self-test device in allergic contact dermatitis. Dermatitis. 2009;20 (4):230-1.
22. Elsner P, Bjarnøe H, Aberer W, Brasch J. Evaluation of a Self-Test Device Used in Allergic Contact Dermatitis. Skin Pharmacol Physiol. 2015;28(5):257-63.
23. Josefson A, Svensson A, Farm G, Engfeldt M, Meding B. Validation of self-testing as a method to estimate the prevalence of nickel allergy. Acta Dermato-Venereologica. 2011;91(5):526-30.

For peer review only

24. Asiimwe S, Oloya J, Song X, Whalen CC. Accuracy of un-supervised versus provider-supervised self-administered HIV testing in Uganda: a randomized implementation trial. AIDS and behavior. 2014;18(12):2477-84.
25. Belete W, Deressa T, Feleke A, Menna T, Moshago T, Abdella S, et al. Evaluation of diagnostic performance of non-invasive HIV self-testing kit using oral fluid in Addis Ababa, Ethiopia: A facility-based cross-sectional study. PLoS One. 2019;14(1):e0210866.
26. Choko AT, Desmond N, Webb EL, Chavula K, Napierala-Mavedzenge S, Gaydos CA, et al. The uptake and accuracy of oral kits for HIV self-testing in high HIV prevalence setting: a cross-sectional feasibility study in Blantyre, Malawi. Plos medicine. 2011;8(10):e1001102.
27. Choko AT, MacPherson P, Webb EL, Willey BA, Feasy H, Sambakunsi R, et al. Uptake, Accuracy, Safety, and Linkage into Care over Two Years of Promoting Annual Self-Testing for HIV in Blantyre, Malawi: A Community-Based Prospective Study. PLoS Med. 2015;12(9):e1001873.
28. Kapaku K, Newman M, Maluzi K, Sigande L, Taegtmeyer M, Corbett E, et al., editors. Is OraQuick HIV-self-testing valid among intended users? Analysis from a clinical performance study in
Lusaka, Zambia. 22nd International AIDS Conference; 2017 24-26 July; Paris, France.
29. UNITAID/PSI HIV Self-Testing Africa (HIV STAR) study. A clinical performance study of self-testing using self-collected oral fluid transudate and the OraQuick® HIV Self-Test [Available from: https://hivstar.lshtm.ac.uk/files/2016/11/STAR-Protocol-for-Zambia-Clinical-Performance-Study-web.pdf] Accessed 18 June 2021.
30. Kurth AE, Cleland CM, Chhun N, Sidle JE, Were E, Naanyu V, et al. Accuracy and Acceptability of Oral Fluid HIV Self-Testing in a General Adult Population in Kenya. AIDS Behav. 2016;20(4):870-9.
31. Martínez Pérez G, Steele SJ, Govender I, Arellano G, Mkwamba A, Hadebe M, et al. Supervised oral HIV self-testing is accurate in rural KwaZulu-Natal, South Africa. Trop Med Int Health. 2016;21(6):759-67.
32. Pant Pai N, Behlim T, Abrahams L, Vadnais C, Shivkumar S, Pillay S, et al. Will an unsupervised self-testing strategy for HIV work in health care workers of South Africa? A cross sectional pilot feasibility study. PLoS One. 2013;8(11):e79772.
33. OraSure Technologies. Final Advisory Committee Briefing Materials: Available for Public Release. OraQuick In-Home HIV Test. Washington (D.C.): Food and Drug Administration: Blood Products Advisory Committee; 2012.
34. Macaskill P, Gatsonis C, Deeks JJ, Harbord RM, Takwoingi Y. Cochrane Handbook for Systematic Reviews of Diagnostic Test Accuracy.: The Cochrane Collaboration; http://srdta.cochrane.org; 2010.
35. UNAIDS. AIDSinfo Global factsheets 2019. HIV and AIDS Estimates [Available from http://aidsinfo.unaids.org/] Accessed 22 June 2021.
36. Adebayo OW, Salerno JP. Facilitators, Barriers, and Outcomes of Self-Initiated HIV Testing: An Integrative Literature Review. Res Theory Nurs Pract. 2019;33(3):275-91.
37. Njau B, Covin C, Lisasi E, Damian D, Mushi D, Boulle A, et al. A systematic review of qualitative evidence on factors enabling and deterring uptake of HIV self-testing in Africa. BMC Public Health. 2019;19(1):1289.

For peer review only

Figure 1: Study Selection

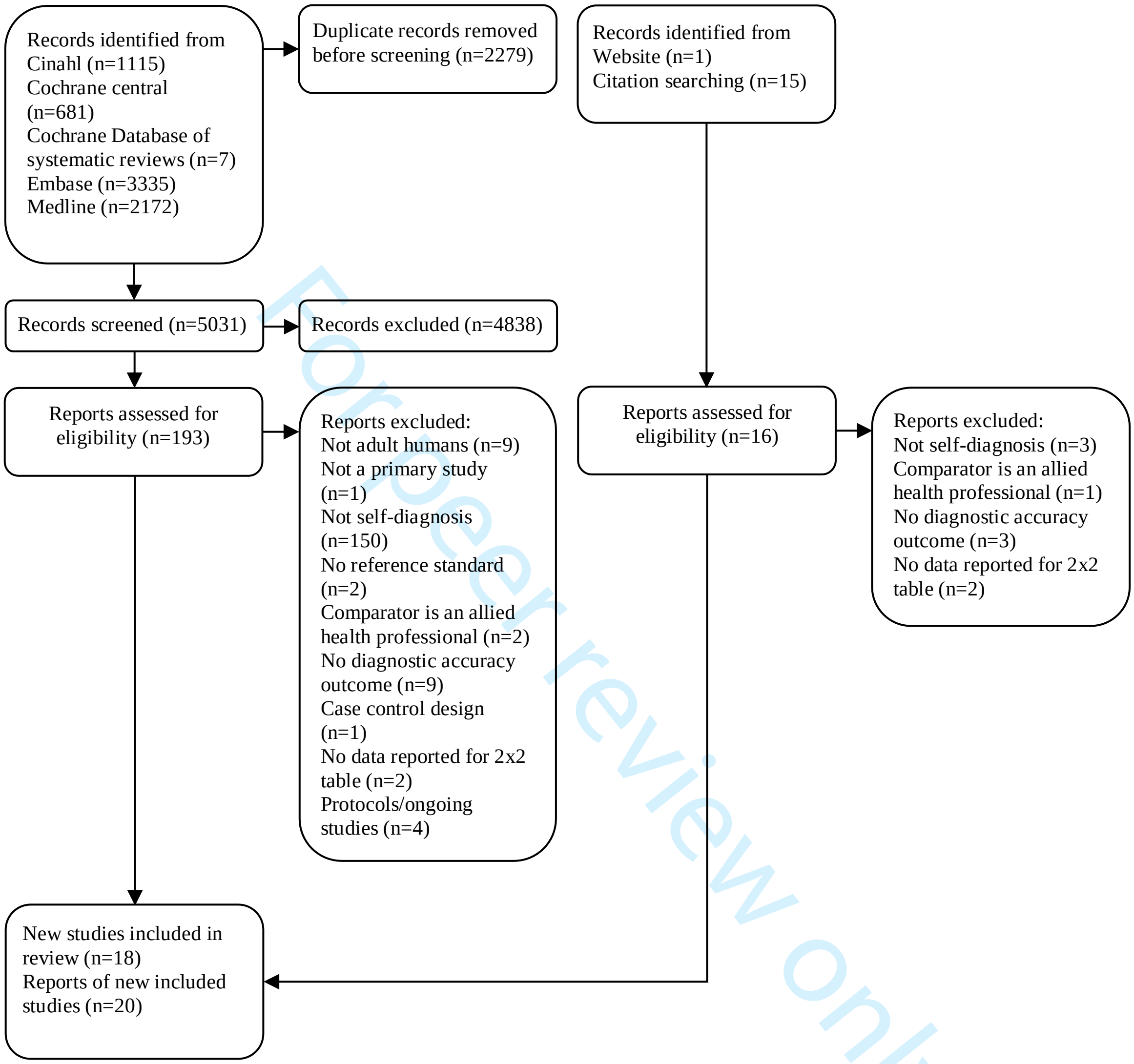

For peer review only

Figure 2: Paired forest plots of sensistivity and specificity for studies of self-diagnosis of vaginal infection (where Donders 2016a used pH threshold ≥ 4.5, Donders 2016b used pH threshold ≥ 4.7. Sungkar 2012 represents pooled data from 4 time points)

Study	TP	FP	FN	TN	Sensitivity (95% CI)	Specificity (95% CI)
**Bacterial vaginosis**						
Donders 2016a	80	151	4	103	0.95 [0.88, 0.99]	0.41 [0.34, 0.47]
Donders 2016b	38	49	46	205	0.45 [0.34, 0.56]	0.81 [0.75, 0.85]
Sungkar 2012	8	16	14	122	0.36 [0.17, 0.59]	0.88 [0.82, 0.93]
**Bacterial vaginosis &/or Trichomonas vaginalis**						
Geva 2006	226	50	23	217	0.91 [0.86, 0.94]	0.81 [0.76, 0.86]
Ryan-Wenger 2010	193	109	127	107	0.60 [0.55, 0.66]	0.50 [0.43, 0.56]
**Candida vaginitis**						
Ryan-Wenger 2010	26	43	122	345	0.18 [0.12, 0.25]	0.89 [0.85, 0.92]
**Trichomonas vaginalis**						
Jones 2013	13	1	6	675	0.68 [0.43, 0.87]	1.00 [0.99, 1.00]

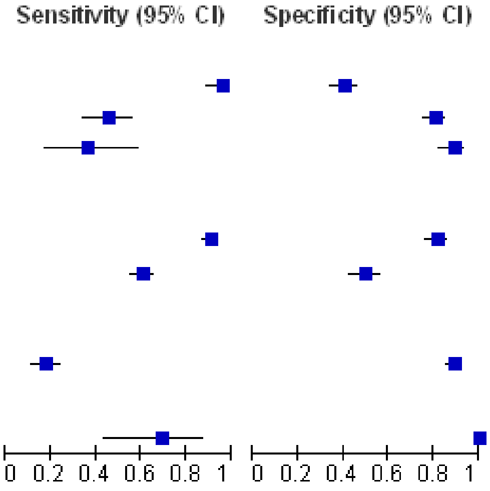

For peer review only

Figure 3: Paired forest plots of sensistivity and specificity for studies of self-diagnosis of common skin conditions (where Bregnhoj 2011 represents pooled data from recruitment and follow up time points)

Study	TP	FP	FN	TN	Sensitivity (95% CI)	Specificity (95% CI)
**Eczema**						
Bregnhoj 2011	26	1	11	726	0.70 [0.53, 0.84]	1.00 [0.99, 1.00]
Svensson 2002	78	22	12	82	0.87 [0.78, 0.93]	0.79 [0.70, 0.86]
**Nickel & fragrance allergy**						
Elsner 2015	39	16	1	106	0.97 [0.87, 1.00]	0.87 [0.80, 0.92]
**Nickel allergy**						
Josefson 2011	33	13	13	132	0.72 [0.57, 0.84]	0.91 [0.85, 0.95]

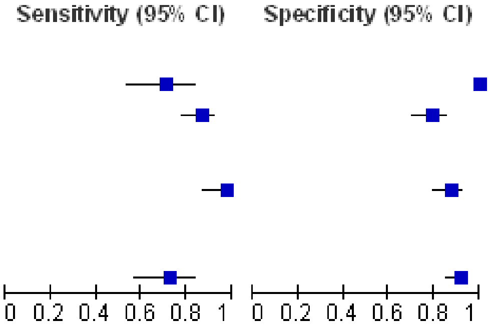

For peer review only

<u>Figure 4: Studies of self-diagnosis of HIV</u>

(a) Paired forest plots of sensitivity and specificity (where Choko 2015 represents pooled data from 1- 12 and 13 – 24 months follow up time points)

Study	TP	FP	FN	TN	Sensitivity (95% CI)	Specificity (95% CI)
Assiimwe 2014	24	5	3	91	0.89 [0.71, 0.98]	0.95 [0.88, 0.98]
Belete 2019	199	0	1	200	0.99 [0.97, 1.00]	1.00 [0.98, 1.00]
Choko 2011	27	0	1	210	0.96 [0.82, 1.00]	1.00 [0.98, 1.00]
Choko 2015	132	1	9	1507	0.94 [0.88, 0.97]	1.00 [1.00, 1.00]
Kapaku 2017	227	7	32	2286	0.88 [0.83, 0.91]	1.00 [0.99, 1.00]
Kurth 2016	26	1	3	173	0.90 [0.73, 0.98]	0.99 [0.97, 1.00]
Martinez Perez 2016	323	0	4	1860	0.99 [0.97, 1.00]	1.00 [1.00, 1.00]
Orasure 2012	106	1	8	5385	0.93 [0.87, 0.97]	1.00 [1.00, 1.00]
Pant Pai 2013	6	0	3	242	0.67 [0.30, 0.93]	1.00 [0.98, 1.00]

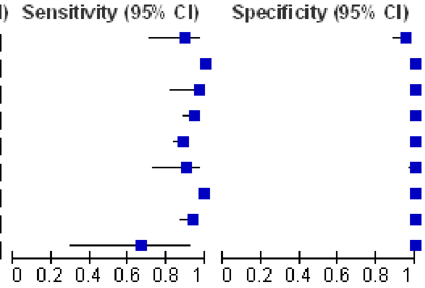

(b) Receiver operating characteristic plot HIV self-diagnosis compared with clinical diagnosis or laboratory reference test grouped by reference test type (where size of symbol indicates study size)

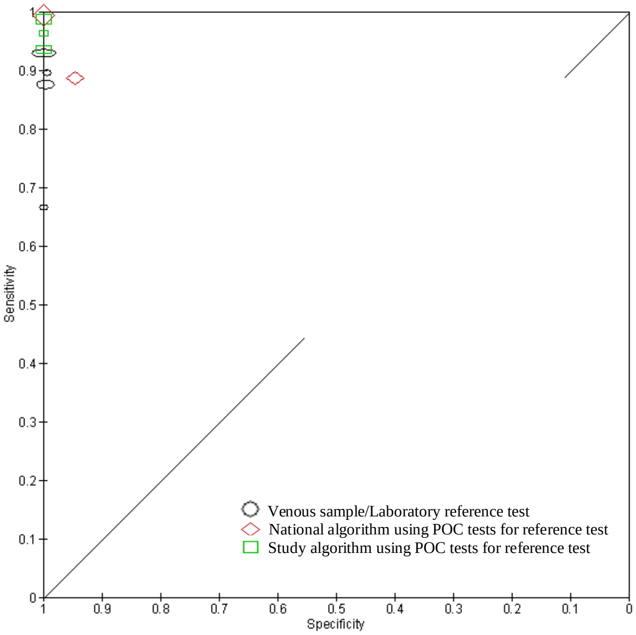

All studies (n=9):
Sensitivity: 92.8% (95%CI 86% to 96.5%); Specificity: 99.8% (95%CI 99.1% to 99.9%)
Sensitivity analysis retaining only studies with venous/laboratory reference test (n=4):
Sensitivity: 87.7% (95%CI 81.4% to 92.2%); Specificity: 99.8% (95%CI 98.9% to 99.9%)

Figure 5: QUADAS-2 summary of risk of bias and applicability concerns showing review authors' judgments about each domain for each included study (based on 19 studies (20 data sets))

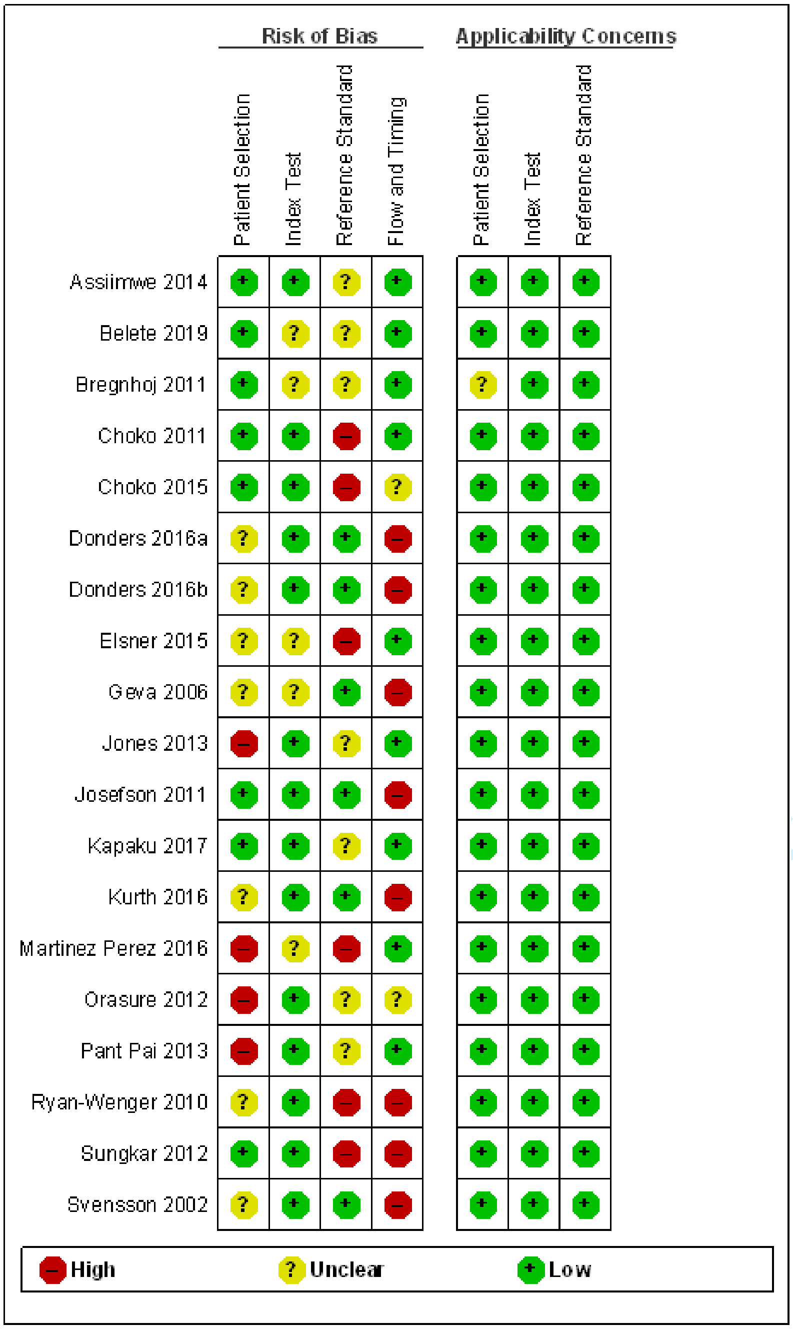

SUPPLEMENTARY MATERIAL

Supplementary Table 1: Commonly managed conditions in primary care

As reported by Cooke et al 2013 (Cooke G, Valenti L, Glasziou P, Britt H. Common general practice presentations and publication frequency. Australian Family Physician. 2013; 42:65-8)

1	Hypertension
2	Immunisation/vaccination: all
3	Acute upper respiratory tract infection
4	Depression
5	Diabetes: nongestational
6	Lipid disorders
7	General check-up
8	Osteoarthritis
9	Back complaint
10	Prescription
11	Oesophagus disease
12	Female genital check-up
13	Acute bronchitis/bronchiolitis
14	Asthma
15	Anxiety
16	Test results
17	Urinary tract infection
18	Dermatitis, contact/allergic
19	Pregnancy
20	Sleep disturbance
21	Sinusitis acute/chronic
22	Gastroenteritis
23	Vitamin/nutritional deficiency

For peer review only

24	Malignant neoplasm of skin
25	Abnormal test results
26	Atrial fibrillation/flutter
27	Oral contraception
28	Solar keratosis/sunburn
29	Ischaemic heart disease
30	Viral disease, not otherwise specified

For peer review only

Supplementary Table 2: Search strategy

**Medline**	
**#**	**Searches**
1	Diagnostic Self Evaluation/
2	((self* adj diagnos*) or selfdiagnos*).ti,ab.
3	(self* and diagnos*).ti.
4	((self* adj test*) or selftest*).ti,ab.
5	(home adj3 diagnos*).ti,ab.
6	((selfreport* or self-report*) and diagnos*).ti.
7	((selfreport* or self-report*) adj5 diagnos*).ti,ab.
8	(diagnos* and (selftreat* or self-treat*)).ti,ab.
9	1 or 2 or 3 or 4 or 5 or 6 or 7 or 8
10	respiratory tract infections/ or common cold/ or influenza, human/ or laryngitis/ or exp pharyngitis/ or rhinitis/ or exp sinusitis/ or exp supraglottitis/ or tracheitis/ or exp otitis media/
11	(((respirat* or airway*) adj2 infection*) or (common cold or influenza or flu or pharyngitis or laryngitis or tonsillitis or sore throat or rhinitis or nasopharyngitis or nasolaryngitis or sinusitis or supraglottitis or epiglottitis or tracheitis or urti or otitis media)).ti,ab.
12	exp Depressive Disorder/ or Depression/
13	(depress* or (mood adj2 disorder*)).ti,ab.
14	diabetes mellitus/ or exp diabetes mellitus, type 1/ or exp diabetes mellitus, type 2/
15	diabet*.ti,ab.
16	dyslipidemias/ or exp hyperlipidemias/
17	(dyslipid?emia? or hyperlipid?emia? or hypercholesterol?emia? or cholesterol* or triglyceride*).ti,ab.
18	hypertension/
19	(hypertens* or high blood pressure).ti,ab.
20	exp Osteoarthritis/
21	(osteoarthritis or osteo-arthritis or (degenerat* adj2 arthritis)).ti,ab.
22	back pain/ or low back pain/
23	Sciatica/
24	Intervertebral Disc Displacement/
25	((back adj2 (pain or problem? or disorder?)) or slipped disc* or sciatica).ti,ab.
26	Esophageal Diseases/ or esophageal motility disorders/ or exp gastroesophageal reflux/ or Barrett Esophagus/
27	((oesophag* or esophag*) adj2 disease).ti,ab.
28	(((oesophag* or esophag* or gastr*) adj2 reflux) or heartburn or heart burn).ti,ab.
29	((barrett* or globus) adj2 (esophag* or oesophag*)).ti,ab.
30	exp Asthma/
31	asthma*.ti,ab.

For peer review only

32	exp Anxiety Disorders/ or Anxiety/
33	(anxiety or phobia*).ti,ab.
34	exp Urinary Tract Infections/
35	exp Cystitis/
36	((urin* adj3 infection*) or bacteriuria or pyuria or cystitis).ti,ab.
37	exp Vaginitis/
38	(vaginitis or vaginosis).ti,ab.
39	dermatitis/ or exp dermatitis, contact/ or eczema/
40	(dermatitis or eczema).ti,ab.
41	exp Sleep Wake Disorders/
42	exp Sleep Apnea Syndromes/
43	((sleep adj2 (disorder* or disturbance*)) or insomnia*).ti,ab.
44	(sleep adj2 (apnea or apnoea)).ti,ab.
45	restless leg*.ti,ab.
46	Gastroenteritis/ or diarrhea/ or vomiting/
47	(gastroenteritis or (stomach adj2 (bug? or upset))).ti,ab.
48	(diarrhoea or diarrhea or food poisoning).ti,ab.
49	Sunburn/
50	(sunburn or solar keratosis).ti,ab.
51	or/10-50
52	9 and 51
53	exp "Sensitivity and Specificity"/
54	exp "REPRODUCIBILITY OF RESULTS"/
55	(sensitiv* or specific* or predict* or accura* or valid* or reproduc*).ti,ab.
56	53 or 54 or 55
57	52 and 56
**Embase**	
**#**	**Searches**
1	*Self Evaluation/
2	((self* adj diagnos*) or selfdiagnos*).ti,ab.
3	(self* and diagnos*).ti.
4	((self* adj test*) or selftest*).ti,ab.
5	(home adj3 diagnos*).ti,ab.
6	((selfreport* or self-report*) and diagnos*).ti.
7	((selfreport* or self-report*) adj5 diagnos*).ti,ab.
8	(diagnos* and (selftreat* or self-treat*)).ti,ab.
9	1 or 2 or 3 or 4 or 5 or 6 or 7 or 8
10	respiratory tract infection/ or upper respiratory tract infection/ or exp respiratory tract inflammation/ or influenza/ or otitis media/
11	(((respirat* or airway*) adj2 infection*) or (common cold or influenza or flu or pharyngitis or laryngitis or tonsillitis or sore throat or rhinitis or nasopharyngitis or nasolaryngitis or sinusitis or supraglottitis or epiglottitis or tracheitis or urti or otitis media)).ti,ab.

For peer review only

12	exp Depression/
13	(depress* or (mood adj2 disorder*)).ti,ab.
14	diabetes mellitus/ or exp diabetes mellitus, type 1/ or exp diabetes mellitus, type 2/
15	diabet*.ti,ab.
16	dyslipidemia/ or exp hyperlipidemia/
17	(dyslipid?emia? or hyperlipid?emia? or hypercholesterol?emia? or cholesterol* or triglyceride*).ti,ab.
18	hypertension/
19	(hypertens* or high blood pressure).ti,ab.
20	exp Osteoarthritis/
21	(osteoarthritis or osteo-arthritis or (degenerat* adj2 arthritis)).ti,ab.
22	backache/ or discogenic pain/ or low back pain/
23	Sciatica/
24	intervertebral disk hernia/ or intervertebral disk disease/
25	((back adj2 (pain or problem? or disorder?)) or slipped disc* or sciatica).ti,ab.
26	exp gastroesophageal reflux/ or barrett esophagus/ or esophagus disease/
27	((oesophag* or esophag*) adj2 disease).ti,ab.
28	(((oesophag* or esophag* or gastr*) adj2 reflux) or heartburn or heart burn).ti,ab.
29	((barrett* or globus) adj2 (esophag* or oesophag*)).ti,ab.
30	exp Asthma/
31	asthma*.ti,ab.
32	exp Anxiety Disorder/
33	(anxiety or phobia*).ti,ab.
34	exp Urinary Tract Infection/
35	Cystitis/
36	((urin* adj3 infection*) or bacteriuria or pyuria or cystitis).ti,ab.
37	vagina discharge/ or exp vaginitis/
38	(vaginitis or vaginosis).ti,ab.
39	dermatitis/ or contact dermatitis/ or exp eczema/
40	(dermatitis or eczema).ti,ab.
41	sleep disorder/ or exp insomnia/ or periodic limb movement disorder/
42	exp sleep disordered breathing/
43	((sleep adj2 (disorder* or disturbance*)) or insomnia*).ti,ab.
44	(sleep adj2 (apnea or apnoea)).ti,ab.
45	restless leg*.ti,ab.
46	Gastroenteritis/ or diarrhea/ or vomiting/
47	(gastroenteritis or (stomach adj2 (bug? or upset))).ti,ab.
48	(diarrhoea or diarrhea or food poisoning).ti,ab.
49	Sunburn/
50	(sunburn or solar keratosis).ti,ab.
51	or/10-50

For peer review only

52	9 and 51
53	"Sensitivity and Specificity"/
54	diagnostic accuracy/ or diagnostic test accuracy study/
55	predictive validity/ or predictive value/ or reproducibility/
56	(sensitiv* or specific* or predict* or accura* or valid* or reproduc*).ti,ab.
57	53 or 54 or 55 or 56
58	52 and 57
**Cochrane**	
**ID**	**Search**
#1	MeSH descriptor: [Diagnostic Self Evaluation] explode all trees
#2	(((self* AND diagnos*) or selfdiagnos*)):ti OR (((self* NEXT diagnos*) or selfdiagnos*)):ti,ab,kw OR (((self* NEXT test*) or selftest*)):ti,ab,kw OR (home NEAR/3 diagnos*):ti,ab,kw
#3	(((selfreport* or self-report*) and diagnos*)):ti OR (((selfreport* or self-report*) NEAR diagnos*)):ti,ab,kw OR ((diagnos* and (selftreat* or self-treat*))):ti,ab,kw
#4	#1 OR #2 OR #3
#5	MeSH descriptor: [Sensitivity and Specificity] explode all trees
#6	MeSH descriptor: [Reproducibility of Results] explode all trees
#7	(sensitiv* or specific* or predict* or accura* or valid* or reproduc*):ti,ab,kw
#8	#5 OR #6 OR #7
#9	#4 AND #8
**Cinahl**	
**#**	**Query**
S39	S3 AND S35 AND S38
S38	S36 OR S37
S37	TX sensitiv* or specific* or predict* or accura* or valid* or reproduc*
S36	(MH "Sensitivity and Specificity") OR (MH "Predictive Value of Tests")
S35	(S4 OR S5 OR S6 OR S7 OR S8 OR S9 OR S10 OR S11 OR S12 OR S13 OR S14 OR S15 OR S16 OR S17 OR S18 OR S19 OR S20 OR S21 OR S22 OR S23 OR S24 OR S25 OR S26 OR S27 OR S28 OR S29 OR S30 OR S31 OR S32 OR S33 OR S34)
S34	TI (sunburn or "solar keratosis") OR AB (sunburn or "solar keratosis")
S33	(MH "Sunburn")
S32	TI ((gastroenteritis or (stomach N2 (bug or bugs or upset)))) OR AB ((gastroenteritis or (stomach N2 (bug or bugs or upset)))) OR TI (diarrhoea or diarrhea or "food poisoning") OR AB (diarrhoea or diarrhea or "food poisoning")
S31	(MH "Gastroenteritis") OR (MH "Vomiting") OR (MH "Diarrhea")
S30	TI (((sleep N2 (disorder* or disturbance*)) or insomnia*)) OR AB (((sleep N2 (disorder* or disturbance*)) or insomnia*)) OR TI ((sleep N2 (apnea or apnoea))) OR AB ((sleep N2 (apnea or apnoea))) OR TI "restless legs" OR AB "restless legs"
S29	(MH "Sleep Disorders, Intrinsic+")
S28	TI (dermatitis or eczema) OR AB (dermatitis or eczema)
S27	(MH "Dermatitis") OR (MH "Dermatitis, Contact+") OR (MH "Eczema")
S26	TI (vaginitis or vaginosis) OR AB (vaginitis or vaginosis)

For peer review only

S25	(MH "Vaginitis+")
S24	TI (((urin* N3 infection*) or bacteriuria or pyuria or cystitis)) OR AB (((urin* N3 infection*) or bacteriuria or pyuria or cystitis))
S23	(MH "Urinary Tract Infections+") OR (MH "Cystitis")
S22	TI asthma* OR AB asthma*
S21	(MH "Asthma+")
S20	TI (((oesophag* or esophag*) N2 disease)) OR (((oesophag* or esophag*) N2 disease)) OR TI ((((oesophag* or esophag* or gastr*) N2 reflux) or heartburn or "heart burn")) OR AB ((((oesophag* or esophag* or gastr*) N2 reflux) or heartburn or "heart burn")) OR (((barrett* or globus) N2 (esophag* or oesophag*))) OR (((barrett* or globus) N2 (esophag* or oesophag*)))
S19	(MH "Gastroesophageal Reflux") OR (MH "Esophageal Motility Disorders") OR (MH "Esophageal Diseases") OR (MH "Barrett Esophagus")
S18	TI (((back N2 (pain or problem* or disorder*)) or "slipped disc*" or sciatica)) OR AB (((back N2 (pain or problem* or disorder*)) or "slipped disc*" or sciatica))
S17	(MH "Low Back Pain") OR (MH "Back Pain") OR (MH "Sciatica") OR (MH "Intervertebral Disk Displacement")
S16	TI ((osteoarthritis or osteo-arthritis or (degenerat* N2 arthritis))) OR AB ((osteoarthritis or osteo-arthritis or (degenerat* N2 arthritis)))
S15	(MH "Osteoarthritis+")
S14	TI (hypertens* or "high blood pressure") OR AB (hypertens* or "high blood pressure")
S13	(MH "Hypertension")
S12	TI (dyslipidemia* or hyperlipidemia* or hypercholesterolemia* or dyslipidaemia* or hyperlipidaemia* or hypercholesterolaemia* or cholesterol* or triglyceride*) OR AB (dyslipidemia* or hyperlipidemia* or hypercholesterolemia* or dyslipidaemia* or hyperlipidaemia* or hypercholesterolaemia* or cholesterol* or triglyceride*)
S11	(MH "Hyperlipidemia+")
S10	TI diabet* OR AB diabet*
S9	(MH "Diabetes Mellitus") OR (MH "Diabetes Mellitus, Type 1") OR (MH "Diabetes Mellitus, Type 2")
S8	TI ((depress* OR anxiety or (mood N2 disorder*))) OR AB ((depress* OR anxiety or (mood N2 disorder*)))
S7	(MH "Depression") OR (MH "Anxiety")
S6	TI ((((respirat* or airway*) N2 infection*) or ("common cold" or influenza or flu or pharyngitis or laryngitis or tonsillitis or "sore throat" or rhinitis or nasopharyngitis or nasolaryngitis or sinusitis or supraglottitis or epiglottitis or tracheitis or urti or "otitis media"))) OR AB ((((respirat* or airway*) N2 infection*) or ("common cold" or influenza or flu or pharyngitis or laryngitis or tonsillitis or "sore throat" or rhinitis or nasopharyngitis or nasolaryngitis or sinusitis or supraglottitis or epiglottitis or tracheitis or urti or "otitis media")))
S5	(MH "Otitis Media+")
S4	(MH "Respiratory Tract Infections") OR (MH "Common Cold") OR (MH "Influenza") OR (MH "Influenza, Human+") OR (MH "Laryngitis+") OR (MH "Pharyngitis") OR (MH "Rhinitis+") OR (MH "Sinusitis+") OR (MH "Tonsillitis+")

For peer review only

S3	S1 OR S2
S2	TI (((self* AND diagnos*) or selfdiagnos*)) OR AB (((self* N1 diagnos*) or selfdiagnos*)) OR TI (((self* N1 test*) or selftest*)) OR AB (((self* N1 test*) or selftest*)) OR TI (home N3 diagnos*) OR AB (home N3 diagnos*) OR TI (((selfreport* or self-report*) and diagnos*)) OR AB (((selfreport* or self-report*) N5 diagnos*)) OR TI ((diagnos* and (selftreat* or self-treat*))) OR AB ((diagnos* and (selftreat* or self-treat*)))
S1	(MH "Self Diagnosis")

For peer review only

Supplementary table 3: Data items included in extraction sheet (where available)

Study identification - author, year, location
Study research question
Study design and setting
Study funding source
Target condition definition/diagnostic criteria
Participant characteristics and numbers, including exclusions
Index test
Reference standard
Flow of participants through study including losses to follow-up<br>Patient presentation and prior testing
Conduct of the study including timing of the tests, and information on masking
Absolute counts of true positive (TP), false positive (FP), false negative (FN) and true negative (TN) diagnoses.
Statistical analyses that were performed, including whether all participants were included in analyses
Additional summary information on participant preference, timing, or cost, as available.

For peer review only

Supplementary table 4: Protocol

Accuracy of self-diagnosis in conditions commonly managed in primary care: diagnostic accuracy review

Annette Plüddemann [1], Hayley E Jones [2], Carl Heneghan [1]

1. Nuffield Department of Primary Care Health Sciences, University of Oxford, OX2 6GG
2. Population Health Sciences, Bristol Medical School, University of Bristol, BS8 2PS

Background and rationale

A wide range of conditions present to primary care, some acute, some chronic. As a consequence primary care is facing increasing workload (Hobbs et al 2016) that may become unmanageable.

Some common conditions in primary care, therefore, have the potential to be self-diagnosed and self-treated by the patients themselves. This offers superior convenience for individuals, swifter diagnosis and treatment where relevant, reduced costs for health service providers and
potentially reduce the burden on primary care services. Self-diagnosis may apply to initial diagnosis, or to diagnosing an exacerbation of an ongoing condition, such as an exacerbation of chronic obstructive pulmonary disease (COPD). In order to support self-diagnosis where safe and appropriate, the efficacy of self-diagnosis needs to be assessed, and this information used to make evidence-based decisions on who can self-diagnose safely.

Where there is an available comparison with diagnosis by a healthcare professional, it is possible to assess the accuracy of self-diagnosis. A further comparison could potentially be made with diagnosis by an allied healthcare professional such as pharmacists, but this is outside the scope of this current review. Sometimes, self-diagnosis involves using a diagnostic test (any type of medical test used to help diagnose or detect disease). For conditions where this is available and appropriate, the accuracy of these tests also informs the safety and efficacy of self-diagnosis by the patient.

Previous studies on the safety and accuracy of self-diagnosis of conditions commonly managed in primary care setting include self-diagnosis of urinary tract infection (Donofrio & Weiner 2013), high blood pressure (Tormo et al 2000) and depression (Hedayati et al 2006).

This review aims to identify, appraise and summarise the available evidence on self-diagnosis in common conditions in primary care. Cooke and colleagues recently reported the 30 most commonly managed conditions in primary care in Australia, which has a health landscape broadly comparable with western Europe (Cooke et al 2013). This list arises from survey data collected between January 2009 and December 2010, which included 194,100 patient encounters from 1,941 GPs. The most commonly managed conditions included some with the potential for self-diagnosis, e.g. urinary tract infection, as well as some that would be unsuitable for self-diagnosis, such as "general check-up". We base our review on conditions

For peer review only - http://bmjopen.bmj.com/site/about/guidelines.xhtml

from this list that are relevant for self-diagnosis. We may review infectious diseases and non-communicable diseases separately.

Objectives

Primary objective

Our primary objective is to summarize the accuracy of self-diagnosis of common conditions in primary care, compared with diagnosis by a healthcare provider.

Secondary objective

To summarise any associated relevant information relating to self-diagnosis of common conditions in primary care, such as information on patient preference, timing, or cost (only using information from studies we include for accuracy data). Where there is substantial qualitative information reported, this will only be summarised briefly; detailed qualitative approaches will not be used.

Methods

Criteria for considering studies for this review

Types of studies

Prospective or retrospective studies comparing the results of self-diagnosis of common self-limiting conditions in primary care by free-living individuals, to the results of a reference standard test performed by a healthcare service provider, will be included. Studies with a case-control design will be excluded. In case of duplicate publications we will include the study report with the highest methodological quality. There will be no language restrictions.

We will exclude studies comparing self-diagnosis with diagnosis by allied health professionals such as a pharmacists.

Participants

Adults (>= 18 years of age) self-diagnosing conditions common in primary care.

Index tests

Index tests will be the self-testing or self-diagnosis of relevant conditions, compared with diagnosis by a healthcare practitioner.

Comparator tests

Comparator tests will comprise diagnosis by a healthcare practitioner.

Outcome measures

Diagnostic accuracy measures (e.g. sensitivity, specificity, likelihood ratios, predictive values, etc.) and primary data for 2x2 tables. Studies reporting only measures of agreement will be excluded.

For peer review only

Search methods to identify studies

Electronic searches

The search strategy will be developed in consultation with a healthcare librarian experienced with supporting systematic reviews. No language restrictions will be applied. The search strategy will use multiple electronic databases, from inception onwards including:

Medline

EMBASE

Cochrane Central Register of Controlled Trials (CENTRAL)

Trip database

Web of Science for conference proceedings, dissertations, and theses

World Health Organization International Clinical Trials Registry Platform (ICTRP),

ClinicalTrials.gov

Database of Abstracts of Reviews of Effect (DARE)

We will also search Science Citation Index Expanded for study reports that cite the included studies.

The search may use relevant filters, but in order to maximise sensitivity, will not be limited to these. The reference lists of relevant studies will be examined and additional tools such as the "related articles" feature in PubMed will also be used to identify relevant publications.

Data collection and analysis

Selection of studies

Two reviewers will independently apply the selection criteria to the titles and abstracts of the study reports identified by the searches. If the decision to exclude a study cannot be made on the basis of the title and the abstract, the full study report will be retrieved for inclusion assessment. The final decision on inclusion will be based on the full study report. Disagreements between reviewers will be resolved by discussion, or if necessary by a third reviewer. Study identification will be summarised in a PRISMA flow diagram.

Data extraction and management

Two reviewers will independently extract information from selected studies into a data extraction sheet. Disagreements will be resolved by discussion, or if necessary with the help of a third reviewer.

Where this is insufficient (or unclear) information, where there is an email address provided, the authors will be contacted via email for clarification. Where data is not available for completion of 2x2 tables, the studies will be excluded from the analysis.

Data to be extracted

For peer review only

The following information will be extracted from the included studies, where available:

Study identification - author, year, location

Study research question

Study design and setting

Target condition definition/diagnostic criteria

Participant characteristics and numbers, including exclusions

Index test

Reference standard

Flow of participants through study including losses to follow-up

Patient presentation and prior testing

Conduct of the study including timing of the tests, and information on masking

Absolute counts of true positive (TP), false positive (FP), false negative (FN) and true negative (TN) diagnoses.

Statistical analyses that were performed, including whether all participants were included in analyses

Additional summary information on participant preference, timing, or cost, as available.

Assessment of methodological quality

To assess methodological quality, we will use the Quality Assessment of Diagnostic Accuracy Studies-2 (QUADAS-2) tool (Whiting et al 2011). Two reviewers will independently assess studies' methodological quality; disagreements will be resolved by discussion, or if necessary, by a third reviewer. The QUADAS-2 tool facilitates assessment of bias in four areas: patient selection; index test; reference standard; flow and timing; and also facilitates assessment of applicability of the studies to the review research question.

The data will be presented in a tables showing risk of bias and applicability within each domain assessed for each study. These data will be considered in relation to interpreting the results of the studies.

Statistical analysis and data synthesis

Analyses will be conducted for each category of condition specified. Summary tables will detail study information including the patient sample, condition, study design, the test under evaluation, and the comparator.

Meta-analysis

For each test, RevMan will be used to produce paired forest plots to explore the between-study variability of sensitivity and specificity across the included studies. For each study estimate of sensitivity and specificity, corresponding 95% confidence intervals will be shown

For peer review only

to illustrate the uncertainty related to each study estimate. If accuracy has been reported at multiple common thresholds, forest plots will be sub-grouped on threshold.

Bivariate meta-analysis methods (Reitsma et al 2005) will be used to generate pooled estimates of sensitivity and specificity where sufficient data is available for each test or condition. These will be plotted with 95% confidence and prediction ellipses in Receiver Operating Characteristic (ROC) space. Where appropriate, summary ROC curves will also be plotted, drawing on the equivalence of the bivariate method and the hierarchical summary ROC meta-analysis model (Rutter and Gatsonis 2001; Harbord et al 2007). For these analyses, we will use WinBUGS or the metandi command in Stata, as appropriate, and feed parameters directly into Revman to produce Cochrane-standardised output.

Where appropriate, meta-analysis models that include multiple thresholds will be employed (e.g. Steinhauser et al 2016 or similar).

Investigating heterogeneity

For medical conditions for which data from more than one study are available, it may be possible to investigate heterogeneity in the results. Two approaches will be used to explore the sources of between-study heterogeneity: 1) inclusion of study level characteristics as covariates in the bivariate model (meta-regression) 2) carrying out sub-group analyses. These approaches will only be carried out if there is sufficient data available and sub-group specific pooled estimates are thought to be of clinical relevance. Any meta-regressions will be carried out using WinBUGS or the xtmelogit command in Stata.

Sensitivity analyses

If there appear to be any outliers in the data, these studies will be removed from the analysis to evaluate the impact on the overall pooled estimates.

Investigating reporting bias

Funnel plots used to detect publication bias in reviews of RCTs have been shown to be misleading for diagnostic test accuracy reviews (Deeks et al 2005; Leeflang et al 2008). Funnel plots as an assessment of reporting bias will therefore not be included in this review. Publication bias will be assessed using Deek’s test, as recommended by the Cochrane Handbook, where data allows (Deeks et al 2005).

Funding

This project is funded by National Institute for Health Research School for Primary Care Research (NIHR SPCR) [ProjectNumber 390]

Declarations of conflict of interest

Dr. Plüddemann reports grants from NIHR, grants from NIHR School of Primary Care Research, during the conduct of the study; and occasionally receives expenses for teaching Evidence-Based Medicine. Dr. Heneghan reports receiving expenses and fees for his media work. He has received expenses from the WHO and holds grant funding from the NIHR, the NIHR School of Primary Care Research, The Wellcome Trust and the WHO. He has received financial remuneration from an asbestos case. He has also received income from the

For peer review only

publication of a series of toolkit books published by Blackwells. On occasion, he receives expenses for teaching EBM and is also paid for his GP work in NHS out of hours. CEBM jointly runs the EvidenceLive Conference with the BMJ and the Overdiagnosis Conference with some international partners which are based on a non-profit making model.

References

Cooke G, Valenti L, Glasziou P, Britt H. Common general practice presentations and publication frequency. Aust Fam Physician. 2013;42(1-2):65-8.

Deeks JJ, Macaskill P, Irwig L. The performance of tests of publication bias and other sample size effects in systematic reviews of diagnostic test accuracy was assessed. J Clin Epidemiol. 2005;58(9):882-93

Donofrio JC, Weiner SG. Female patient self-diagnosis compared with emergency physician diagnosis of urinary tract infection. J Emerg Med. 2013;45(6):969-73. doi: 10.1016/j.jemermed.2013.07.019

Harbord RM, Deeks JJ, Egger M, Whiting P, Sterne JA. A unification of models for meta-analysis of diagnostic accuracy studies. Biostatistics. 2007 Apr;8(2):239-51.

Hedayati SS, Bosworth HB, Kuchibhatla M, Kimmel PL, Szczech LA. The predictive value of self-report scales compared with physician diagnosis of depression in hemodialysis patients. Kidney Int. 2006 May;69(9):1662-8.

Hobbs FDR, Bankhead C, Mukhtar T, Stevens S, Perera-Salazar R, Holt T, Salisbury C; National Institute for Health Research School for Primary Care Research. Clinical workload in UK primary care: a retrospective analysis of 100 million consultations in England, 2007–14. The Lancet; 387; 10035: 2323-2330

Leeflang MM, Deeks JJ, Gatsonis C, Bossuyt PM; Cochrane Diagnostic Test Accuracy Working Group. Systematic reviews of diagnostic test accuracy. Ann Intern Med. 2008; 16;149(12): 889-97

Rutter CM, Gatsonis CA. A hierarchical regression approach to meta-analysis of diagnostic test accuracy evaluations. Stat Med. 2001 Oct 15;20(19):2865-84.

Reitsma JB, Glas AS, Rutjes AW, Scholten RJ, Bossuyt PM, Zwinderman AH. Bivariate analysis of sensitivity and specificity produces informative summary measures in diagnostic reviews. J Clin Epidemiol. 2005 Oct;58(10):982-90

Review Manager (RevMan) [Computer program]. Version 5.3. Copenhagen: The Nordic Cochrane Centre, The Cochrane Collaboration, 2014

Steinhauser S, Schumacher M, Rücker G. Modelling multiple thresholds in meta-analysis of diagnostic test accuracy studies. BMC Med Res Methodol. 2016 Aug 12;16(1):97. doi: 10.1186/s12874-016-0196-1.

Tormo MJ, Navarro C, Chirlaque MD, Barber X. Validation of self diagnosis of high blood pressure in a sample of the Spanish EPIC cohort: overall agreement and predictive values. EPIC Group of Spain. J Epidemiol Community Health. 2000 Mar;54(3):221-6.

For peer review only

Supplementary Table 5: Characteristics of included studies

Disease/ condition	Author, year	Design	Setting	Country	Study duration	Number of participants	Mean age* (years)	Female (%)
Vaginal infection	Donders 2016	Prospective diagnostic accuracy study	Birth control, general gynaecology, infertility and prenatal clinics	Uganda	N/A	360	28.3	100
	Sungkar 2012	RCT (intervention arm only)	Prenatal clinics or hospitals	Indonesia	24 weeks	176	28	100
	Geva 2006	Prospective diagnostic accuracy study	Gynaecologic clinics	Israel	N/A	593	18 – 60 range	100
	Ryan-Wenger 2010	Prospective diagnostic accuracy study	Military clinics	USA	N/A	546	25.7	100
	Jones 2013	RCT (both arms)	Clinics or home	Brazil	N/A	695	18 – 40 range	100
Common skin condition	Bregnhoj 2011	Prospective cohort	Not reported	Denmark	18 months	502	17.5	95
	Svensson 2002	Prospective diagnostic accuracy study	Dermatology outpatient clinics	Sweden	N/A	208	40.4	50
	Elsner 2015	Prospective diagnostic accuracy study	Not reported	Germany & Austria	N/A	165	≥18 years	81
	Josefson 2011	Prospective diagnostic accuracy study	Hospital dermatology departments	Sweden	N/A	243	44	69
HIV	Assiimwe 2014	RCT (unsupervised arm only)	Home	Uganda	N/A	123	28 (23–32), median (IQR)	47
	Belete 2019	Cross-sectional	Public health facilities	Ethiopia	N/A	400	29 (17.7–40.3), median (IQR)	61
	Choko 2011	Cross-sectional	Home	Malawi	N/A	241	27 (NR) median (IQR)	52
	Choko 2015	RCT (intervention arm only)	Home	Malawi	2 years	2370	NR	NR

For peer review only

	Kapaku 2017	Cohort	Home/Voluntary counselling & testing facilities	Zambia	1 year	2572	26 (21–35), median (IQR)	59
	Kurth 2016	Cross-sectional	NR (not at home)	Kenya	N/A	240**	36	33
	Martinez Perez 2016	Cross-sectional	Health care clinics/HIV testing sites	South Africa	N/A	2205	Male: 27 (11-36) Female: 28 (22-36) median (IQR)	66
	Orasure 2012	Cross-sectional	Study site	USA	N/A	5798	NR	50
	Pant Pai 2013	Cross-sectional	Hospital	South Africa	N/A	251	≥18 years	79

RCT – randomised control trial; N/A – not applicable; NR – not reported, * unless otherwise indicated, ** Subset (N=113) that used laboratory reference standard included in systematic review and pooled analysis.

For peer review only

Supplementary Table 6: Characteristics of self-diagnosis (index) tests and reference standard tests

For peer review only

Disease/ condition	Author, year	Test for	Self-diagnosis test (index)	Self-diagnosis test threshold	Reference test	Reference test threshold	Interval between index & reference test	Data collection points
Vaginal infection	Donders 2016a	Bacterial vaginosis	Vaginal fluid test using pH strip	+/- (≥ 4.5 pH)	Air dried vaginal fluid test using gram staining (Nugent score) Assessment by central laboratory	+/- (≥ 4.5 pH, Nugent score 7-10)	NR	N/A
	Donders 2016b			+/- (≥ 4.7 pH)		+/- (≥ 4.7 pH, Nugent score 7-10)		
	Sungkar 2012	Bacterial vaginosis	Vaginal fluid test using pH strip	+/-	Vaginal fluid test using microbiological gram staining test (Kopeloff modified Gram stain) Prepared by midwives for laboratory assessment	+/-	NR	Baseline, 16 – 18, 18 – 20, 20 – 22, 22 – 24 weeks
	Geva 2006	Bacterial vaginosis and/or *Trichomonas vaginalis*	Vaginal discharge test using panty liner test kit (VI-SENSE)	+/- (based on strip colour, no level reported*)	Clinical diagnosis plus vaginal wall swabs testing pH (nitrazine paper), amine, culture (InPouch TV, BioMed Diagnostic) and gram staining (Nugent score). Assessment by board certified gynaecologists and central laboratory.	BV: >3: (a) homogeneous discharge, (b) pH value >4.5, (c) release of fishy odor (KOH was added to the vaginal discharge, and (d) presence of clue cells; or 7+ Nugent score of gram stain TV: +/- by culture	Within 6 hours	N/A
	Ryan-Wenger 2010	Bacterial vaginosis and/or *Trichomonas vaginalis*	Vaginal fluid test using Women in the military self-diagnosis kit	+/- (based on ≥ 4.7 pH, amines,	Clinical interview plus vaginal fluid test for pH (nitrazine paper), amines (FemExam card), whiff, wet mount	+/-	None	N/A

			(includes FemExam card)	vaginal itching)	microscopy (Affirm VPIII Microbial Identification Test)<br>Assessment by women's health nurse practitioner plus in clinic microscopy testing			
	Ryan-Wenger 2010	*Candida* vaginitis	Vaginal fluid test using Women in the military self-diagnosis kit (includes FemExam card)	+/-<br>(based on ≥ 4.7 pH, amines, vaginal itching)	Clinical interview plus vaginal fluid test for pH (nitrazine paper, amines (FemExam card), whiff, wet mount microscopy (Affirm VPIII Microbial Identification Test)<br>Assessment by women's health nurse practitioner plus in clinic microscopy testing	+/-	None	N/A
	Jones 2013	*Trichomonas vaginalis*	Vaginal fluid testing using dipstick test (OSOM Trichomonas rapid test)	+/-<br>(two red lines)	Vaginal fluid test using PCR test<br>Assessment at central laboratory	+/-	None	N/A
Common skin condition	Bregnhoj 2011	Eczema	Questionnaire on presence of eczema	Positive response	Hand Eczema Severity Index (HECSI)<br>Interpretation by clinician	+ve for presence of eczema	Same day	Inclusion & 18 month follow-up
	Svensson 2002	Hand eczema	Questionnaire on presence of eczema	Positive response	Hand examination for erythema, papules, vesicles, scaling, fissures, lichenification and hyperkeratotic areas.<br>Assessment by experienced dermatologist	+ve if erythema and papules / vesicles OR erythema and scaling and fissures / lichenification	None	N/A
	Elsner 2015	Allergic reaction to nickel and fragrance	Irritant reaction to surgical tapes on upper arm after 48 hours	+/-	Irritant reaction to surgical tapes on upper arm after 48 hours<br>Assessment by clinician	+/-	None	N/A
	Josefson 2011	Allergic reaction to nickel	Medical plaster patches (Nixema) on upper arm with	+/-	Medical plaster patches (Finn	+/-	Same day	N/A

For peer review only - http://bmjopen.bmj.com/site/about/guidelines.xhtml

			readings on days 3-4		chambers® on Scanpor® tape or IQ Ultra® Chambers) on back with readings on days 3-4 and/or day 7 Assessment by dermatologist			
HIV	Assiimwe 2014	Antibodies for HIV1 and HIV2	Oral fluid test using OraQuick® In-Home Rapid HIV-1/2 Antibody Test (Orasure Technologies)	+/-	Finger pick blood test. Nationally approved algorithm of POC rapid HIV tests (Determine (Abbot Laboratories), STAT-PAK (Chembio Diagnostic Systems Inc) and Unigold (Trinity Biotech plc) as a tiebreaker). Assessment by research assistants.	+/-	12 -72 hours	N/A
	Belete 2019	Antibodies for HIV1 and HIV2	Oral fluid test using OraQuick® Rapid HIV-1/2 Antibody Test (Orasure Technologies)	+/-	Finger pick blood test. Nationally approved algorithm of POC rapid HIV tests (Wanti (Beijing), Unigold (Trinity Biotech plc), Vikia). Assessment by health professional.	+/-	Same time	N/A
	Choko 2011	Antibodies for HIV1 and HIV2	Oral fluid test using OraQuick ADVANCE® Rapid HIV-1/2 Antibody Test	+/-	Finger pick blood test. Algorithm of POC rapid HIV tests (Determine (Abbot Laboratories), Unigold (Trinity Biotech plc) and SD Bioline HIV i/II (Standard Diagnostics, Inc.) as a tiebreaker). Assessment by counsellor.	+/-	Same time	N/A
	Choko 2015	Antibodies for HIV1 and HIV2	Oral fluid test using OraQuick ADVANCE® Rapid HIV-1/2 Antibody Test	+/-	Finger pick blood test. Two parallel POC rapid HIV tests (Determine (Abbot Laboratories), Unigold (Trinity Biotech plc)). Assessment by nurse.	+/-	Approx. 1 week	1 – 12, 13 – 24 months (max. 1 test in 12 months)

For peer review only

	Kapaku 2017	Antibodies for HIV1 and HIV2	Oral fluid test using OraQuick® rapid HIV-1/2 Antibody Test (Orasure Technologies)	+/-	Venous blood test (EDTA tube) Testing in certified central laboratory (Abbott Architect HIV1 Ag/Ab combo assay, positive results confirmed by BioRad GS HIV combo Ag/Ab assay)	+/-	Within 8 hours	Once in 12 month study period.
	Kurth 2016	Antibodies for HIV1 and HIV2	Oral fluid test using OraQuick ADVANCE® Rapid HIV-1/2 Antibody Test	+/-	Venous blood test. ELISA testing by single person in certified central laboratory (Vironostika HIV Uni-Form II Ag/Ab (bioMe´rieux Inc.))	+/-	Within 8 hours	N/A
	Martinez Perez 2016	Antibodies for HIV1 and HIV2	Oral fluid test using OraQuick ADVANCE® Rapid HIV-1/2 Antibody Test	+/-	Finger pick blood test. POC rapid HIV tests (Determine (Abbot Laboratories), confirmatory Unigold (Trinity Biotech plc)). Assessment by HIV counsellor.	+/-	Same time	N/A
	Orasure 2012	Antibodies for HIV1 and HIV2	Oral fluid test using OraQuick® In-Home Rapid HIV-1/2 Antibody Test (Orasure Technologies)	+/-	Venous blood test. FDA approved serum EIA and Western blot in FDA approved laboratory.	+/-	Unclear	N/A
	Pant Pai 2013	Antibodies for HIV1 and HIV2	Oral fluid test using OraQuick® rapid HIV-1/2 Antibody Test (Orasure Technologies)	+/-	Venous blood test. ELISA with p24 Antigen tests in reference laboratories all within 24 hours (Architect HIV Ag/Ab combo (Abbott Laboratories), positive results confirmed by Western Blot)	+/-	Same time	N/A

HIV - Human immunodeficiency virus, POC – Point of care, max. – maximum, FDA – Food and Drug Administration, USA, N/A – not applicable; NR – not reported; KOH – potassium hydroxide; PCR – polymerase chain reaction; * whilst no reported level was given for VI-SENSE, it was reported that the polymer used in this product had a range of 4.3 – 5.1 pH

For peer review only

PRISMA 2020 Checklist

Section and Topic	Item #	Checklist item	Location where item is reported
**TITLE**			
Title	1	Identify the report as a systematic review.	Title page (pg 1)
**ABSTRACT**			
Abstract	2	See the PRISMA 2020 for Abstracts checklist.	Page 2
**INTRODUCTION**			
Rationale	3	Describe the rationale for the review in the context of existing knowledge.	Introduction (pg 4)
Objectives	4	Provide an explicit statement of the objective(s) or question(s) the review addresses.	Introduction (pg 2)
**METHODS**			
Eligibility criteria	5	Specify the inclusion and exclusion criteria for the review and how studies were grouped for the syntheses.	Methods, Types of studies (pg 4) & Statistical analysis and data analysis (pg 5)
Information sources	6	Specify all databases, registers, websites, organisations, reference lists and other sources searched or consulted to identify studies. Specify the date when each source was last searched or consulted.	Methods, Search methods to identify studies (pg 4)
Search strategy	7	Present the full search strategies for all databases, registers and websites, including any filters and limits used.	Supplementary table 1
Selection process	8	Specify the methods used to decide whether a study met the inclusion criteria of the review, including how many reviewers screened each record and each report retrieved, whether they worked independently, and if applicable, details of automation tools used in the process.	Methods, Selection of studies (pg 5). No automation tools used.
Data collection process	9	Specify the methods used to collect data from reports, including how many reviewers collected data from each report, whether they worked independently, any processes for obtaining or confirming data from study investigators, and if applicable, details of automation tools used in the process.	Methods, Data extraction and management (pg 5). No automation tools used.
Data items	10a	List and define all outcomes for which data were sought. Specify whether all results that were compatible with each outcome domain in each study were sought (e.g. for all measures, time points, analyses), and if not, the methods used to decide which results to collect.	Supplementary table 2. Methods, Data extraction and

For peer review only

PRISMA 2020 Checklist

Section and Topic	Item #	Checklist item	Location where item is reported
			management (pg 5).
	10b	List and define all other variables for which data were sought (e.g. participant and intervention characteristics, funding sources). Describe any assumptions made about any missing or unclear information.	Supplementary table 2.<br>Methods, Data extraction and management (pg 5).
Study risk of bias assessment	11	Specify the methods used to assess risk of bias in the included studies, including details of the tool(s) used, how many reviewers assessed each study and whether they worked independently, and if applicable, details of automation tools used in the process.	Methods, Assessment of methodological quality (pg 5).<br>No automation tools used.
Effect measures	12	Specify for each outcome the effect measure(s) (e.g. risk ratio, mean difference) used in the synthesis or presentation of results.	Methods, Statistical analysis and data synthesis (pg 5)<br>Supplementary table 2
Synthesis methods	13a	Describe the processes used to decide which studies were eligible for each synthesis (e.g. tabulating the study intervention characteristics and comparing against the planned groups for each synthesis (item #5)).	Methods, Statistical analysis and data synthesis (pg 5)
	13b	Describe any methods required to prepare the data for presentation or synthesis, such as handling of missing summary statistics, or data conversions.	N/A Studies included providing 2x2 published data.
	13c	Describe any methods used to tabulate or visually display results of individual studies and syntheses.	Methods, Statistical analysis and data synthesis (pg 5)
	13d	Describe any methods used to synthesize results and provide a rationale for the choice(s). If meta-analysis was performed, describe the model(s), method(s) to identify the presence and extent of statistical heterogeneity, and software package(s) used.	Methods, meta-analysis (pg 5)
	13e	Describe any methods used to explore possible causes of heterogeneity among study results (e.g. subgroup analysis, meta-regression).	Methods, Investigating

For peer review only

PRISMA 2020 Checklist

Section and Topic	Item #	Checklist item	Location where item is reported
			heterogeneity (pg 5 & 6)
	13f	Describe any sensitivity analyses conducted to assess robustness of the synthesized results.	Results, Self-diagnosis of HIV (pg 9)
Reporting bias assessment	14	Describe any methods used to assess risk of bias due to missing results in a synthesis (arising from reporting biases).	Not examined.
Certainty assessment	15	Describe any methods used to assess certainty (or confidence) in the body of evidence for an outcome.	Methods, meta-analysis (pg 5)
**RESULTS**			
Study selection	16a	Describe the results of the search and selection process, from the number of records identified in the search to the number of studies included in the review, ideally using a flow diagram.	Results (pg 6) Figure 1
	16b	Cite studies that might appear to meet the inclusion criteria, but which were excluded, and explain why they were excluded.	N/A. Rather 2 studies were included that were borderline exclusion (pg 7 & 8)
Study characteristics	17	Cite each included study and present its characteristics.	Results (pg 6 – 9). Supplementary tables 4 & 5
Risk of bias in studies	18	Present assessments of risk of bias for each included study.	Figure 5
Results of individual studies	19	For all outcomes, present, for each study: (a) summary statistics for each group (where appropriate) and (b) an effect estimate and its precision (e.g. confidence/credible interval), ideally using structured tables or plots.	Figures 2 - 4
Results of syntheses	20a	For each synthesis, briefly summarise the characteristics and risk of bias among contributing studies.	Results (pg 6-9)
	20b	Present results of all statistical syntheses conducted. If meta-analysis was done, present for each the summary estimate and its precision (e.g. confidence/credible interval) and measures of statistical heterogeneity. If comparing groups, describe the direction of the effect.	Results, Self-diagnosis of HIV (pg 8 & 9). Figure 4
	20c	Present results of all investigations of possible causes of heterogeneity among study results.	Results, Self-diagnosis of HIV (pg 8 & 9). Figure 4
	20d	Present results of all sensitivity analyses conducted to assess the robustness of the synthesized results.	Results, Self-

For peer review only

PRISMA 2020 Checklist

Section and Topic	Item #	Checklist item	Location where item is reported
			diagnosis of HIV (pg 9). Figure 4
Reporting biases	21	Present assessments of risk of bias due to missing results (arising from reporting biases) for each synthesis assessed.	N/A
Certainty of evidence	22	Present assessments of certainty (or confidence) in the body of evidence for each outcome assessed.	Discussion (pg 10 & 11)
**DISCUSSION**			
Discussion	23a	Provide a general interpretation of the results in the context of other evidence.	Discussion (pg 10 & 11)
	23b	Discuss any limitations of the evidence included in the review.	Discussion (pg 10 & 11)
	23c	Discuss any limitations of the review processes used.	Discussion (pg 10 & 11)
	23d	Discuss implications of the results for practice, policy, and future research.	Discussion (pg 11)
**OTHER INFORMATION**			
Registration and protocol	24a	Provide registration information for the review, including register name and registration number, or state that the review was not registered.	Abstract (pg 2). Methods (pg 6)
	24b	Indicate where the review protocol can be accessed, or state that a protocol was not prepared.	Supplementary table 3
	24c	Describe and explain any amendments to information provided at registration or in the protocol.	Methods, meta-analysis (pg 5). Results, self-diagnosis of HIV (post-hoc analysis, pg 9)
Support	25	Describe sources of financial or non-financial support for the review, and the role of the funders or sponsors in the review.	Funding statement (pg 11)
Competing interests	26	Declare any competing interests of review authors.	Competing interests statement (pg 11)
Availability of data, code and other materials	27	Report which of the following are publicly available and where they can be found: template data collection forms; data extracted from included studies; data used for all analyses; analytic code; any other materials used in the review.	Data availability statement (pg 11)

For peer review only

PRISMA 2020 Checklist

From: Page MJ, McKenzie JE, Bossuyt PM, Boutron I, Hoffmann TC, Mulrow CD, et al. The PRISMA 2020 statement: an updated guideline for reporting systematic reviews. BMJ 2021;372:n71. doi: 10.1136/bmj.n71
For more information, visit: http://www.prisma-statement.org/

For peer review only
